# Zinc(II) Complex with Pyrazolone-Based Hydrazones is Strongly Effective against *Trypanosoma brucei* Which Causes African Sleeping Sickness

**DOI:** 10.1021/acs.inorgchem.2c02201

**Published:** 2022-08-15

**Authors:** Fabio Marchetti, Alessia Tombesi, Corrado Di Nicola, Riccardo Pettinari, Federico Verdicchio, Alessandra Crispini, Francesca Scarpelli, Cecilia Baldassarri, Elisa Marangoni, Anders Hofer, Agustín Galindo, Riccardo Petrelli

**Affiliations:** † Chemistry Interdisciplinary Project (CHIP), School of Science and Technology, University of Camerino, via Madonna delle Carceri, 62032 Camerino, Macerata, Italy; ‡ Chemistry Interdisciplinary Project (CHIP), School of Pharmacy, University of Camerino, via Madonna delle Carceri, 62032 Camerino, Macerata, Italy; § MAT-InLAB, Dipartimento di Chimica e Tecnologie Chimiche, Università della Calabria, 87036 Arcavacata di Rende, Cosenza, Italy; ∥ Department of Medical Biochemistry and Biophysics, Umea University, 901 87 Umeå, Sweden; ⊥ Departamento de Química Inorganíca, Facultad de Química, Universidad de Sevilla, Aptdo 1203, 41071 Sevilla, Spain

## Abstract

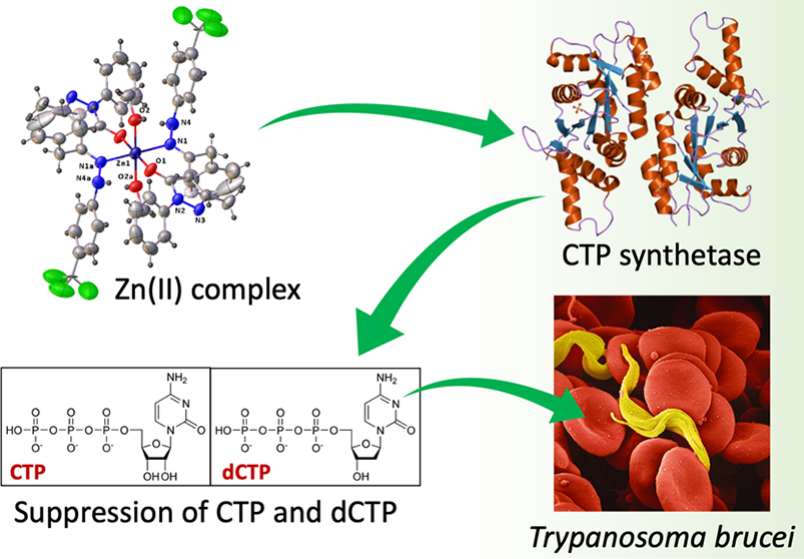

Two pyrazolone-based hydrazones H_2_L′ [in general, H_2_L′; in detail, **H**_**2**_**L**^**1**^ = 5-methyl-2-phenyl-4-(2-phenyl-1-(2-(4-(trifluoromethyl)phenyl)hydrazineyl)ethyl)-2,4-dihydro-3*H*-pyrazol-3-one, **H**_**2**_**L**^**2**^ = (*Z*)-5-methyl-2-phenyl-4-(2-phenyl-1-(2-(pyridin-2-yl)hydrazineyl)ethylidene)-2,4-dihydro-3*H*-pyrazol-3-one] were reacted with Zn(II) and Cu(II) acceptors affording the complexes [Zn(HL^1^)_2_(MeOH)_2_], [Cu(HL^1^)_2_], and [M(HL^2^)_2_] (M = Cu or Zn). X-ray and DFT studies showed the free proligands to exist in the N–H,N–H tautomeric form and that in [Zn(HL^1^)_2_(MeOH)_2_], zinc is six-coordinated by the N,O-chelated (HL^1^) ligand and other two oxygen atoms of coordinated methanol molecules, while [Cu(HL^1^)_2_] adopts a square planar geometry with the two (HL^1^) ligands in anti-conformation. Finally, the [M(HL^2^)_2_] complexes are octahedral with the two (HL^2^) ligands acting as κ-O,N,N-donors in planar conformation. Both the proligands and metal complexes were tested against the parasite *Trypanosoma brucei* and Balb3T3 cells. The Zn(II) complexes were found to be very powerful, more than the starting proligands, while maintaining a good safety level. In detail, **H**_**2**_**L**^**1**^ and its Zn(II) complex have high selective index (55 and >100, respectively) against *T. brucei* compared to the mammalian Balb/3T3 reference cells. These results encouraged the researchers to investigate the mechanism of action of these compounds that have no structural relations with the already known drugs used against *T. brucei*. Interestingly, the analysis of NTP and dNTP pools in *T. brucei* treated by **H**_**2**_**L**^**1**^ and its Zn(II) complex showed that the drugs had a strong impact on the CTP pools, making it likely that CTP synthetase is the targeted enzyme.

## Abstract 2

New Zn(II) and Cu(II) complexes with pyrazolone-based hydrazone ligands display different structural features. The Zn(II) complexes show strong efficiency against the parasite *Trypanosoma brucei*, while maintaining a good safety level. They strongly impact the CTP pools, indicating that CTP synthetase is the targeted enzyme.

## Introduction

**Figure 1:**
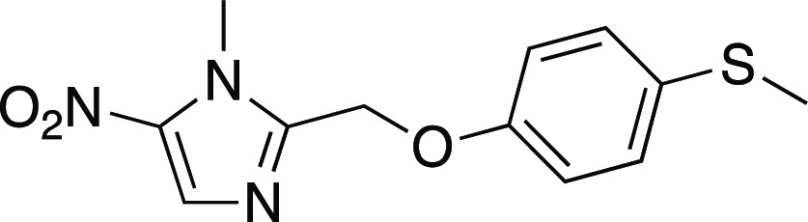
Fexinidazole.

Parasitic protozoal diseases, including trypanosomiasis, are listed by the World Health Organization (WHO) as part of 17 neglected tropical diseases which are defined as “a diverse group of communicable diseases that prevail in tropical and subtropical conditions”.^1^ These diseases are referred to as “neglected” primarily because there is no financial incentive to develop drugs for a patient population that cannot afford them. Consequently, there are few or no reason for “for-profit” companies to invest in research and development of drugs that will not result in high financial returns. Therefore, much of the drug discovery and hit-to-lead optimization for these diseases is performed in academic laboratories without the financial, personnel, and technical resources of a pharmaceutical company. To make matters worse, the absence of vaccines and in some cases, the emergence of resistant parasite strains underlines the importance of the successful track record of antiprotozoal drug discovery. The disease is characterized first by a hemolymphatic stage (early stage or stage 1), in which parasite is present in the blood and in the lymphatic system and patients present general flu-like symptoms. In the second or late stage (meningo-encephalic stage or stage 2), parasites will penetrate the blood–brain barrier, invading the perivascular areas with subsequent infiltration in the white and gray matter of the brain. Only a few drugs are effective and registered so far for the treatment of human African trypanosomiasis (HAT), such as suramin and pentamidine, and all of them have a certain level of toxicity.^2−4^ More recently, another drug, named fexinidazole, has been discovered as effective against HAT (Figure 1) and listed as essential medicine by WHO.^3^

It is effective both in early and in late stage of the disease, differently from suramin, which is used only in the early stage of HAT.^4−6^ Although it delivers all these advantages, fexinidazole shows a significant toxicity profile: neutropenia, body weight loss, reduction in food intake, psychotic disorders, tremors, and dizziness. Therefore, novel scaffolds or new drug entities are urgently needed.

Hydrazones and their metal complexes have been thoroughly investigated over decades for their antioxidant, anti-inflammatory, anticonvulsant, analgesic, antimicrobial, antiparasitic, antitubercular, anti-HIV, and anticancer behavior, raising great interest in the field of medicinal chemistry.^7−9^

It is known that pyrazolone also is a structural motif with a broad-spectrum of pharmacological features, including antimicrobial, antitumor, anti-inflammatory, antioxidant, antitubercular, antiviral, lipid-lowering, antihyperglycemic, and protein inhibitory activities.^10^ Previous studies of our research team underlined the wide biological activities of a series of Zn(II) complexes with acylpyrazolones, displaying antiproliferative activity against human breast cancer cells,^11^ and of pyrazolone-based hydrazones and their ruthenium(II) complexes as anticancer multitarget agents.^12,13^ Moreover, some Cu(II) and Zn(II) complexes with acylpyrazolones and acylhydrazone-5-pyrazolones were recently reported and found to display *in vitro* antimalarial activity with considerable inhibitory effects against *Plasmodium falciparum*.^14−16^ Based on these findings, two new pyrazolone-based hydrazones and their corresponding Zn(II) and Cu(II) complexes have been synthetized and screened against *T. brucei* and mammalian Balb/3T3 cells. The most active and selective metal complex containing a Zn(II) center was further investigated to identify the mechanism of action and the possible target, focusing the study on the peculiar nucleotide metabolism of *T. brucei*. In particular, it is not able to synthesize purines and must recover them from the host,^17^ while concerning pyrimidine synthesis and metabolism,^18^ the parasites behave normally for uridine nucleotides and in a similar way for cytidine nucleotides since they are produced through a unique *de novo* pathway involving the CTP synthetase (CTPS) enzyme inside the parasite. The complete dependence on CTPS for the production of CTP makes the trypanosomes vulnerable to inhibitors of this enzyme.^19^

## Results and Discussion

**Scheme 1:**
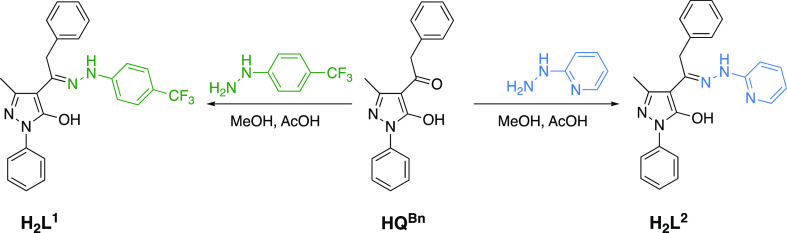
Synthesis of Proligands **H**_**2**_**L**^**1**^ and **H**_**2**_**L**^**2**^ from HQ^Bn^

**Chart 1:**
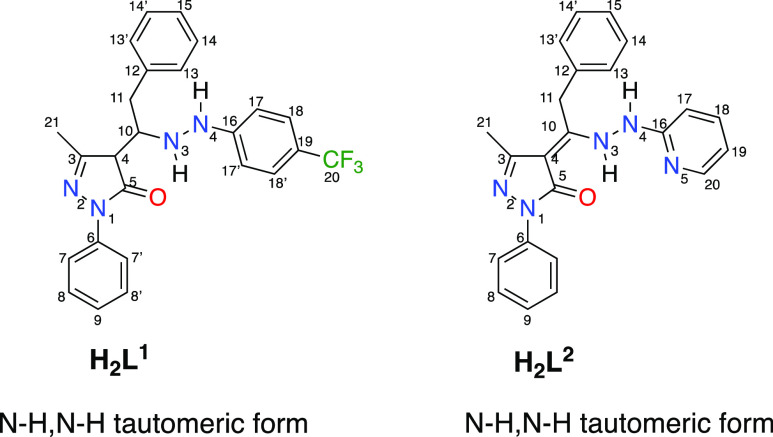
Solid-State Structure of **H**_**2**_**L**^**1**^ and **H**_**2**_**L**^**2**^ with Numbered C and N Atoms

The proligand precursor is 1-(5-hydroxy-3-methyl-1-phenyl-1*H*-pyrazol-4-yl)-2-phenylethanone, HQ^Bn^, which was synthesized as previously reported.^20^ The proligands H_2_L′ were prepared by reacting an equimolar amount of HQ^Bn^ and the appropriate hydrazine, in detail 1-(4-trifluoromethylphenyl)hydrazine, to give **H**_**2**_**L**^**1**^, or 2-hydrazinopyridine, affording **H**_**2**_**L**^**2**^, in methanol-containing traces of glacial acetic acid, according to the reported procedure (Scheme 1).^21^ The two proligands are air-stable in the solid-state and soluble in most organic solvents. In their solid state IR spectra, strong bands in the range of 1618–1614 cm^–1^ were assigned to ν(>C=N−) of the azomethine fragment, while those in the range of 1593–1532 cm^–1^ were assigned to ν(C=N) of the pyrazole ring and, for **H**_**2**_**L**^**2**^, also of the pyridine ring.^22−25^ Additional strong bands at *ca.* 1320 and 1100 in the IR spectra of **H**_**2**_**L**^**1**^ were assigned to asymmetric and symmetric stretching vibrations of the CF_3_ group,^26^ while those falling in the range of 1009–1064 cm^–1^ are typical of ν(N–N).^12^ Based on the band found at 3211 due to ν(N–H) and on the broad absorption in the range of 3130–2700 cm^–1^, which is typical of ν(N–H···O) involved in intramolecular H-bonding, proligand **H**_**2**_**L**^**1**^ was concluded to exist in the N–H,N–H tautomeric form, as indicated in Chart 1, as further confirmed by the X-ray study below. Similarly, the broad band centered at 3301 cm^–1^ in the IR spectrum of **H**_**2**_**L**^**2**^ is in accordance with a N–H,N–H tautomeric form (Chart 1), differently from an analogous pyridine-containing proligand which was isolated in a N–H,N–H zwitterionic form.^12^ The ^1^H and ^13^C NMR chemical shifts were assigned based on ^1^H–^1^H and one-bond and long-range ^1^H–^13^C couplings, seen in{^1^H–^1^H}-COSY, {^1^H–^13^C}-HSQC, and {^1^H–^13^C}-HMBC (see Supporting Information). Moreover, indirect ^15^N NMR chemical shifts were assigned based on {^1^H–^15^N}-HSQC and {^1^H–^15^N}-HMBC for the free proligands **H**_**2**_**L**^**1**^ and **H**_**2**_**L**^**2**^, giving further support to the presence also in solution of the N–H,O–H tautomeric forms. The metal complexes **1–4** were prepared by reacting metal acetate hydrate and the appropriate proligand in methanol.

**Scheme 2:**
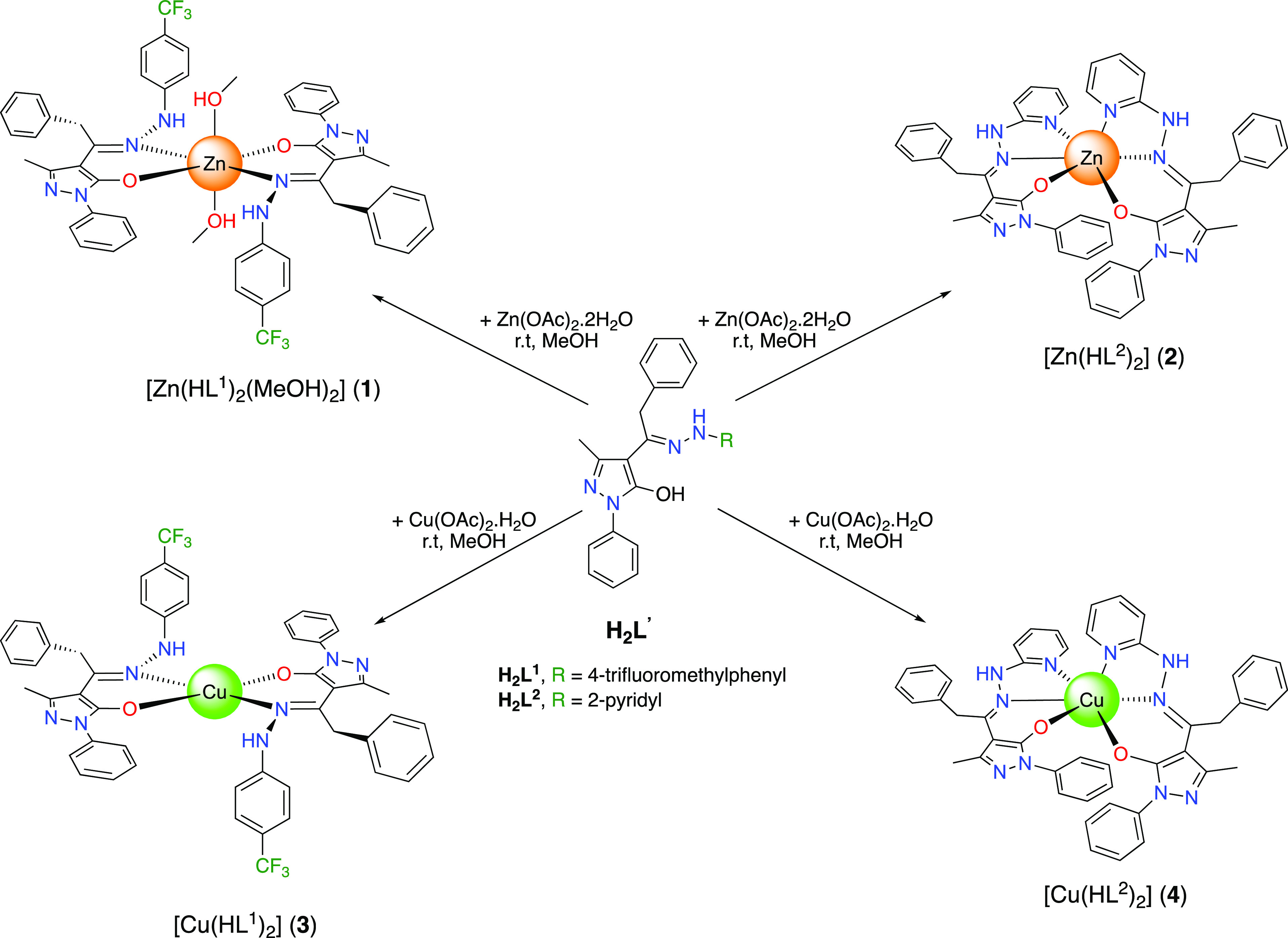
Synthetic Procedures of Complexes **1–4**

The basic anionic forms of (HL^1^) coordinate Zn(II) and Cu(II) in a chelating κ-N,O bidentate fashion, affording complexes [Zn(HL^1^)_2_(MeOH)_2_] (**1**) and [Cu(HL^1^)_2_] (**3**), respectively (Scheme 2), whereas, the anionic form of (HL^2^) coordinates the Zn(II) and Cu(II) atom in a chelating κ-O,N,N tridentate fashion, affording complexes [Zn(HL^2^)_2_] (**2**) and [Cu(HL^2^)_2_] (**4**) (Scheme 2).

**Figure 2:**
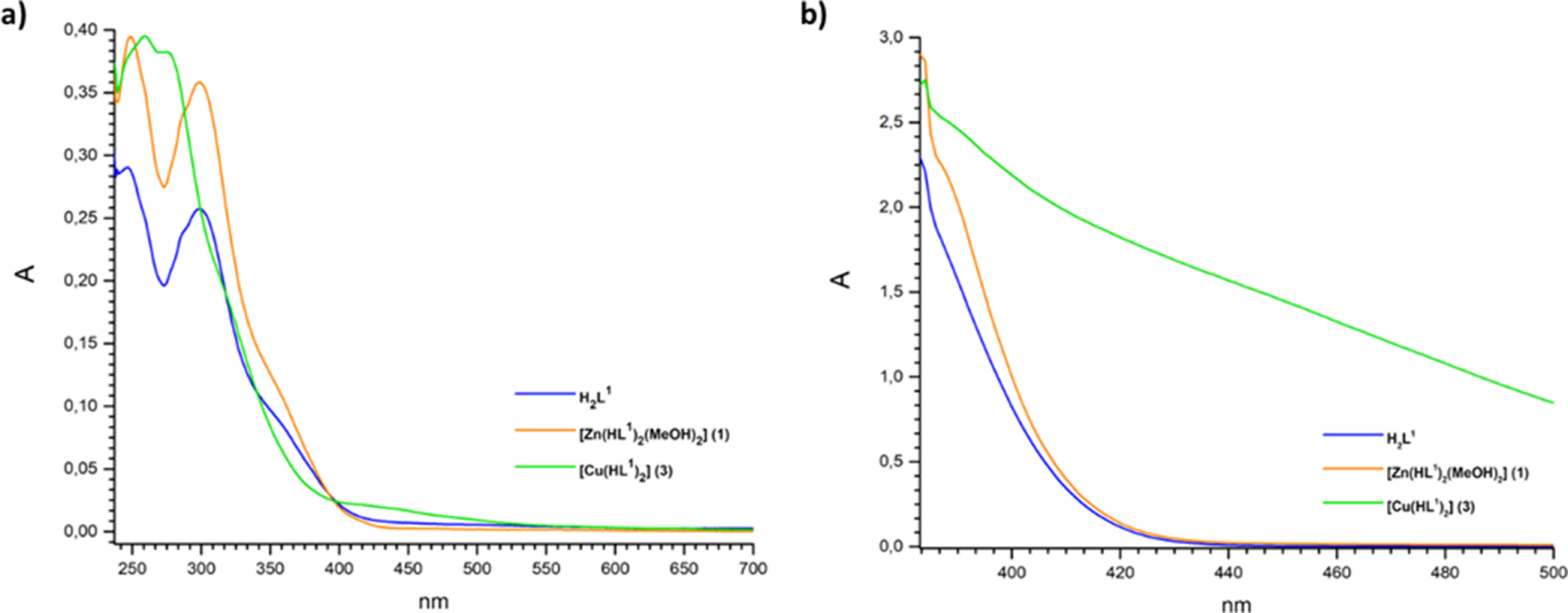
UV–visible spectra of ligand **H**_**2**_**L**^**1**^ and complexes **1** and **3** recorded in CHCl_3_ (a) 10^–5^ and (b) 10^–3^ M.

**Figure 3:**
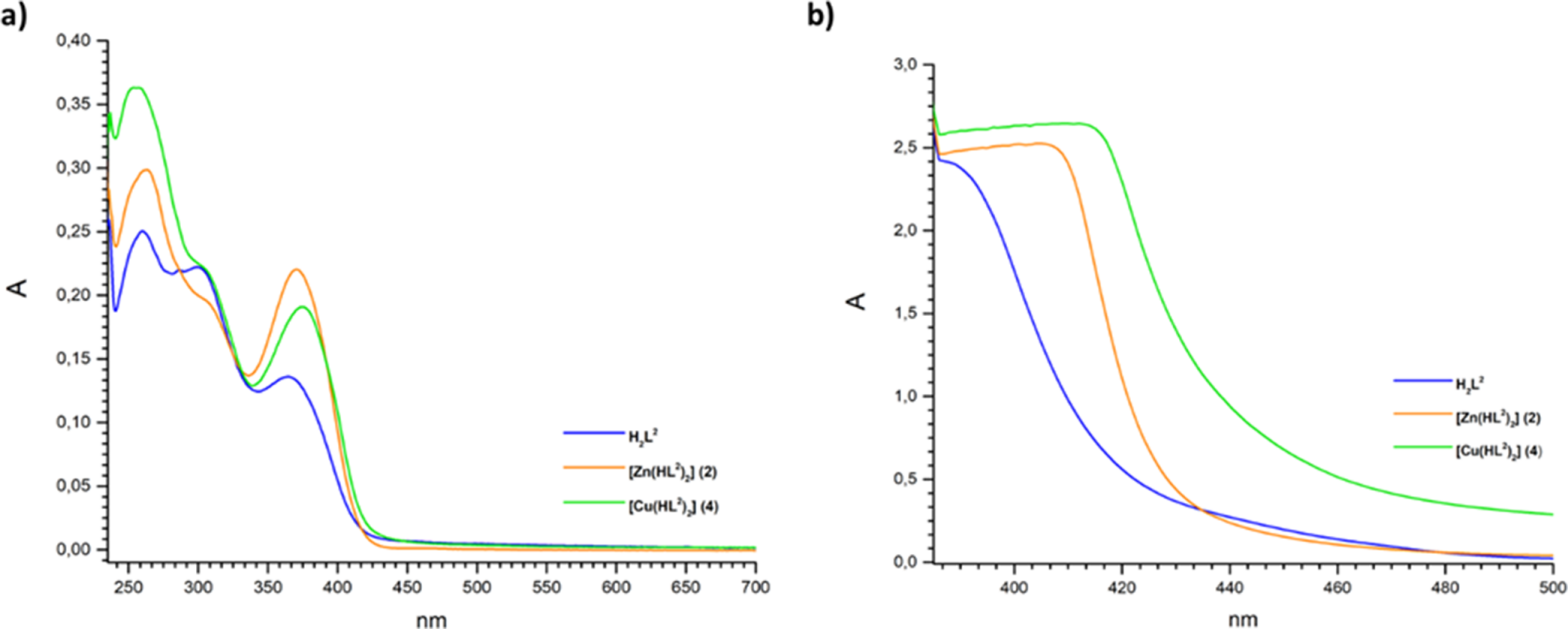
UV–visible spectra of ligand **H**_**2**_**L**^**2**^ and complexes **2** and **4** recorded in CHCl_3_ (a) 10^–5^ and (b) 10^–3^ M.

The structures of **1** and **3**, with the two N,O-chelating ligands in the anti-configuration, have been proposed on the basis of analogous zinc(II) and copper(II) complexes previously reported in the literature with other non-symmetrical pyrazolone-based hydrazones.^21^ Complexes **1–4** are air-stable in the solid state and are soluble in most organic solvents, but not in alcohols and water. The solid-state IR spectra of **1–4** display a shift of ν(C=N) for the azomethine fragment and for HL^2^ also of the pyridine ring, to lower wavelengths with respect to those observed in free proligands, in accordance with coordination to the metal center of the N atom of hydrazide fragments and for **2** and **4**, also N of pyridine. The ν(N–H) stretching produces a sharp band at *ca.* 3300 cm^–1^ in the IR spectra of complexes **1–4** at higher wavelengths than that in free ligands. Additionally, the IR spectrum of **1** displays a broad band at 3134 cm^–1^ due to ν(O–H···N) of extensive intermolecular H-bonding involving coordinated methanol molecules and pyrazole N2 atom of neighboring molecules, which is a structural feature observed in many pyrazolone-based metal complexes.^20,21,27^ In the IR spectra of **1** and **3,** the stretching modes of the CF_3_ group in the chelating HL^1^ were again identified at *ca.* 1320, 1100, and 1065 cm^–1^. In the far-IR region of **1–4,** some medium absorptions in the range of 550–400 cm^–1^ were tentatively assigned to ν(M–N) and ν(M–O).^16,28,29^ The ^1^H and ^13^C NMR spectra in CDCl_3_ of Zn(II) complexes **1** and **2** are in accordance with the expected structures containing the N,O-chelating (**1**) and N,N,O-dichelating (**2**) ligands, confirming the existence of complexes in solution with the ligand signals downfield-shifted with respect to the corresponding signals in the free ligand spectrum. In principle, the geminal methylene protons of the benzyl moiety in proligand **H**_**2**_**L**^**2**^ (H11 in Chart 1) should undergo diastereotopic splitting in complex **2** due to the stereochemistry of the zinc center. In fact, the broad resonance of the CH_2_ group appears as two overlapped signals in the ^1^H NMR spectrum of **2** (see Figure S28). In addition, by recording the ^1^H NMR spectrum of **2** in dimethylsulfoxide (DMSO), the expected AB quartet of the geminal methylene protons is clearly observed (Figure S49). The {^1^H,^15^N}-HSQC and {^1^H,^15^N}-HMBC of **H**_**2**_**L**^**1**^ and complex **1** in chlorinated solvents are quite interesting; in fact, even if the *N*3 resonance at 140.6 ppm in the free **H**_**2**_**L**^**1**^ is not observed for the complex **1**, the *N*4–H resonance shifts from 96.2 to 117.3 ppm and that of *N*2 from 284.9 to 276.4 ppm upon coordination to zinc. Positive ESI-MS spectra recorded in acetonitrile display a similar pattern for all complexes **1–4**, with main peaks being due to [M(HL′)(H_2_L′)]^+^ arising from protonation of one ligand, or [M(HL′)_2_ + Na]^+^, and also to clusters [M_2_(HL′)_3_]^+^. The UV–visible spectra of the ligands and complexes **1–4** were obtained in chloroform solutions at concentrations 10^–3^ and 10^–5^ M, in the range of 200–700 nm at room temperature. The spectra of the **H**_**2**_**L**^**1**^ and **H**_**2**_**L**^**2**^ ligands (Figures 2 and 3) exhibit two bands between 245 and 304 nm due to a ligand-centered π–π* transition localized on the aromatic rings and the n−π* transitions within the >C=N–N chromophore.^30,31^

**Scheme 3:**
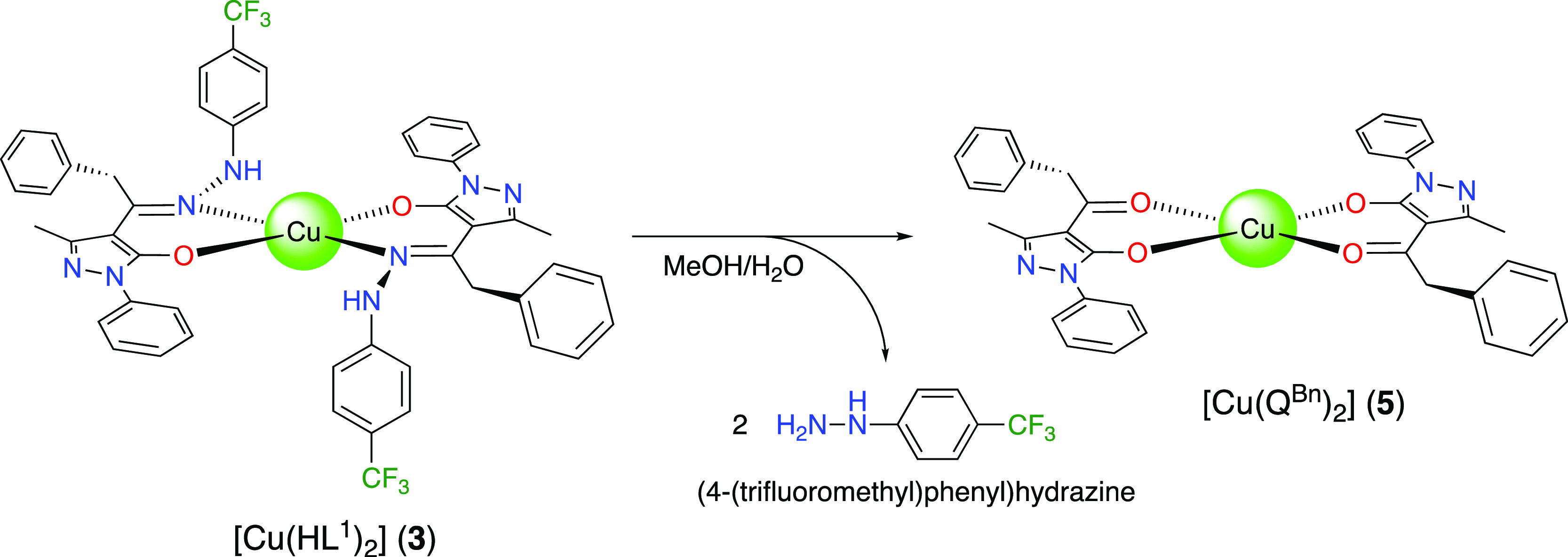
Decomposition Pathway of **3–5**

In addition, the band observed at 370 nm in the **H**_**2**_**L**^**2**^ spectrum can be ascribed to the π → π* transition of pyridine rings present in the ligand structure (Figure 3a).^32^ In the spectra of metal complexes, the bands due to π → π* and n → π* transitions are almost unchanged, apart from Cu(II) complex **3**; however, the band due to the pyridine ring undergoes a bathochromic shift upon coordination in the spectrum of Cu(II) complex **4**. Shoulders around 406–414 nm (Figure 3b) are present due to ligand to metal charge transfer (LMCT) transitions. In the spectra of complexes **1** and **3** (Figure 2a) the bands due to the LMCT transitions appear as shoulders at 388 and 392 nm, respectively. As expected, there are no d–d transitions in complexes **1** and **2** due to the d^10^ configuration of Zn(II); however, also for Cu(II) complexes **3** and **4,** no absorption attributable to d–d transition has been observed, even by increasing the concentration of solutions to 10^–3^ M. When the synthesis of complex **3** is performed at room temperature, leaving the reaction mixture under stirring for 24 h, or under reflux, the red precipitate, corresponding to **3**, slowly converts to a dark green color. This green substance was investigated spectroscopically and with single crystal X-ray analysis which confirmed the degradation of the coordinated HL^1^ to acylpyrazolonate Q^Bn^ ligands, by loss of (4-(trifluoromethyl)phenyl)hydrazine. The new complex is indicated as [Cu(Q^Bn^)_2_] (**5**) (Scheme 3). The IR spectrum of **5** displays strong absorptions at 1604, 1590, and 1574 cm^–1^ due to ν(C=O), ν(C=N), and ν(C=C) and at 510 and 401 cm^–1^, assigned to ν(Cu–O).

## Crystallography

**Figure 4:**
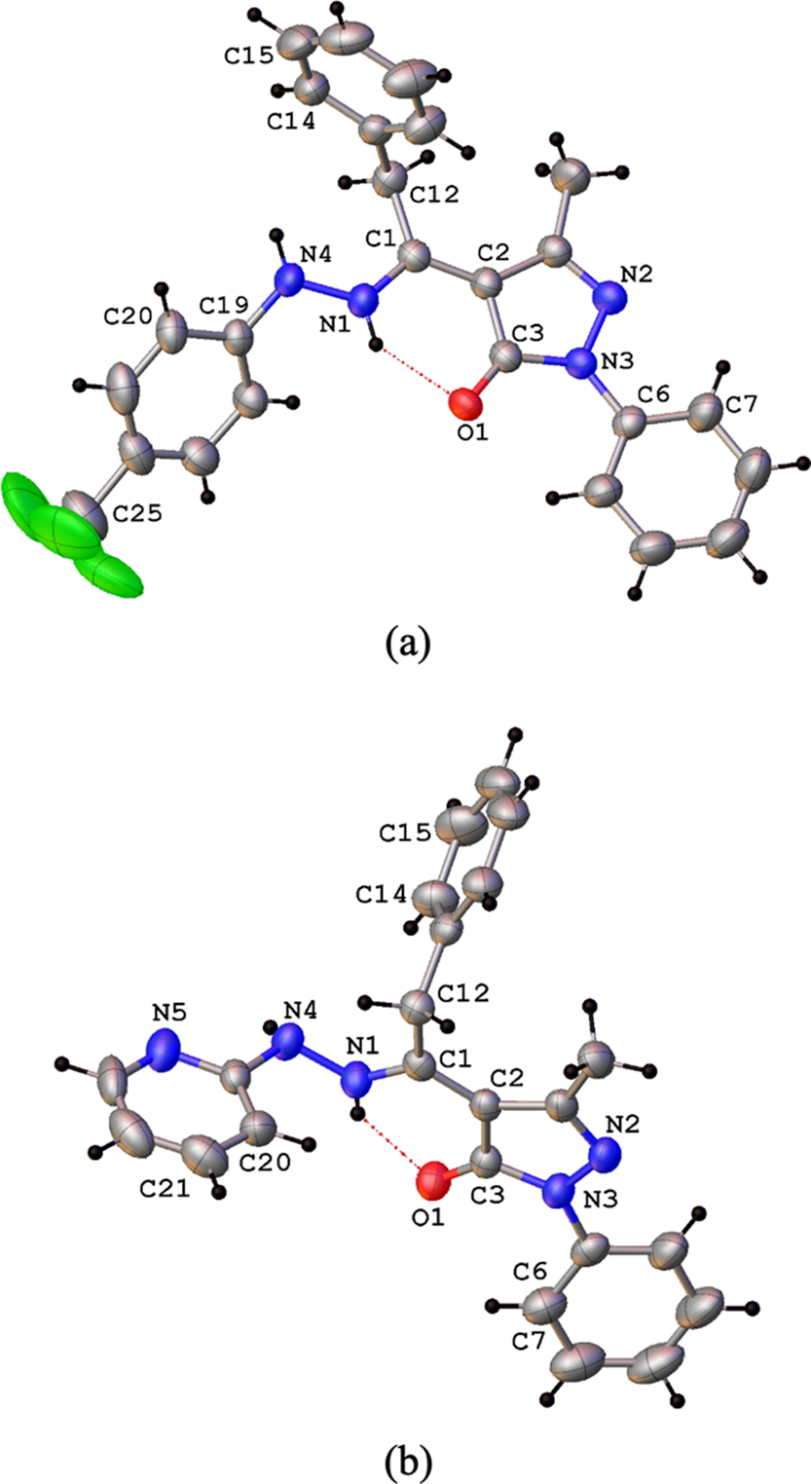
Ortep view of the asymmetric unit content of **H**_**2**_**L**^**1**^ (a) and **H**_**2**_**L**^**2**^ (b) with the atomic numbering scheme and intramolecular N–H···O hydrogen bond (ellipsoids at the 40% level).

**Figure 5:**
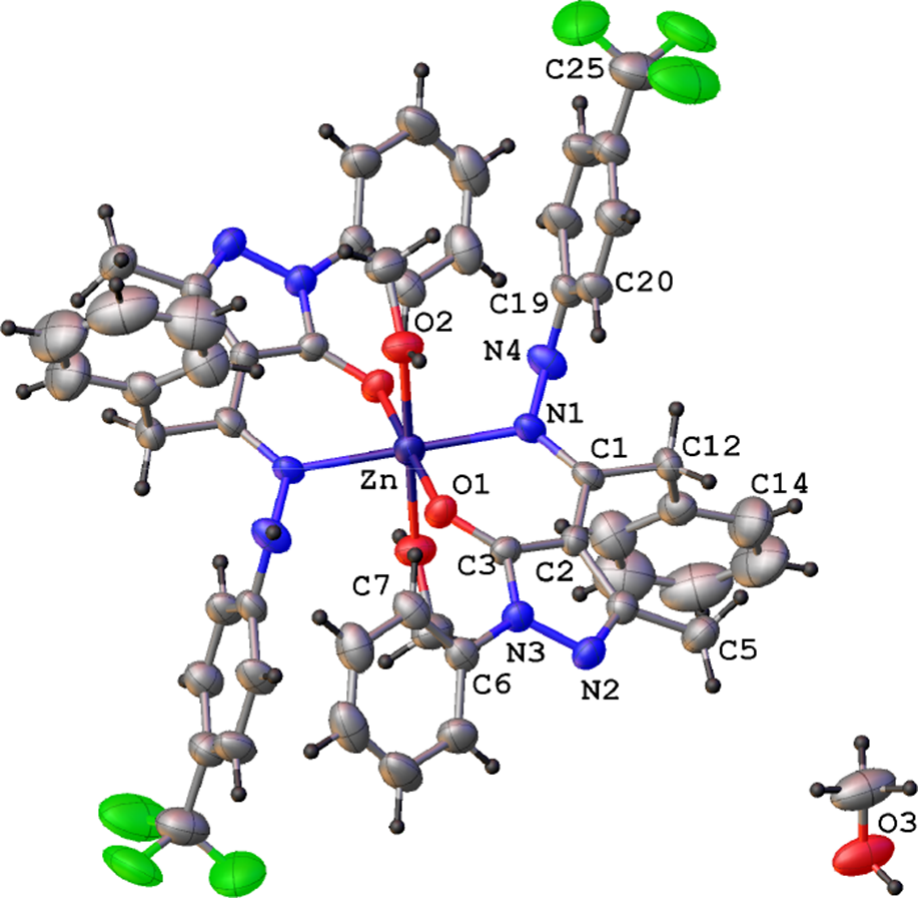
Ortep view of the asymmetric unit content of [Zn(HL^1^)_2_(MeOH)_2_] (**1**) with the atomic numbering scheme (ellipsoids at the 40% level).

The X-ray single crystal molecular structure of proligands **H**_**2**_**L**^**1**^ and **H**_**2**_**L**^**2**^ and of complex **1**, with the atomic numbering scheme are reported in Figures 4a,b and 5. Selected bond distances and angles are reported in Table 1. Details of data and structural refinements are reported in Supporting Information Tables S1 and S2.

**Table 1:** Selected Bond Distances (Å) and Angles (deg) in Proligands **H**_**2**_**L**^**1**^ and **H**_**2**_**L**^**2**^ and Complexes **1** and **5**^a^

ligands	**H**_**2**_**L**^**1**^	**H**_**2**_**L**^**2**^
N(1)–N(4)	1.401(3)	1.400(3)
N(1)–C(1)	1.335(3)	1.318(3)
C(1)–C(2)	1.401(3)	1.400(3)
C(2)–C(3)	1.438(3)	1.436(4)
C(2)–C(4)	1.451(3)	1.435(4)
C(3)–N(3)	1.390(3)	1.371(3)
C(3)–O(1)	1.264(3)	1.256(3)
N(2)–N(3)	1.412(3)	1.399(3)
N(2)–C(4)	1.309(3)	1.304(3)
N(3)–C(6)	1.416(3)	1.418(3)

complexes	**1**	**5**
M–O(1)	2.008(2)	1.893(1)
Zn–N(1)	2.100(3)	
Cu–O(2)		1.931(1)
Zn–O(2)_solv_	2.223(2)	
N(1)–N(4)	1.420(4)	
N(1)–C(1)	1.302(4)	
O(2)–C(1)		1.268(2)
C(1)–C(2)	1.435(4)	1.420(3)
C(2)–C(3)	1.417(4)	1.404(3)
C(2)–C(4)	1.425(4)	1.438(3)
C(3)–N(3)	1.359(4)	
C(3)–O(1)	1.281(4)	1.276(2)
O(1)–Zn–N(1)	87.9(1)	
O(1)–Zn–O(2)	85.7(1)	
O(1)–Zn–N(1)^i^	92.1(1)	
N(1)–Zn–O(2)^i^	89.0(1)	
O(1)–Cu–O(2)		94.0(1)
O(1)–Cu–O(2)^ii^		86.0(1)
O(2)–Cu–O(1)^ii^		86.0(1)

a i = −*x* + 2, −*y*, −*z* + 2; ii = −*x*, −*y* + 2, −*z* + 2.

Both proligands **H**_**2**_**L**^**1**^ and **H**_**2**_**L**^**2**^ are found in the solid crystalline state, in the N–H,N–H,C=O tautomeric form, crystallizing, in the case of **H**_**2**_**L**^**2**^ in the triclinic space group, differently from the monoclinic phase already reported originating from the unexpected zwitterionic form.^14^ Within both ligands, the C(3)–O(1) bond distances of 1.264(3) and 1.253(2) Å, respectively, are similar to the C=O double bond found in similar pyrazolone-based hydrazones in the same tautomeric form.^18^ Confirming to this, the bond distances N(1)–C(1) and N(2)–C(4) are very near to the C=N double bond. The hydrazone N–N bond distances are similar in the two proligands **H**_**2**_**L**^**1**^ and **H**_**2**_**L**^**2**^ (Table 1). In both cases, the overall structure is not planar, with the major distinctive difference related to the orientation of the hydrazone fragment, with a C–N–N–C torsion angle of 149.5(2) and −94.7(3)° in **H**_**2**_**L**^**1**^ and **H**_**2**_**L**^**2**^, respectively. The intramolecular hydrogen bond between the N–H and C=O carbonyl groups are observed [N(1)···O(1) and N(1)–H(1a)∠O(1) of 2.670(3) and 2.703(3) Å, 139 and 137(3)° in **H**_**2**_**L**^**1**^ and **H**_**2**_**L**^**2**^], as typically formed in these types of ligands. In the case of **H**_**2**_**L**^**2**^, the oxygen atom of the C=O group is also involved in intermolecular hydrogen bonding involving the second N atom of the −NH group of benzylhydrazone (N(4)–H(4a)···O(1)). Moreover, π–π interactions between pyridine rings are a further structural feature in the 3D crystal packing of the **H**_**2**_**L**^**2**^ proligand (Figure S40). The X-ray crystal structure analysis of complex **1** confirmed its neutral nature and therefore the general formula [Zn(HL^1^)_2_(MeOH)_2_]. Complex **1** co-crystallized with one methanol molecule in the asymmetric unit (Figure 5).

**Figure 6:**
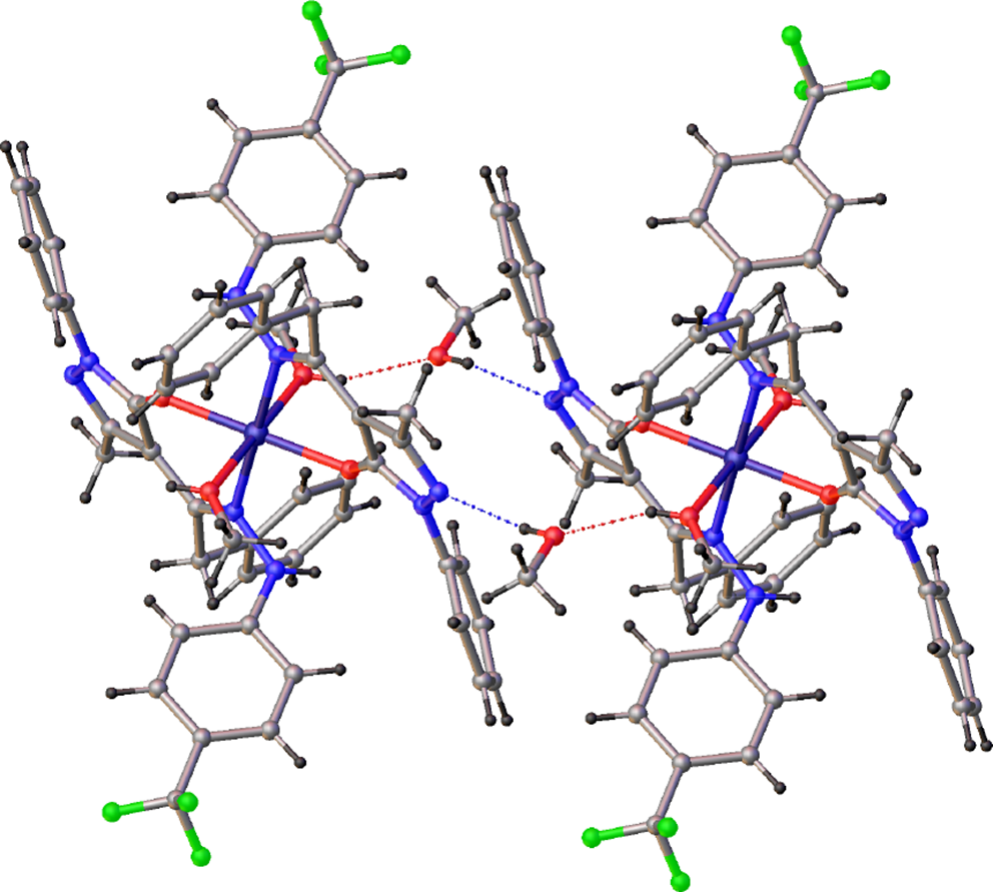
Crystal packing view of **1** showing the N–H···O and O–H···O hydrogen bonds involving both coordinated and lattice methanol molecules.

**Figure 7:**
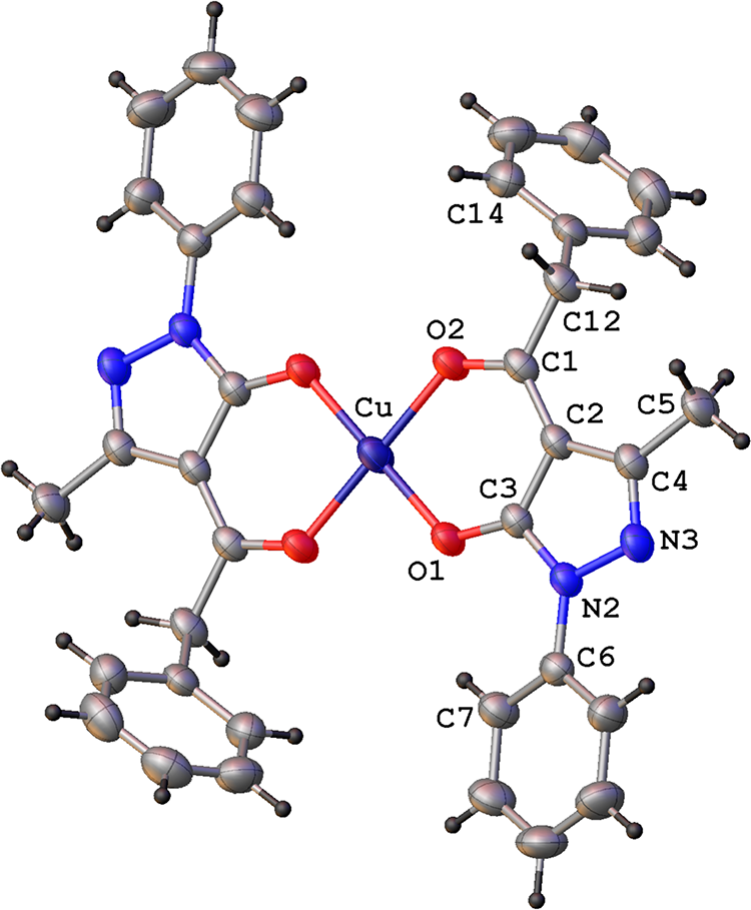
Ortep view of the asymmetric unit content of [Cu(Q^Bn^)_2_] (**5**) with the atomic numbering scheme (ellipsoids at the 40% level).

In complex **1**, the zinc ion, located on the symmetry inversion center, is hexa-coordinated with two N,O bis-chelated **H**_**2**_**L**^**1**^ ligands and two oxygen atoms belonging to methanol molecules. The oxygen and nitrogen atoms of the chelated **H**_**2**_**L**^**1**^ ligands lie in the same plane with bond distances at the zinc ion comparable with those found in analogous complexes. The distances between the zinc ion and the oxygen atoms of the methanol molecules are relatively elongated with respect to the other Zn–O distances (Table 1). Most of the intermolecular interactions are attributable to methanol molecules both coordinated and co-crystallized (Figure 6). In particular, the co-crystallized methanol molecules act as both hydrogen bond donors and acceptors, with the formation of O–H–N and O–H–O interactions with the nitrogen atom N(3) of the pyrazole ring and the hydrogen atom of the coordinated methanol molecule [O(3)···N(2)^i^ 2.828(4) Å, O(3)–H(3)∠N(2) 161°, i = −*x* + 1, −*y* + 1, −*z* + 2; O(2)···O(3)^ii^ 2.698(4) Å, O(2)–H(2)∠O(3) 157°, ii = *x*, *y* – 1, *z*]. The X-ray single crystal molecular structure of complex [Cu(Q^Bn^)_2_] (**5**) confirmed the degradation of the coordinated **H**_**2**_**L**^**1**^ ligands to acylpyrazolones and their O_2_-chelation to the Cu(II) ion. As reported in Figure 7, the four-coordinated Cu(II) ion, sitting on the symmetry inversion center, is found in a distorted square planar geometry, with the two chelated ligands arranged in anti-conformation (Table 1).

As reported in Table 1, bond distances and angles are similar to those reported for analogous four-coordinated Cu(II) complexes containing two bidentate pyrazolonates.^33,34^ The overall planar central metal core is characterized by a dihedral angle between the best mean planes passing through the chelated six-membered and pyrazole rings of 3.7(1)°, while the rotationally free phenyl rings C(6)–C(11) and C(14)–C(18) show dihedral angles with respect to the chelated six-membered ring of 16.1(1) and 71.4(1)°, respectively.

## Theoretical DFT Analysis

**Chart 2:**
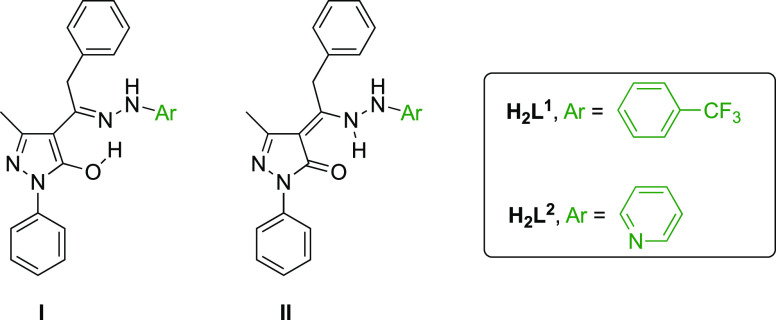
Tautomers I and II of Proligands **H**_**2**_**L**^**1**^ and **H**_**2**_**L**^**2**^

Tautomers I and II of proligands **H**_**2**_**L**^**1**^ and **H**_**2**_**L**^**2**^ (Chart 2) were examined using density functional theory (DFT) at the B3LYP/6-311G** level of theory. In both cases, the energy differences between them are small (*e.g.*, *ca.* 2 kcal/mol for **H**_**2**_**L**^**1**^ in the gas phase; see Table S3 for other data). In solution, tautomer I is the predominant species according to experimental NMR data (see discussion above). This is corroborated by the calculation of the NMR for tautomer I of **H**_**2**_**L**^**1**^. The comparison of the computed ^1^H and ^13^C NMR spectra with the experimental data is good, while a poorer fit for these spectra was found for tautomer II of **H**_**2**_**L**^**1**^ (see Figure S41). However, in the solid state, the observed species is tautomer II. The comparison of selected structural parameters of the tautomer II of **H**_**2**_**L**^**1**^ with those from X-ray data is good (see Figure S42), which confirms the proposed assignment. In particular, the experimental C=O bond distance of 1.264(3) Å is well reproduced by calculations (1.241 Å for II *vs* 1.327 Å for tautomer I). Furthermore, the calculated IR spectrum of tautomer II of **H**_**2**_**L**^**1**^ fits well with the experimental solid-state IR and confirms the assignments previously discussed (see Figure S43 and Table S4).

**Figure 8:**
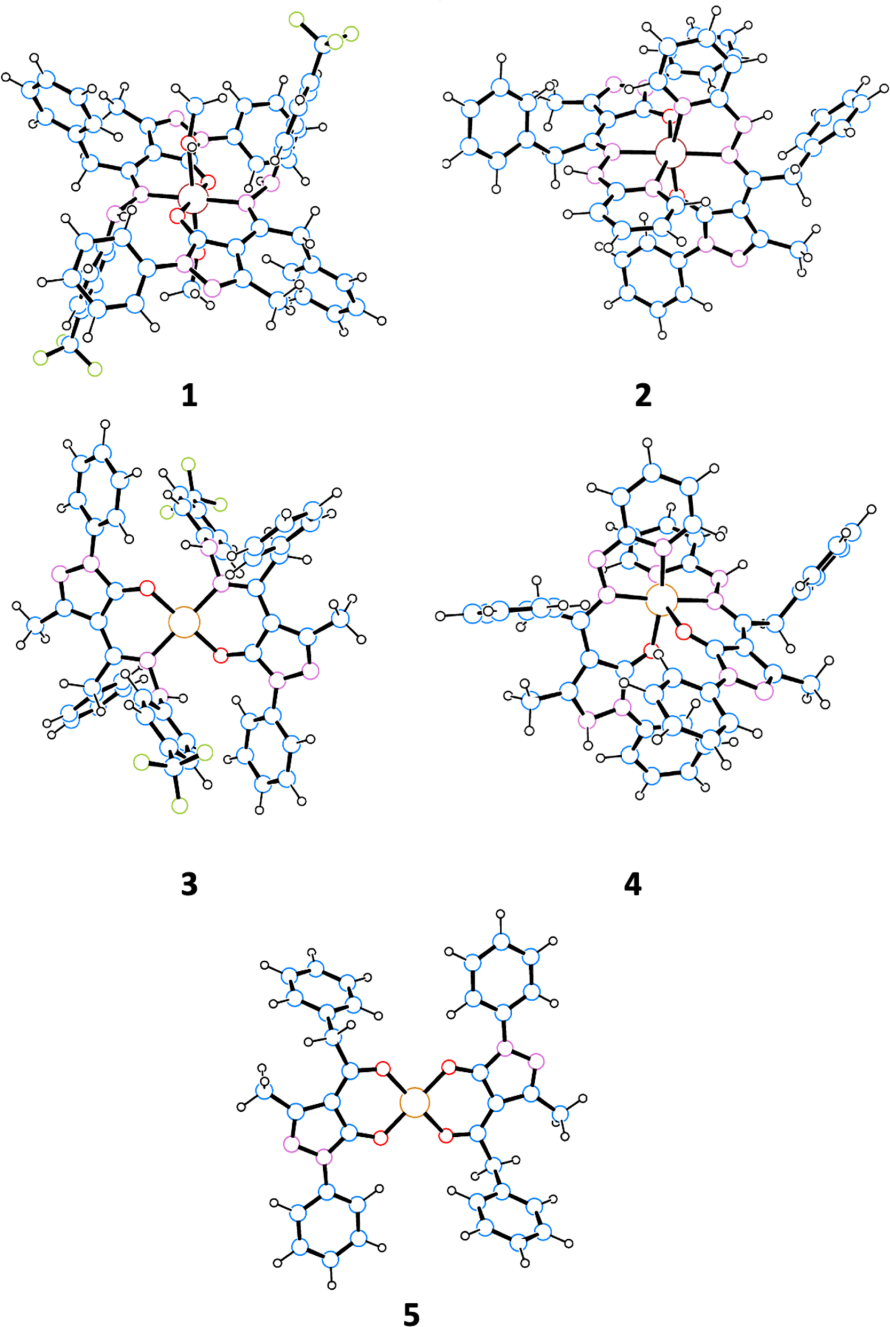
Optimized structures of complexes **1–5**.

To gain information on the coordination capabilities of the ligands in metal complexes **1–4**, the anions [HL^1^]^−^ and [HL^2^]^−^ were also optimized. The bonding localization of [HL^1^]^−^ agrees with the deprotonation of tautomer II, showing a shorter C=O bond (1.232 Å) than the C–N bond (1.303 Å). Evidently, these distances change upon coordination, as we will discuss later. MOs involved in the coordination to the metal atom for the HL^1^ ligand are HOMO – 2 and HOMO – 3, with a minor contribution of HOMO – 9 (Figure S44), and these are MOs in which the lone pairs of the N and O donor atoms take part in the in-phase and out-of-phase contributions of the σ type, which afford the M–O and M–N bonds. These MOs were compared with those obtained from the single-point calculation of the HL^1^ ligand with the geometry found in the optimization of complex **1**. In this case, the in-phase and out-of-phase combinations are clearly detected in HOMO – 1 and HOMO – 3 (also shown in Figure S44 for an appropriate comparison). Concerning the [HL^2^]^−^ anion, its structure fits again well with the deprotonation of tautomer II with a shorter C=O bond (1.242 Å) than the C–N bond (1.296 Å). The anion does not show the planar conformation expected in complexes **2** and **4,** and for this reason, we analyzed their MOs from the single-point calculation of the HL^2^ ligand with the approximately planar geometry found in the optimization of complex **2**. The MOs involved in the coordination to the metal are HOMO – 1, HOMO – 2, HOMO – 3, HOMO – 4, and HOMO – 6 (Figure S44). The lone pair of the oxygen atoms is distributed between HOMO – 1 and HOMO – 2 and the out-of-phase combinations of both N lone pairs appear at HOMO – 3 and HOMO – 4, while HOMO – 6 is clearly the in-phase combination of both N lone pairs. Complexes **1–5** were also analyzed by DFT. The resulting optimized structures of these complexes are shown in Figure 8.

The selected combination of the method and basis sets provides a good structural description of these complexes according to the good comparison of the calculated and experimental structural parameters of complex **1** (Table S5). The HL^1^ ligands exhibit delocalized C–O and C–N bonds (1.279 and 1.315 Å, respectively), in agreement with experimental values, and the two six-membered metallacycles [Zn(HL^1^)] show an envelope conformation with an angle between the ligand and molecular planes of 30.3° (experimental 30.9°). The proposed optimized structure of **1** also gives a calculated NMR that matches well with experimental ^1^H and ^13^C NMR spectra (*R*^2^ value of 0.9971 in the correlation shown in Figure S45). To rationalize the observed trans disposition of methanol molecules in **1**, we optimized the hypothetic complex [Zn(HL^1^)_2_] without these solvent ligands. The resulting structure is square planar (Figure S46), which is unexpected for a four-coordinated d^10^ complex. This fact suggests that the adoption of such a geometry is mainly due to steric reasons. This is confirmed by optimizing the related complex [Zn(HL^3^)_2_], where the HL^3^ ligand is like HL^1^, but the *p*-trifluoromethylphenyl substituent is replaced by a methyl group. Optimized [Zn(HL^3^)_2_] is tetrahedral and is 5.8 kcal/mol (Δ*G*) more stable than the square planar structure (Figure S46). Consequently, the presence of the *p*-trifluoromethylphenyl group in HL^1^ causes enough steric pressure to impede the adoption of the expected d^10^-tetrahedral arrangement of [Zn(HL^1^)_2_], explaining the *trans*-(MeOH)_2_ geometrical configuration observed in **1**. A square planar structure closely related to that found for [Zn(HL^1^)_2_] was optimized for complex [Cu(HL^1^)_2_], **3**. The six-membered metallacycles within the [M(HL^1^)] moiety display an envelope conformation in both cases, and the angle between the ligand and molecular planes is quite similar in both complexes (29.5 and 28.8° for Cu and Zn, respectively). A minor difference found is the M–O and M–N bond distances which are slightly longer for zinc (2.029 and 2.013 Å, respectively) than those for copper (1.950 and 1.987 Å, respectively), in agreement with its higher ionic radius.^35^ Complexes **2** and **4** have an analogous formulation, [M(HL^2^)_2_], but their optimized structures showed some differences. In **4,** the Jahn–Teller effect with two distances is clearly appreciable, Cu–O and Cu–Npy of 2.204 and 2.399 Å, respectively, which are longer than other Cu–O and Cu–N_py_ (2.045 and 2.000 Å, respectively) and longer than those observed for Zn complex **2** (Zn–O: 2.045 and 2.053 Å; Zn–N_py_: 2.183 and 2.191 Å). Furthermore, the planes defined for the HL^2^ ligand formed different angles in both complexes. This angle between the two planes is 83.9° for the Zn derivative, close to the regular angle of 90° for an ideal octahedral geometry, while for the Cu complex, it is more distorted with respect to such a geometry, 72.9°. As occurred with complex **1**, the proposed calculated structure of **2** provides an excellent NMR prediction for this complex, according to the correlation of experimental and calculated ^1^H and ^13^C NMR parameters (*R*^2^ value of 0.9991 in the correlation shown in Figure S45). Complex **5** was also optimized and shows a square planar structure, which is typical for related four-coordinated copper–acylpyrazolonate complexes.^20^ Again, a good comparison of the calculated and experimental structural parameters of this complex was found (Table S6), which is common for these [Cu(Q^R^)_2_] complexes.^36^

## Cytotoxicity Studies

Both the free ligands **H**_**2**_**L**^**1**^ and **H**_**2**_**L**^**2**^ and the metal complexes **1–4** were assayed *in vitro* on *T. brucei* and Balb/3T3 cells (mammalian cells), and interesting results were obtained (Table 2). EC_50_ values were calculated with the GraphPad Prism 5.2 software and the selectivity index by the correlation of the obtained values for *T. brucei* versus the mammalian reference cells. First of all, **H**_**2**_**L**^**2**^ was slightly more active against *T. brucei* than **H**_**2**_**L**^**1**^ (EC_50_ = 0.189 *vs* 0.213 μM), but also more cytotoxic, having an EC_50_ value of 0.465 μM against mammalian cells, compared to 12.51 μM for **H**_**2**_**L**^**1**^. From the SI, it is clear that the safety profile of **H**_**2**_**L**^**1**^ is much better than the **H**_**2**_**L**^**2**^ profile, and **H**_**2**_**L**^**2**^ seems to strongly affect both parasitic and mammalian cells.

**Table 2:** Activity of Tested Compounds on *T. bruceiTC221* and Balb/3T3 Cells

compound	EC_50_*T. brucei* TC221	(μM) Balb/3T3	SI
**H**_**2**_**L**^**1**^	0.231 ± 0.008	12.51 ± 1.354	55
**H**_**2**_**L**^**2**^	0.189 ± 0.038	0.465 ± 0.013	2.460
1	0.084 ± 0.005	12.322 ± 0.346	>100
2	0.169 ± 0.038	0.349 ± 0.109	2.065
3	4.347 ± 2.610	32.977 ± 0.347	7.586
4	12.763 ± 4.060	17.163 ± 3.448	1.345
suramin	0.023 ± 0.0008	43.912 ± 1.438	>100

As evident from Table 2, also Zn and Cu complexes of **H**_**2**_**L**^**1**^ (**1** and **3**) are less toxic than Zn and Cu complexes (**2** and **4**) of **H**_**2**_**L**^**2**^. Therefore, the selectivity index is very low for **2** and **4** (SI = 2.065 and 1.345, respectively) and much higher for **1** and **3** (SI > 100 and 7.586, respectively). This is in line with the results above which **H**_**2**_**L**^**1**^ is always much more specific than **H**_**2**_**L**^**2**^, regardless of whether it is complex or not. The interesting results obtained for **H**_**2**_**L**^**1**^ and its complexes **1** and **3** pushed the research to focus on these compounds. In particular, **1** seems to be more active than **H**_**2**_**L**^**1**^, and it also maintains a good safety level since SI is > 100. Because of the derivation of complex **1** from **H**_**2**_**L**^**1**^, it is logical to suppose that the type of action of these two compounds against *T. brucei* could be the same. Among the complexes tested, **1** is the most convincing; for this reason, we investigated the hypothetical mechanism of action of **1** and its precursor **H**_**2**_**L**^**1**^ by measuring the cellular nucleotide pools, which gives information of nucleotide-metabolizing enzymes and energy metabolism and redox status (NADPH is required by ribonucleotide reductase).

## Mechanism of Action

**Figure 9:**
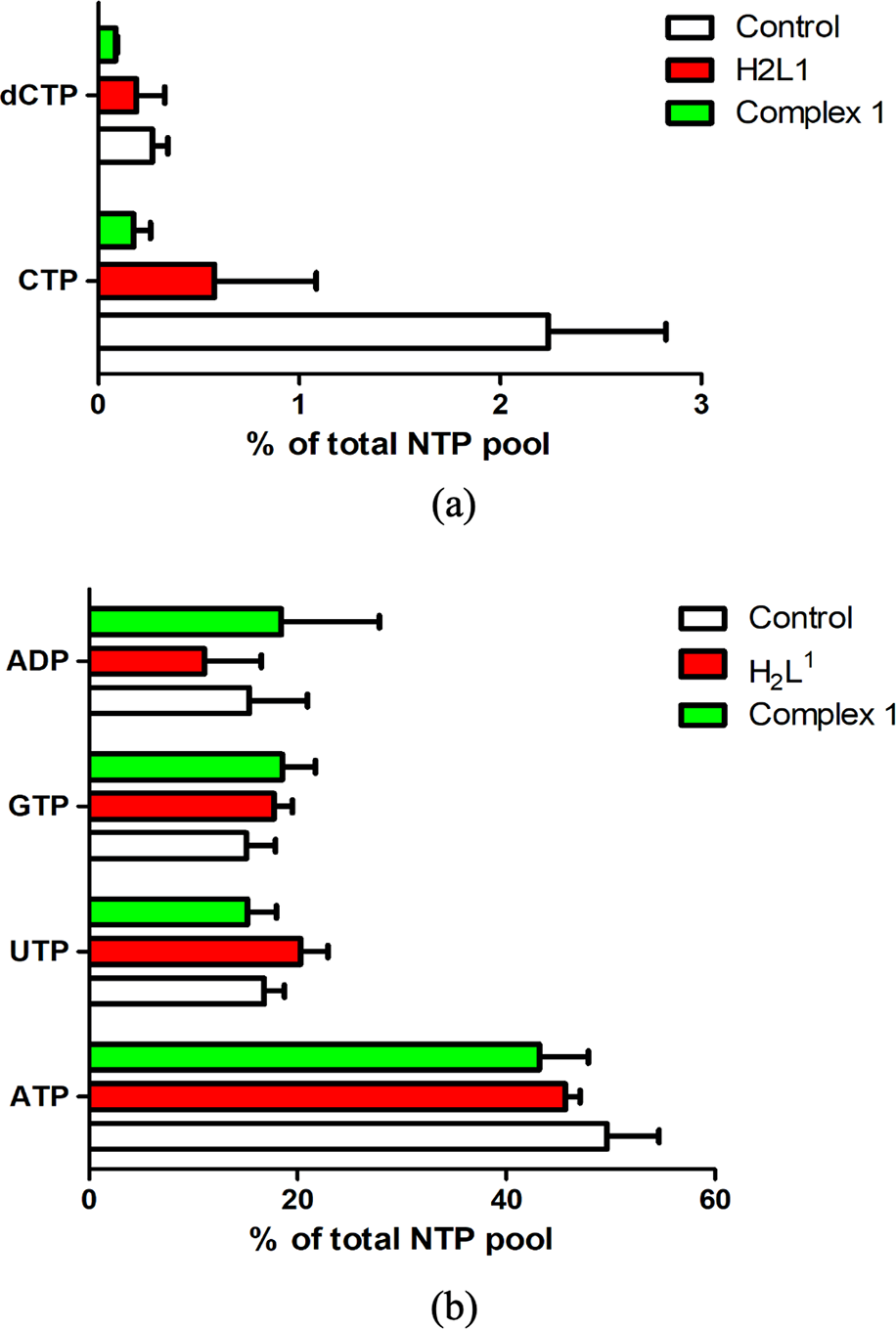
(a) Differences in CTP and dCTP pools in non-treated cells and cells treated with **H**_**2**_**L**^**1**^ and **1** (both of them at a concentration of 5 μM for 1 h). (b) Differences in NTP pools in non-treated cells and cells treated with **H**_**2**_**L**^**1**^ and **1** (both of them at a concentration of 5 μM for 1 h).

Untreated *T. brucei* NTP, dNTP, and ADP pools were measured by HPLC and compared to the pools of parasites treated with **H**_**2**_**L**^**1**^ and complex **1**. For the analysis, the method by Ranjbarian *et al.* was applied.^37^ The procedure started by adding 5 μM of the drug tested to 50 mL of a *T. brucei* culture (10^6^ logarithmically growing cells per mL) and incubating the parasites for 1 h before extracting the NTPs, dNTPs, NDPs, and dNDPs from them with trichloroacetic acid and quantifying the nucleotides with HPLC. From these experiments, it was possible to evaluate changes in nucleotide pools between non-treated trypanosomes and those treated with **H**_**2**_**L**^**1**^ and **1** (chromatographs in Figure S47). A striking difference between the drug-treated and non-treated cells was the levels of CTP and dCTP. In fact, although CTP and dCTP are low also in *T. brucei* cells without treatment (∼2% of the total NTP pool), they are much lower in parasites treated with **H**_**2**_**L**^**1**^ and especially with the Zn-complex **1** (Figure 9a). The CTP pools were decreased to 25% in the cells treaded with **H**_**2**_**L**^**1**^ and 6% in the cells treated with **1,** as compared to the control cells. This result is in accordance with the results obtained in Table 2; complex **1** is more active than **H**_**2**_**L**^**1**^ (EC_50_ value is lower), and it is therefore logical that if CTP is affected by the treatment with **H**_**2**_**L**^**1**^, this effect is much stronger with complex **1**. No obvious effect was observed on the other NTPs or ADP (Figure 9b). From these results, it can be assumed that the target of these compounds could be CTPS.

**Figure 10:**
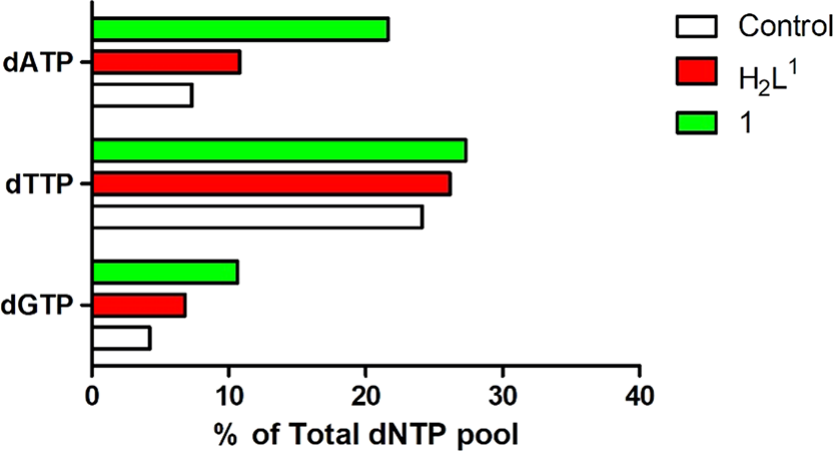
Increased deoxynucleotide pools in cells treated with 5 μM **H**_**2**_**L**^**1**^ and **1** for 1 h (compared to deoxynucleotide pools in non-treated control cells).

Indeed, CTPS is the enzyme responsible for the *de novo* synthesis of CTP from UTP, which is the only pathway of synthesis of CTP in *T. brucei*.^19^ The low CTP level could also be a reason why less dCTP is produced. CTP and other NTPs in the cell are in equilibrium with the corresponding diphosphates. Consequently, it will be less CDP substrate for ribonucleotide reductase to make dCDP from dATP, dGTP, and dTTP levels increased in trypanosomes treated with **H**_**2**_**L**^**1**^ and even more in trypanosomes treated with **1** (Figure 10). This could be a consequence of the low dCTP pools, leading to that the other deoxynucleotides accumulate in the cell as the DNA polymerase is not able to synthesize DNA because of the lack of dCTP. The inhibition of DNA synthesis leads to growth inhibition.

To further investigate the different activities between Zn(II) complexes **1** and **2**, their stability in DMSO-*d*_6_ was evaluated through an NMR study within 48 h. While the ^1^H NMR of complex **2** remains unchanged within 48 h (Figure S49), confirming its stability in DMSO, in the case of complex **1,** some modifications have been immediately observed after dissolution. In fact, the appearance of a resonance due to free MeOH at 3.16 ppm confirms its replacement in the zinc coordination environment by deuterated-DMSO molecules. Moreover, the geminal CH_2_ of the benzyl group in (HL^1^) affords three different signals, two of them undergoing diastereotopic splitting, in accordance with the presence of a trans and two cis isomers, containing a chiral zinc center, in equilibrium with each other (Figure S48). Furthermore, by comparison with the spectrum of the free **H**_**2**_**L**^**1**^ ligand in DMSO-*d*_6_, no ligand release was observed. In conclusion, complex **1** in DMSO replaces methanol with DMSO molecules in the zinc environment and undergoes trans–cis interconversion without any decomposition.

## Conclusions

In conclusion, we have reported two novel pyrazolone-based hydrazones **H**_**2**_**L**^**1**^ and **H**_**2**_**L**^**2**^ and their Zn(II) and Cu(II) complexes **1–4**. The compounds were fully characterized both in the solid state and solution, together with a bis(acylpyrazolonate)copper(II) species (**5**) arising from decomposition of the complex [Cu(HL^1^)_2_] (**3**). All ligands and metal complexes were structurally characterized both in the solid state and solution and investigated as potential antitrypanosomal agents, and the overall results of this study shed light on the biological properties of this new series of compounds as a relevant source of bioactive substances, which can serve as possible lead candidates for further antiprotozoal drug development. The exhibited antitrypanosomal activity of **H**_**2**_**L**^**1**^ and its Zn(II) complex **1,** revealed through biological *in vitro* assays, is an important result also because a very low cytotoxicity has been detected. An important outcome is the finding of the mechanism of action. The analysis of NTP and dNTP pools clearly revealed that CTP is severely affected by **H**_**2**_**L**^**1**^ and its Zn(II) complex **1**. *T. brucei* lacks salvage pathways for CTP synthesis, and our preliminary results on the purified *T. brucei* CTPS indicate that it is the targeted enzyme of the Zn(II) complex **1**, whereas **H**_**2**_**L**^**1**^ seems inactive by itself. The effect of **H**_**2**_**L**^**1**^ on the CTP/dCTP pools may possibly come from that it is chelated by naturally occurring metals in the trypanosomes or the growth medium. Accordingly, the Zn(II)-chelated complex **1** had a stronger effect on the CTP and dCTP pools than **H**_**2**_**L**^**1**^. CTPS was previously found to be a good target in *T. brucei* because in contrast to the situation in mammalian cells, the inhibition of this enzyme cannot be rescued by cytidine (or cytosine) in the surrounding medium. The parasite was therefore sensitive to the CTPS inhibitors 6-diazo-5-oxo-l-norleucine (DON) and α-amino-3-chloro-4,5-dihydro-5-isoxazoleacetic acid (acivicin).^19^ However, DON and acivicin are both glutamine analogues, and in mammalian cells, they also affect other glutamine-requiring enzymes/pathways such as *de novo* purine biosynthesis (DON) and GMP synthase (acivicin). Indeed, they were originally tried out as anticancer drugs, and the selectivity against the trypanosomes is dependent on nucleosides/nucleobases in the growth medium that selectively rescues the mammalian cells. In contrast, the drugs investigated here are from another class of compounds and have a high selectivity against the trypanosomes with almost no effect on the mammalian reference cells. Since then, improved versions of acivicin have been produced with higher selectivity against *T. brucei* CTPS.^38^ Experience from studies on mammalian cells has shown that it is possible to achieve much higher selectivity against CTPS over other cellular enzymes by using cytidine/uridine analogues such as cyclopentenyl cytosine. However, these analogues need to be phosphorylated in the cell to achieve their function as CTPS inhibitors, and *T. brucei* lacks uridine–cytidine kinase. All drugs developed against the *T. brucei* CTPS have therefore so far been glutamine analogues. In contrast, the drugs investigated here are from another class of compounds. Furthermore, they have a high selectivity against the trypanosomes with almost no effect on the mammalian reference cells.

## Experimental Section

### Materials and Methods

All reagents and solvents were purchased from Sigma-Aldrich Chemical Co and were of analytical grade and used as received. Thin-layer chromatography (TLC) was run on silica gel 60 F254 plates. The final compounds were characterized by ^1^H NMR, ^19^F NMR, ^13^C NMR, MS, and elemental analyses. ^1^H NMR and ^13^C NMR spectra were recorded with the 500 Bruker Ascend (500 MHz for ^1^H, 470.6 for ^19^F, and 125 MHz for ^13^C) instrument operating at room temperature. The chemical shift values are expressed in δ values (ppm), and coupling constants (*J*) are in Hertz; tetramethylsilane (TMS) was used as an internal standard. Proton chemical data are reported as follows: chemical shift, multiplicity (s = singlet, d = doublet, dd = doublet of doublets, t = triplet, dt = doublet of triplets, q = quartet, dq = doublet of quartets, and m = multiplet, brs = broad singlet) coupling constant(s), and integration. The presence of all exchangeable protons was confirmed by addition of D_2_O. ^1^H NMR and ^13^C NMR spectra were assigned with the aid of {^1^H–^1^H} COSY, {^1^H–^13^C} HSQC, and {^1^H–^13^C} HMBC NMR techniques. Indirect ^15^N NMR chemical shifts were assigned based on the {^1^H–^15^N}-HSQC and {^1^H–^15^N}-HMBC NMR techniques. Mass spectra were recorded on an HP 1100 series instrument. All measurements were performed in the positive ion mode using atmospheric pressure electrospray ionization (API-ESI). Elemental analyses (C, H, and N) were determined on the Thermo Fisher Scientific FLASH 2000 CHNS analyzer and are within 0.4% of theoretical values. Melting points are uncorrected and were recorded on the STMP3 Stuart scientific instrument and on a capillary apparatus.

### X-ray Crystallography

Single-crystal X-ray diffraction data of proligands **H**_**2**_**L**^**1**^ and **H**_**2**_**L**^**2**^ and complexes **1** and **5** were collected at room temperature with the Bruker-Nonius X8APEXII CCD area detector system equipped with a graphite monochromator with radiation Mo Kα (λ = 0.71073 Å). Data were processed through the SAINT reduction and SADABS absorption software.^39,40^ Structures were solved by direct methods and refined by full-matrix least-squares based on *F*^2^ through the SHELX and SHELXTL structure determination package.^40^ All non-hydrogen atoms were refined anisotropically. Fluorine atoms F(2) and F(3) of the −CF_3_ group in complex **1** are found to be disordered in two positions and refined with an occupancy factor of 0.80 and 0.20, respectively. Both sets of atoms were refined anisotropically. Hydrogen atoms were included as idealized atoms riding on the respective carbon, nitrogen, and oxygen atoms with bond lengths appropriate to the hybridization. H(1a) and H(4a) hydrogen atoms in **H**_**2**_**L**^**2**^ were located in the best difference map and refined isotropically. Constrains and restrains have been applied on the N5/C(23) pyridine ring (refinement with idealized geometry and thermal motion restrains) in order to fix a slight disorder. All graphical representations have been obtained by using the Olex2 software package.^41^ Details of data and structural refinements are reported in Supporting Information Tables S1 and S2. CCDC 2174178–2174181 contains the supplementary crystallographic data for this article.

### Computational Details

The electronic structure and geometries of the proligands **H**_**2**_**L**^**1**^ and **H**_**2**_**L**^**2**^, their tautomers and anions, [HL^1^]^−^ and [HL^2^]^−^, and zinc and copper complexes were investigated by using DFT at the B3LYP level.^42,43^ For the proligands and their corresponding anions, the 6-311G** basis set was used for the optimization, while for the Zn and Cu complexes, the optimization was carried out using 6-311G*. Molecular geometries were optimized without symmetry restrictions. Frequency calculations were carried out at the same level of theory to identify all of the stationary points as minima (zero imaginary frequencies) and to provide the thermal correction to free energies at 298.15 K and 1 atm. Solution-phase SCF energies were calculated by a single-point calculation on the in vacuum optimized structure using the CPCM solvation model in chloroform.^44^ Gibbs free energies in chloroform solution were estimated from the equation *G*_solv_ = *E*_solv_ + (*G*_gas_– *E*_gas_). The GIAO method was used for the NMR calculations (^1^H-, ^13^C-, and ^15^N NMR isotropic shielding tensors) which were carried out at the 6-311++G** level of theory. The computed IR spectra were scaled by a factor of 0.96.^45,46^ The DFT calculations were executed using the Gaussian 09 program package.^47^ Coordinates of all optimized compounds are collected in the Supporting Information (Table S7).

### Cell Culture and Cytotoxicity Determinations

The biological assays have been conducted in collaboration with Prof. Anders Hofer, Department of Medical Biochemistry and Biophysics, Umeå University, Umeå, Sweden. *T. bruceiTC221* bloodstream forms and mouse embryonic fibroblast Balb/3T3 cells (ATCC no CCL-163) were cultivated in a vented plastic flask at 37 °C with 5% CO_2_. For *T. brucei*, the growth medium was Hirumi’s modified Iscoves medium (HMI)-9 supplemented with 10% (v/v) fetal bovine serum (Thermo Fischer Scientific Gibco, Waltham, MA, USA), whereas the Balb/3T3 cells were grown in Dulbecco’s modified Eagle’s medium (Sigma-Aldrich) supplemented with 10% (v/v) heat-inactivated fetal bovine serum, glutamine (0.584 g/L), and 10 mL/L 100 × penicillin–streptomycin (Gibco).^48−50^ The synthetic compounds tested were dissolved in DMSO and serially diluted with growth medium in white 96-well microtiter plates. 20,000 bloodstream forms of *T. brucei* cells were added to each well in the final volume of 200 μL. In the case of mammalian cells (Balb3T3), we added 2000 cells/well with similar results. To avoid any damage to the cells, the concentration of DMSO in the solution was never higher than 1% (no cell growth inhibition was observed with this concentration of DMSO). Cell viability was verified by a drug-free control for each compound. The plates were incubated for 48 h in the 5%CO_2_ incubator; then, 20 μL of 0.5 mM resazurine (Sigma-Aldrich) was added to each well, and the plates were incubated for an additional 24 h before the fluorescence was measured with the Synergy H4 microplate reader (excitation wavelength 530 or 540 nm and emission wavelength 590 nm).

The half-maximal efficacious concentration (EC_50_) values were calculated on the log inhibitor versus the response curves by non-linear regression using the GraphPad prism 5.2 software (GraphPad Software, Inc., La Jolla, CA, USA). The procedure was repeated three times to make data reliable.

### Determination of NTP and dNTP Pools by HPLC

The bloodstream form of *T. brucei* (strain 221) was maintained at 37 °C and 5% CO_2_ in Hirumi’s modified Iscove’s medium (HMI)-9 medium and Serum Plus but containing 10% fetal bovine serum. Trypanosomes (50 mL), harvested in the late logarithmic phase, were chilled on ice for 5 min before being collected and centrifuged at 4000 rpm for 5 min at 4 °C. Subsequently, the pellet was resuspended in 1 mL of culture medium, transferred to an Eppendorf tube, and centrifuged at 14000 rpm for 1 min at 4 °C. The NTP, NDP, dNDP, and dNTP pools were not affected by the time on ice (5 min), the centrifugation time (varied between 5 and 15 min), the centrifugation speed (varied between 4000 and 14000 rpm), or how many washings in culture medium were made (3 times). After the medium wash, the collected trypanosomes were disintegrated by pipetting them up and down in 720 μL of ice-cold 0.6 M trichloroacetic acid containing 15 mM MgCl_2_. The resultant solution was centrifuged at 14,000 rpm for 1 min at 4 °C, and the supernatant was extracted twice with 1.13 the volume of Freon (78% v/v)-trioctylamine (22% v/v) or chloroform–trioctylamine. 400 μL of the resulting solution was transferred and centrifuged in a pre-washed Eppendorf tube with a 5 kDa filter (Nanosep 3k Omega, Pall Life Sciences). The sample was purified on a WAX cartridge properly prewashed, and the collected solution was evaporated to dryness in a Speedvac (Savant) and dissolved in 200 μL of water. This fraction was used for quantification of nucleotides and deoxynucleotide diphosphates and triphosphates by a 150 × 3 mm C18-WP HPLC column (Chromanik Sunshell).^37^ The analyses were performed on a 150 × 2.1 mm Sunshell C18-WP 2.6 μm column (ChromaNik Technologies Inc), at 30 °C using a mobile phase of 43% solution A and 57% solution B. Solution A contained 5.8% (v/v) acetonitrile, 23 g/L KH_2_PO_4_, and 0.7 g/L tetrabutylammonium bromide (TBA-Br) adjusted to pH 5.6 with KOH, while solution B contained 5.8% (v/v) acetonitrile and 0.7 g/L TBA-Br. All reagents and solvents were of HPLC grade. The size of the sample loop was 100 μL, and the flow rate was 1.2 mL/min. The peaks were detected by their absorption at 270 nm with a UV-2075 Plus detector. Nucleotides were quantified by measuring peak heights and areas and comparing them to a standard curve.

### Synthesis of the Proligands HL′

#### General Procedure for the Synthesis of **H**_**2**_**L**^**1**^ and **H**_**2**_**L**^**2**^

The acylpyrazolone ligand 5-hydroxy-3-methyl-1-phenyl-1*H*-pyrazol-4-yl)(phenyl)methanone (HQ^Bn^) was synthetized following the method previously reported.^20^ A mixture of HQ^Bn^ (1.0 equiv) and the appropriate hydrazine (1.0 equiv) in methanol (10 mL) containing 5–10 drops glacial acetic acid was heated to 80 °C, and the reaction was monitored by TLC (CH_3_Cl/MeOH 96:4 v/v). A precipitate slowly formed from the hot solution, and after completion, the reaction mixture was placed at 4 °C overnight. The obtained precipitate was filtered, redissolved in ethanol (10 mL) and recrystallized from slow evaporation of the solution, to give a light yellow solid which was collected by filtration and dried to a constant weight.

#### 5-Methyl-2-phenyl-4-(2-phenyl-1-(2-(4-(trifluoromethyl)phenyl)hydrazineyl)ethyl)-2,4-dihydro-3*H*-pyrazol-3-one (**H**_**2**_**L**^**1**^)

The proligand **H**_**2**_**L**^**1**^ was synthesized from 5-hydroxy-3-methyl-1-phenyl-1*H*-pyrazol-4-yl)(phenyl)methanone (HQ^Bn^) (500 mg, 1.710 mmol) and 4-trifluormethylphenylhydrazine (301 mg, 1.710 mmol), following the general procedure previously described (80 °C, reaction time 2 h). Yield: 68%, 531 mg, 1.18 mmol. **H**_**2**_**L**^**1**^ is a yellow powder soluble in DMSO, acetone, acetonitrile, alcohols, diethyl ether, and chlorinated solvents. mp: 195–196 °C. Anal. calcd for C_25_H_21_F_3_N_4_O; C, 66.66; H, 4.70; N, 12.65%. Found: C, 66.20; H, 4.60; N, 12.77%. IR (cm^–1^): 3214w ν(N–H), 3063w ν(C–H aromatic), 3130-2700wbr ν(O–H), 1619vs ν(C=N), 1590vs ν(C=N), 1534s ν(C=C), 1495s ν(C=C), 1488vs ν(C=C), 1327vs ν(C–F), 1317vs ν(C–F), 1112vs ν(C–F), 1064s ν(N–N). ^1^H NMR (CDCl_3_, 500 MHz, with 0.05% v/v TMS, 298 K): δ_H_ 12.39s (1H, O–*H*), 7.97d (2H, ^3^*J* = 8.7 Hz, *H*7 and *H*7′), 7.50–7.35m (4H, *H*8, *H*8′, *H*18 and *H*18′), 7.27–7.22m (3H, *H*14, *H*14′ and *H*15), 7.22–7.17m (1H, ^3^*J* = 8.7 Hz, *H*9), 7.16–7.10m (2H, *H*13 and *H*13′), 6.66d (2H, ^3^*J* = 8.2, Hz, *H*17 and *H*17′), 6.36s (1H, N–*H*), 4.13s (2H, *H*5), 2.38s (3H, *H*21). ^13^C{^1^H} NMR (CDCl_3_, 125 MHz, with 0.05% v/v TMS, 298): δ_C_ 164.2 (*C*5), 161.8 (*C*10), 145.2 (*C*3), 143.4 (*C*16), 138.5 (*C*6), 134.4 (*C*12), 129.19 (*C*14 and *C*14′), 128.9 (*C*8 and *C*8′), 127.9 (*C*13 and *C*13′), 127.4 (*C*15), 126.7q (^3^*J*_C–F_ = 3.9 Hz, *C*18 and *C*18′), 125.0 (*C*9), 123.7q (^2^*J*_C–F_ = 32.9 Hz, *C*19), 124.2q (^1^*J*_C–F_ = 270.6 Hz, *C*20), 119.5 (*C*7 and *C*7′), 112.6 (*C*17 and *C*17′), 100.1 (*C*4), 33.5 (*C*11), 16.7 (*C*21). ^19^F{^1^H} NMR (CDCl_3_, 125 MHz, with 0.05% v/v TMS, 298): δ_F_ 61.7. {^1^H,^15^N}gs- HSQC NMR (CDCl_3_, 51 MHz, ^3^*J*(N–H) = 3 Hz, 298 K): δN 96.2 (*N*4). {^1^H,^15^N} gs-HMBC NMR (CDCl_3_, 51 MHz, ^3^*J*(N–H) = 3 Hz, 298 K): δN 284.9 (*N*2), 140.6 (*N*3), 96.2 (*N*4), *N*1 not observed. ESI-MS (−) CH_3_CN (*m*/*z*, relative intensity %): 449 [100] [HL^1^]^−^. UV–visible (CH_3_CN, 10^–5^ M): 248 nm (π–π*), 299 nm (n−π*).

#### (*Z*)-5-Methyl-2-phenyl-4-(2-phenyl-1-(2-(pyridin-2-yl)hydrazineyl)ethylidene)-2,4-dihydro-3*H*-pyrazol-3-one (**H**_**2**_**L**^**2**^)

The proligand **H**_**2**_**L**^**2**^ was synthesized from (5-hydroxy-3-methyl-1-phenyl-1*H*-pyrazol-4-yl)(phenyl)methanone HQ^Bn^ (385 mg, 1.374 mmol) and 2-hydrazinopyridine (150 mg, 1.374 mmol), following the general procedure previously described (80 °C, reaction time 2 h). Yield: 65%, 333 mg, 0.85 mmol). **H**_**2**_**L**^**2**^ is a brown powder soluble in DMSO, acetone, acetonitrile, alcohols, diethyl ether, and chlorinated solvents. mp: 240–241 °C. Anal. calcd for C_23_H_21_N_5_O; C, 72.04; H, 5.52; N, 18.26%. Found: C, 71.96; H, 5.41; N, 18.33%. IR (cm^–1^): 3301w br ν(N–H), 3057w ν(C–H aromatic), 3027w ν(C–H aromatic), 1615s ν(C=N), 1592vs ν(C=N), 1538m ν(C=C), 1488m ν(C=C), 1472m ν(C=C), 1009m ν(N–N). ^1^H NMR (CDCl_3_, with 0.05% v/v TMS, 500 MHz, 298 K): δ_H_ 12.54s (1H, O–*H*), 8.10d (1H, ^3^*J* = 5.0 Hz, *H*20), 8.04d (2H, ^3^*J* = 7.8 Hz, *H*7,7′), 7.49t (1H, ^3^*J* = 7.4 Hz, *H*18), 7.42t (2H, ^3^*J* = 7.8 Hz, *H*8,8′), 7.31t (2H, ^3^*J* = 7.6 Hz, *H*13,13′), 7.26–7.21m (3H, *H*14,14′,15), 7.19t (1H, *H*9), 6.82t (1H, ^3^*J* = 7.3 Hz, *H*19), 6.56d (1H, ^3^*J* = 8.3 Hz, *H*17), 4.22s (2H, *H*11), 2.42s (3H, *H*21). ^13^C NMR (CDCl_3_, 125 MHz with 0.05% v/v TMS, 298): δ_C_ 167.1 (*C*5), 165.7 (*C*10), 157.9 (C16), 148.2 (C20), 147.0 (C3), 138.9 (C6), 138.5 (C18), 134.6 (C12),129.2 (C14,14′), 128.8 (C13,13′), 128.1 (C8,8′), 127.3 (C15), 124.6 (C9), 119.3 (C7,7′), 117.2 (C19), 106.8 (C17), 100.2 (C4), 33.6 (*C*11), 16.8 (C21). {^1^H,^15^N}gs- HSQC NMR (CDCl_3_, 51 MHz, ^3^*J*(N–H) = 3 Hz, 298 K): δN *N*4 not observed. {^1^H,^15^N} gs-HMBC NMR (CDCl_3_, 51 MHz, ^3^*J*(N–H) = 3 Hz, 298 K): δN 286.9 (*N*2), 139.4 (*N*3), *N*4, *N*1 not observed. ESI-MS (−) CH_3_CN (*m*/*z*, relative intensity %): 382 [100] [HL^2^]^−^. UV–visible (CH_3_CN, 10^–5^ M): 260 nm (π–π*), 304 nm (n−π*, >C=N−), 372 nm (n−π*, py).

### Synthesis of Zn(II) and Cu(II) Complexes **1–5**

#### [Zn(HL^1^)_2_(MeOH)_2_] (**1**)

A solution of Zn(OOCCH_3_)_2_·2H_2_O (29 mg, 0.133 mmol) in water (5 mL) was added to a solution of **H**_**2**_**L**^**1**^ (120 mg, 0.166 mmol) dissolved in methanol (15 mL). The mixture was stirred at reflux, and within an hour, a light yellow precipitate formed, which was removed by filtration, washed with a EtOH/H_2_O (60:40 v/v) solution and shown to be complex **1**. It is soluble in DMSO, DMF, acetonitrile, acetone, diethyl ether and chlorinated solvents. Yield = 86%, 118 mg, 0.114 mmol. mp 139–140 °C. Anal. calcd for C_52_H_48_F_6_N_8_O_4_Zn; C, 60.73; H, 4.70; N, 10.90%. Found: C, 60.68; H, 4.67; N, 10.81%. IR (cm^–1^): 3299m ν(N–H), 3.134w br ν(O–H···N), 3060w ν(C–H aromatic), 1603s ν(C=N), 1576s ν(C=N), 1532w ν(C=C), 1506s ν(C=C), 1479m ν(C=C), 1322vs ν(C–F), 1103vs ν(C–F), 1065s ν(N–N), 550m ν(Zn–N), 472s ν(Zn–O). ^1^H NMR (CDCl_3_ with 0.05% v/v TMS, 500 MHz, 298 K): δ_H_ 7.84d (4H, ^3^*J* = 8.0 Hz, *H*7 and *H*7′), 7.43t (4H, ^3^*J* = 7.8 Hz, *H*8 and *H*8′), 7.34–7.23m (12H, *H*14, *H*14′, *H*15, *H*18, *H*18′, *H*19), 7.03d (4H, ^3^*J* = 6.6 Hz, *H*13 and *H*13′), 6.46d (4H, ^3^*J* = 8.3 Hz, *H*17 and *H*17′), 5.98s (2H, N–*H*), 4.17s (4H, *H*11), 3.44s (6H, MeOH), 2.28s (6H, *H*21), 1.30s (4H, MeOH). ^13^C{^1^H} NMR (CDCl_3_ with 0.05% v/v TMS, 125 MHz): δ_C_ 177.7 (*C*5), 162.6 (*C*10), 149.1 (*C*3), 148.1 (*C*16), 138.5 (*C*6), 135.3 (*C*12), 129.1 (*C*14 and *C*14′), 128.7 (*C*8 and *C*8′), 127.9 (*C*13 and *C*13′), 127.0 (*C*15), 126.3q (^3^*J*_C–F_ = 3.4 Hz, *C*18 and *C*18′), 125.6 (*C*9), 124.3q (^1^*J*_C–F_ = 271.3 Hz, *C*20), 123.1q (^2^*J*_C–F_ = 32.3 Hz, *C*19), 119.5 (*C*7 and *C*7′), 114.1 (*C*17 and *C*7′), 98.3 (*C*4), 35.7 (*C*11), 17.3 (*C*21). ^19^F{^1^H} NMR (CDCl_3_, 125 MHz, with 0.05% v/v TMS, 298): δ_F_ 61.4. {^1^H,^15^N}gs- HSQC NMR (CDCl_3_, 51 MHz, ^3^*J*(N–H) = 3 Hz, 298 K): δN 117.3 (*N*4). {^1^H,^15^N} gs-HMBC NMR (CDCl_3_, 51 MHz, ^3^*J*(N–H) = 3 Hz, 298 K): δN 276.4 (*N*2), *N*3, *N*4, *N*1 not observed. ESI-MS (+) CH_3_CN (*m*/*z*, relative intensity %): 965 [100] [Zn(HL^1^)(**H**_**2**_**L**^**1**^)]^+^; 987 [40] [Zn(HL^1^)_2_ + Na]^+^; 1479 [30] [Zn_2_(HL^1^)_3_]^+^. UV–visible (CH_3_CN, 10^–5^ M): 249 nm (π–π*), 299 nm (n−π*), 388 nm sh (LMCT).

#### [Zn(HL^2^)_2_] (**2**)

A solution of Zn(OOCCH_3_)_2_·2H_2_O (29 mg, 0.133 mmol) in water (5 mL) was added to a solution of **H**_**2**_**L**^**2**^ (120 mg, 0.313 mmol) dissolved in methanol (15 mL). The mixture was stirred at room temperature and within an hour a light yellow precipitate formed, which was removed by filtration, washed with a EtOH/H_2_O (60:40 v/v) solution, and shown to be complex **2**. Yield: 78%, 102 mg, 0.122 mmol. It is soluble in DMSO, DMF, acetone, acetonitrile, and chlorinated solvents. mp: 172–174 °C. Anal. Calcd For C_46_H_40_N_10_O_2_Zn; C, 66.55; H, 4.86; N, 16.87%. Found: C, 63.65; H, 4.72; N, 15.64%. IR (cm^–1^): 3316w ν(N–H), 3187w br ν(N–H), 3056w ν(C–H aromatic), 1614s ν(C=N), 1593 ν(C=N), 1570s ν(C=N), 1055m ν(N–N), 540m ν(Zn–N), 444s ν(Zn–O). ^1^H NMR (CDCl_3_ with 0.05% v/v TMS, 500 MHz, 298 K): δ_H_ 8.05d (1H, ^3^*J* = 8.0 Hz, *H*20), 7.73 (2H, d, ^3^*J* = 8.0 Hz, *H*7 and *H*7′), 7.55 (1H, s, N–*H*), 7.50 (2H, d, ^3^*J* = 7.8 Hz, *H*13 and *H*13′), 7.44 (3H, m, *H*15, *H*14 and *H*14′), 7.38 (2H, d, ^3^*J* = 7.6 Hz, *H*18), 7.15 (2H, t, ^3^*J* = 8.0 Hz, *H*8 and *H*8′), 6.99 (1H, t, ^3^*J* = 8.0 Hz, *H*9), 6.52 (1H, t, ^3^*J* = 7.6 Hz, *H*19), 6.33 (1H, d, ^3^*J* = 8.0 Hz, *H*17), 4.41 (2H, dbr, *H*11), 2.42 (3H,s, *H*21). ^13^C NMR (CDCl_3_ with 0.05% v/v TMS, 125 MHz): δ_C_ 165.5 (C5), 162.2 (C10), 152.6 (C16), 146.7 (C20), 144.6 (C3), 139.0 (C6), 138.5 (C18), 134.6 (C12), 129.8 (C15), 128.8 (C14,14′), 128.2 (C8,8′), 128.0 (C13,13′), 124.6 (C9), 119.3 (C7,7′), 115.4 (C19), 109.3 (C17), 97.6 (C4), 35.4 (C11), 17.2 (C21). {^1^H,^15^N}gs- HSQC NMR (CDCl_3_, 51 MHz, ^3^*J*(N–H) = 3 Hz, 298 K): δN 130.2 (*N*4). {^1^H,^15^N} gs-HMBC NMR (CDCl_3_, 51 MHz, ^3^*J*(N–H) = 3 Hz, 298 K): δN 51.2 (*N*5), *N*3, *N*2, and *N*1 not observed. ESI-MS (+) CH_3_CN (*m*/*z*, relative intensity %): 829 [70] [Zn(HL^2^)(**H**_**2**_**L**^**2**^)]^+^; 851 [100] [Zn(HL^2^)_2_ + Na]^+^; 1276 [55] [Zn_2_(HL^2^)_3_]^+^. UV–visible (CH_3_CN, 10^–5^ M): 266 nm (π–π*), 305 nm (n−π*, >C=N−), 371 nm (n−π*, py), 406 nm sh (LMCT).

#### [Cu(HL^1^)_2_] (**3**)

A solution of Cu(OOCCH_3_)_2_·2H_2_O (26 mg, 0.133 mmol) in water (5 mL) was added to a solution of **H**_**2**_**L**^**1**^ (120 mg, 0.166 mmol) dissolved in methanol (15 mL). The mixture was stirred at room temperature, and immediately a red precipitate formed, which was removed by filtration, washed with a EtOH/H_2_O (60:40 v/v) solution, and shown to be complex **3**. It is soluble in DMSO, DMF, acetone, and chlorinated solvents. Yield: 63%, 80 mg, 0.084 mmol. Mp: 205–208 °C. Anal. calcd. for C_50_H_40_CuF_6_N_8_O_2_; C, 62.40; H, 4.19; N, 11.64%. Found: C, 62.19; H, 4.24; N, 11.32%. IR (cm^–1^): 3294m ν(N–H), 3026w ν(C_arom_–H), 3028w ν(C_arom_–H), 1614m ν(C=N), 1601m ν(C=N), 1588m ν(C=N), 1575m ν(C=C), 1516vs ν(C=C), 1489vs ν(C=C), 1327vs ν(C–F), 1103vs ν(C–F), 1065s ν(N–N), 540m ν(Cu–N), 510s ν(Cu–O). ESI-MS (+) CH_3_CN (*m*/*z*, relative intensity %): 963 [15] [Cu(HL^1^)(**H**_**2**_**L**^**1**^)]^+^; 1067 [20] [Cu(HL^1^)(**H**_**2**_**L**^**1**^)(MeOH)_2_(MeCN)]^+^. UV–visible (CH_3_CN, 10^–5^ M): 259 nm (π–π*), 278 nm (n−π*), 392 nm sh (LMCT).

#### [Cu(HL^2^)_2_] (**4**)

A solution of Cu(OOCCH_3_)_2_·2H_2_O (31 mg, 0.157 mmol) in water (5 mL) was added to a solution of **H**_**2**_**L**^**2**^ (120 mg, 0.313 mmol) dissolved in methanol (15 mL). The mixture was stirred at room temperature, and immediately, a brown green precipitate formed, which was removed by filtration, washed with a EtOH/H_2_O (60:40 v/v) solution, and shown to be complex **4**. It is soluble in DMSO, DMF, acetone, acetonitrile, and chlorinated solvents. mp: 181–182 °C. Anal. calcd. for C_46_H_40_CuN_10_O_2_; C, 66.69; H, 4.87; N, 16.91%. Found: C, 66.23; H, 4.75; N, 16.86%. IR (cm^–1^): 3297w br ν(N–H), 3062w ν(N–H), 3030w ν(C–H aromatic), 1617m ν(C=N), 1588m ν(C=N), 1572 ν(C=N), 1528m, 1508m ν(C=C), 1071m ν(N–N), 546m ν(Cu–N), 508s ν(Cu–O). ESI-MS (+) CH_3_CN (*m*/*z*, relative intensity %): 828 [100] [Cu(HL^2^)(**H**_**2**_**L**^**2**^)]^+^; 1274 [75] [Cu_2_(HL^2^)_3_]^+^. UV–visible (CH_3_CN, 10^–5^ M): 256 nm (π–π*), 305 nm (n−π*, >C=N−), 378 nm (n−π*, py), 414 nm sh (LMCT).

#### [Cu(Q^Bn^)_2_] (**5**)

Complex 5 is obtained with a procedure similar to that of 3 but leaving the reaction mixture under stirring for 24 h, during which the red precipitate slowly converts to dark green. It is soluble in DMSO, DMF, acetone, and chlorinated solvents. Yield: 86%, 74 mg, 0.114 mmol. mp: 264–266 °C. Anal. calcd. For C_36_H_30_CuN_4_O_4_; C, 66.91; H, 4.68; N, 8.67%. Found: C, 66.55; H, 4.57; N, 8.71%. IR (cm^–1^): 3065w ν(C_arom_–H), 3037w ν(C_arom_–H), 1604s ν(C=O), 1590s ν(C=N), 1575vs ν(C=C), 1532m ν(C=C), 1486vs ν(C=C), 510s, 400m ν(Cu–O). ESI-MS (+) CH_3_CN (*m*/*z*, relative intensity %): 647 [100] [Cu(Q^Bn^)(HQ^Bn^)]^+^.

## Supporting Information Available

NMR and IR spectra, X-ray data collection and structure refinements, DFT data of the optimized structures of proligands and metal complexes, comparison of calc. and exp. IR spectra, ^1^H and ^13^C NMR spectra, MOs of anionic ligands [HL^1^]^−^ and [HL^2^]^−^ and coordinates, and HPLC chromatographs showing NTPs, dNTPs, NDPs and dNDPs (PDF)
