# Supplementary material for: Zinc(II) Complex with Pyrazolone-Based Hydrazones is Strongly Effective against Trypanosoma brucei Which Causes African Sleeping Sickness
Source: Inorg Chem. 2022 Aug 15;61(34):13561–75. doi: 10.1021/acs.inorgchem.2c02201 (PMC9446893; doi:10.1021/acs.inorgchem.2c02201)
Supplement: Supplementary file 1 — ic2c02201_si_001.pdf [file ic2c02201_si_001.pdf]

SUPPORTING INFORMATION

A Zinc(II) complex with pyrazolone-based hydrazones is strongly effective against *Trypanosoma brucei*, which causes African sleeping sickness

Fabio Marchetti,[#*] Alessia Tombesi,[†] Corrado Di Nicola,[#] Riccardo Pettinari,[†] Federico Verdicchio,[†] Alessandra Crispini,[Ψ] Francesca Scarpelli,[Ψ] Cecilia Baldassarri,[†] Elisa Marangoni,[†] Anders Hofer,[ϕ] Agustín Galindo,[‡] Riccardo Petrelli[†]

[†] Chemistry Interdisciplinary Project (CHIP), School of Pharmacy, University of Camerino, via Madonna delle Carceri, 62032 Camerino MC, Italy;

[#] Chemistry Interdisciplinary Project (CHIP), School of Science and Technology, University of Camerino, via Madonna delle Carceri, 62032 Camerino MC, Italy;

[Ψ] MAT-InLAB, Dipartimento di Chimica e Tecnologie Chimiche, Università della Calabria, 87036 Arcavacata di Rende, CS,Italy;

[ϕ] Department of Medical Biochemistry and Biophysics, Umea University, 901 87 Umeå, Sweden.

[‡] Departamento de Química Inorgánica, Facultad de Química, Universidad de Sevilla, Aptdo 1203, 41071 Sevilla, Spain.

E-mail: fabio.marchetti@unicam.it

Table of contents

Figure S1. ^{1}H NMR spectrum in $CDCl_3$ at 298 K of $\mathbf{H_2L^1}$. S3
Figure S2. {^{1}H,^{1}H}-COSY spectrum in $CDCl_3$ at 298 K of $\mathbf{H_2L^1}$. S3
Figure S3. ^{13}C{^{1}H} NMR spectrum in $CDCl_3$ at 298 K of $\mathbf{H_2L^1}$. S4
Figure S4. Magnification of ^{13}C{^{1}H} NMR spectrum in $CDCl_3$ at 298 K of $\mathbf{H_2L^1}$. S4
Figure S5. {^{1}H,^{13}C}-HSQC spectrum in $CDCl_3$ at 298 K of $\mathbf{H_2L^1}$. S5
Figure S6. {^{1}H,^{13}C}-HMBC spectrum in $CDCl_3$ at 298 K of $\mathbf{H_2L^1}$. S5
Figure S7. {^{1}H,^{15}N}-HSQC spectrum in $CDCl_3$ at 298 K of $\mathbf{H_2L^1}$. S6
Figure S8. {^{1}H,^{15}N}-HMBC spectrum in $CDCl_3$ at 298 K of $\mathbf{H_2L^1}$. S6
Figure S9. ^{19}F{^{1}H} NMR spectrum in $CDCl_3$ at 298 K of $\mathbf{H_2L^1}$. S7
Figure S10. IR–MIR spectrum of $\mathbf{H_2L^1}$. S7
Figure S11. IR–FIR spectrum of $\mathbf{H_2L^1}$. S8
Figure S12. ^{1}H NMR spectrum in $CDCl_3$ at 298 K of $\mathbf{H_2L^2}$. S8
Figure S13. {^{1}H,^{1}H}-COSY spectrum in $CDCl_3$ at 298 K of $\mathbf{H_2L^2}$. S9
Figure S14. ^{13}C{^{1}H} NMR spectrum in $CDCl_3$ at 298 K of $\mathbf{H_2L^2}$. S9
Figure S15. {^{1}H,^{13}C}-HSQC spectrum in $CDCl_3$ at 298 K of $\mathbf{H_2L^2}$. S10
Figure S16. {^{1}H,^{13}C}-HMBC spectrum in $CDCl_3$ at 298 K of $\mathbf{H_2L^2}$. S10
Figure S17. {^{1}H,^{15}N}-HMBC spectrum in $CDCl_3$ at 298 K of $\mathbf{H_2L^2}$. S11
Figure S18. IR–MIR spectrum of $\mathbf{H_2L^2}$. S11
Figure S19. IR–FIR spectrum of $\mathbf{H_2L^2}$. S12
Figure S20. ^{1}H NMR spectrum in $CDCl_3$ at 298 K of $[Zn(HL^1)_2(MeOH)_2]$ (**1**). S12
Figure S21. ^{13}C NMR spectrum in $CDCl_3$ at 298 K of $[Zn(HL^1)_2(MeOH)_2]$ (**1**). S13
Figure S22. Magnification of ^{13}C NMR spectrum of $[Zn(HL^1)_2(MeOH)_2]$ (**1**). S13

Figure S23. {^{1}H,^{15}N}-HSQC spectrum in $CDCl_3$ at 298 K of [Zn(HL^1)$_2$(MeOH)$_2$] (**1**). ………..… S14
Figure S24. {^{1}H,^{15}N}-HMBC spectrum in $CDCl_3$ at 298 K of [Zn(HL^1)$_2$(MeOH)$_2$] (**1**). ………... S14
Figure S25. ^{19}F{^{1}H} NMR spectrum in $CDCl_3$ at 298 K of [Zn(HL^1)$_2$(MeOH)$_2$] (**1**). …………..... S15
Figure S26. IR–MIR spectrum of [Zn(HL^1)$_2$(MeOH)$_2$] (**1**)……………………………………… S15
Figure S27. IR–FIR spectrum of [Zn(HL^1)$_2$(MeOH)$_2$] (**1**). ……………………………………... S16
Figure S28. ^{1}H NMR spectrum in $CDCl_3$ at 298 K of [Zn(HL^2)$_2$] (**2**). …………………………... S16
Figure S29. ^{13}C NMR spectrum in CDCl3 at 298 K of [Zn(HL^2)$_2$] (**2**). …………………….…. S17
Figure S30. {^{1}H,^{13}C}-HSQC spectrum in CDCl3 at 298 K of [Zn(HL^2)$_2$] (**2**). ...…..………….….. S17
Figure S31. {^{1}H,^{15}N}-HSQC spectrum in CDCl3 at 298 K of [Zn(HL^2)$_2$] (**2**). ………..………… S18
Figure S32. {^{1}H,^{15}N}-HMBC spectrum in CDCl3 at 298 K of [Zn(HL^2)$_2$] (**2**). ………………..... S18
Figure S33. IR–MIR spectrum of [Zn(HL^2)$_2$] (**2**). ………………………………….……... S19
Figure S34. IR–FIR spectrum of [Zn(HL^2)$_2$] (**2**). ……………………………………………… S19
Figure S35. IR–MIR spectrum of [Cu(HL^1)$_2$] (**3**). ……………………………………….……… S20
Figure S36. IR–FIR spectrum of [Cu(HL^1)$_2$] (**3**). ………………………………………………... S20
Figure S37. IR–MIR spectrum of [Cu(HL^2)$_2$] (**4**). ……………………………………….……… S21
Figure S38. IR–FIR spectrum of [Cu(HL^2)$_2$] (**4**). ……………………………………….……….. S21
Figure S39. ESI-MS spectra of (a) [Zn(HL^1)$_2$(MeOH)$_2$] (**1**)and (b) [Cu(HL^2)$_2$] (**4**). …………..... S22
Figure S40. Crystal packing view of **H_2L^2**. …………………………………………...…. S22
Table S1. Details of data collection and structure refinements for proligands **H_2L^1** and **H_2L^2**. …. S23
Table S2. Details of data collection and structure refinements for complexes **1** and **5**. ………..... S24
Table S3. Energies for the tautomers of proligands **H_2L^1** and **H_2L^2**. …………………………….. S25
Figure S41. Comparison of calc. (tautomer I) and exp. ^{1}H and ^{13}C NMR of **H_2L^1.** ……..…… S26
Figure S42. Comparison of selected bond distances of **H_2L^1** and **H_2L^2**. ....……..… S27
Figure S43. Comparison of the exp. and calc. IR spectrum of **H_2L^1** (cm^{-1}). ....………..... S28
Table S4. Selected stretching assignments of the IR spectrum of **H_2L^1**. ………………………… S28
Figure S44. MOs of anionic ligands [HL^1]$^-$ and [HL^2]$^-$. …………………………………………….. S29
Table S5. Comparison of selected exp. and calc. structural parameters of complex **1**. ...……….. S30
Figure S45. Comparison of calc. and exp. ^{1}H and ^{13}C NMR spectra of **1** and **2**. ……..……..... S31
Figure S46. Optimized structures of complexes [Zn(HL^1)$_2$] and [Zn(HL^3)$_2$]. …………………… S32
Table S6. Comparison of selected experimental and calculated structural parameters of **5**. …….. S33
Table S7. Coordinates of the optimized compounds. …………………………………..…… S34-S50
Figure S47. HPLC chromatographs showing NTPs, dNTPs, NDPs and dNDPs. … … … … … ….. S51
Figure S48. ^{1}H NMR spectra in DMSO-d$_6$ of [Zn(HL^1)$_2$(MeOH)$_2$] (**1**) at t = 0, 24 h and 48 h. S52
Figure S49. ^{1}H NMR spectra in DMSO-d$_6$ of [Zn(HL^2)$_2$] (**2**) at t = 0, 24 h and 48 h. ….............. S53

NMR spectra of H_2L^1

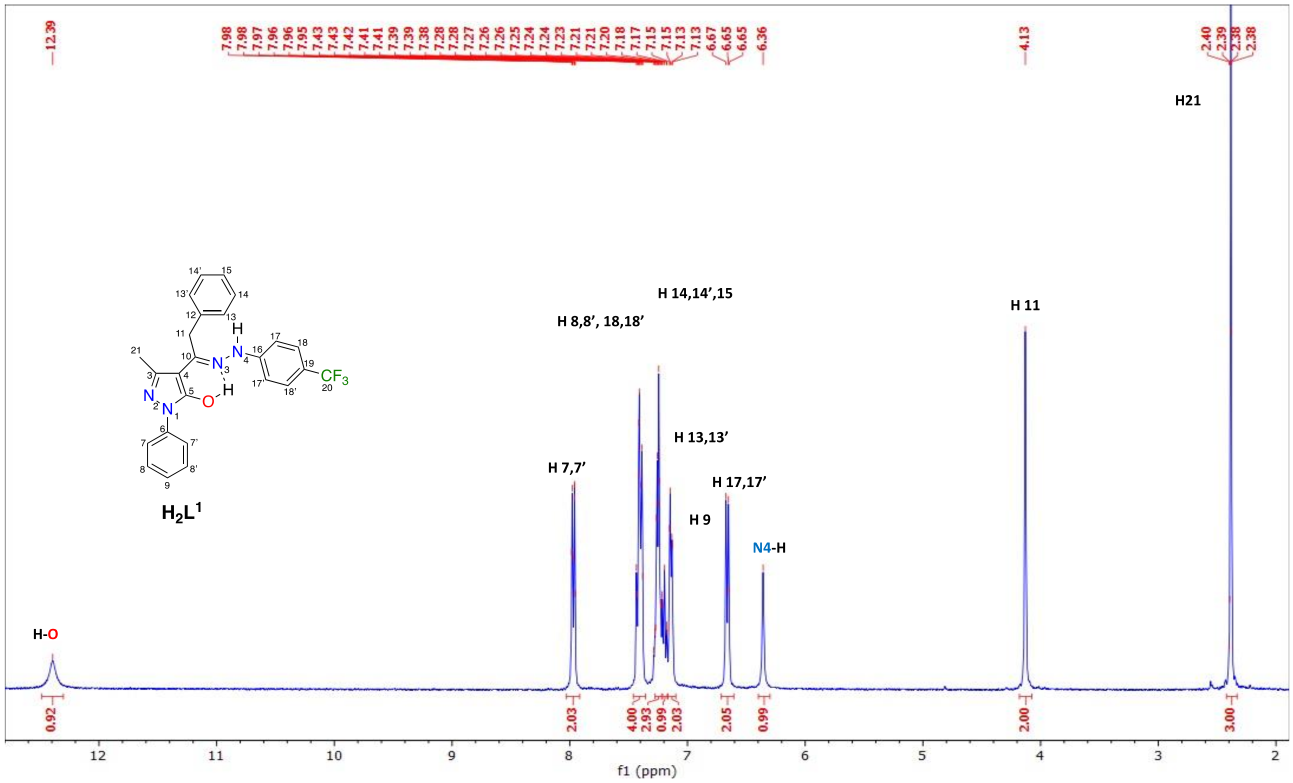

Figure S1. ^1H NMR spectrum in $CDCl_3$ at 298 K of H_2L^1.

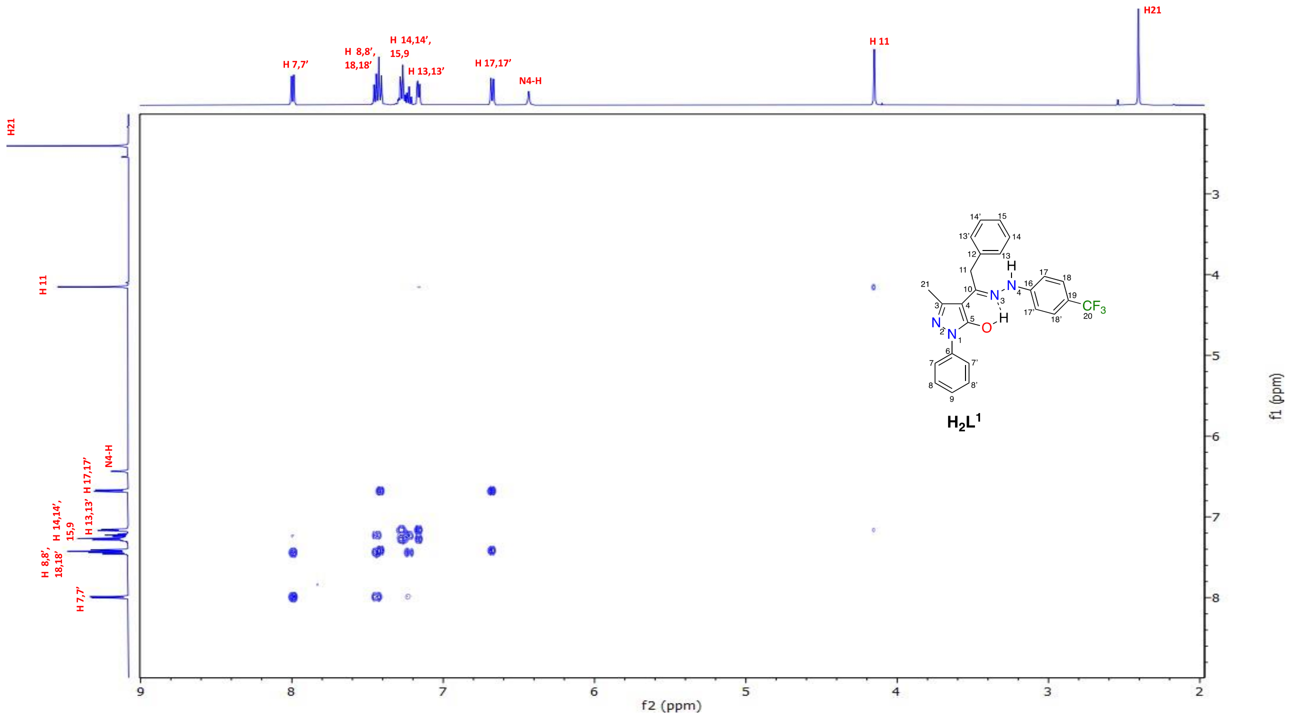

Figure S2. {^1H,^1H}-COSY spectrum in $CDCl_3$ at 298 K of H_2L^1.

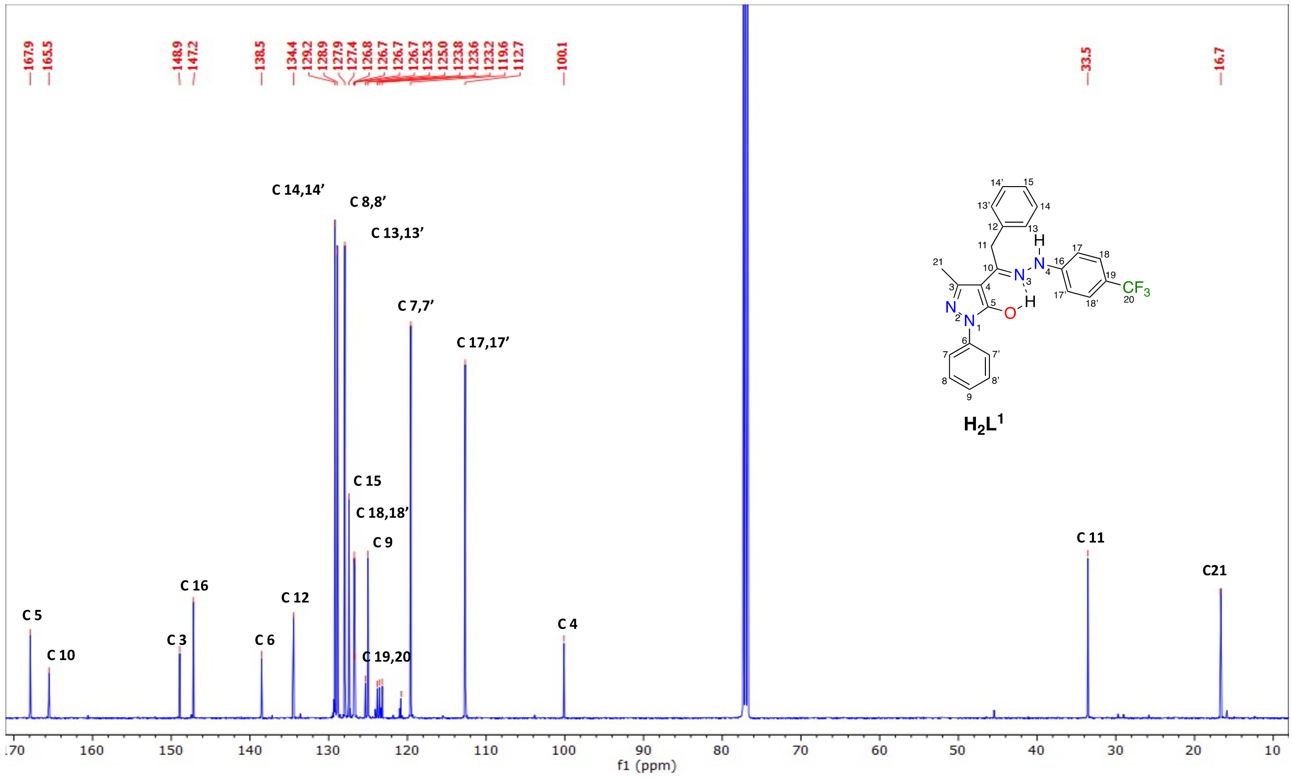

Figure S3. $^{13}C\{^{1}H\}$ NMR spectrum in $CDCl_3$ at 298 K of H_2L^1.

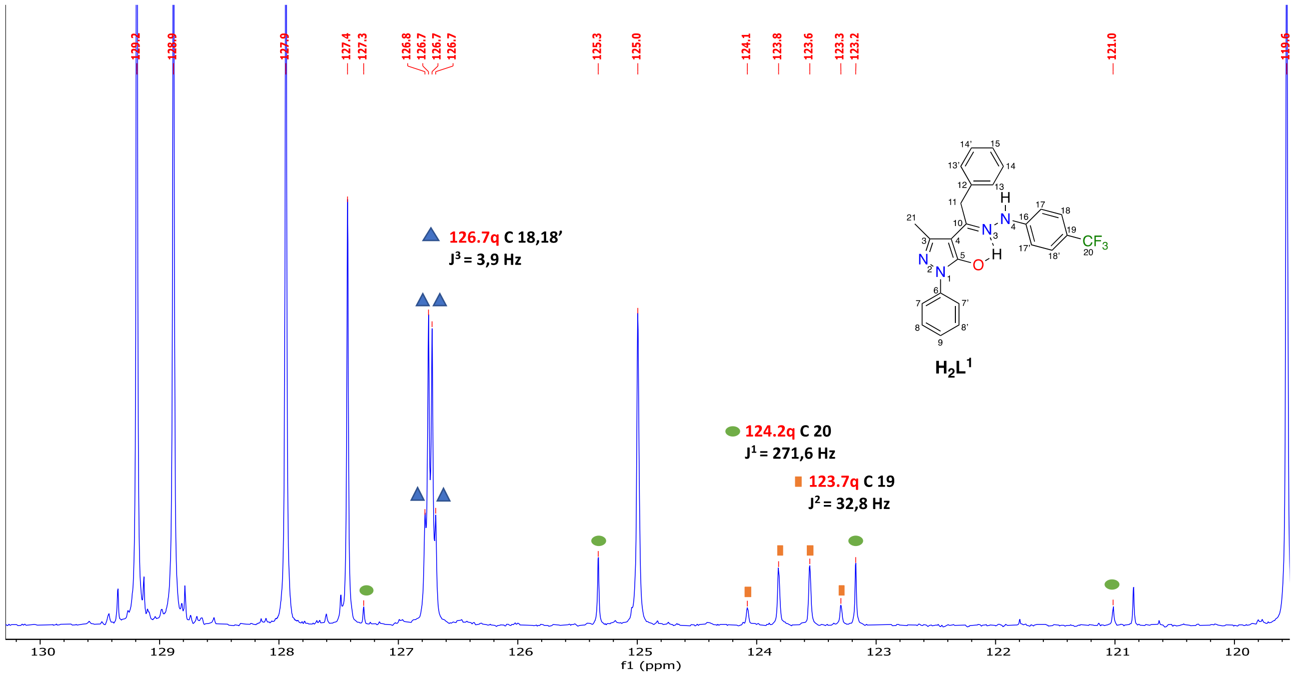

Figure S4. Magnification of $^{13}C\{^{1}H\}$ NMR spectrum in $CDCl_3$ at 298 K of H_2L^1.

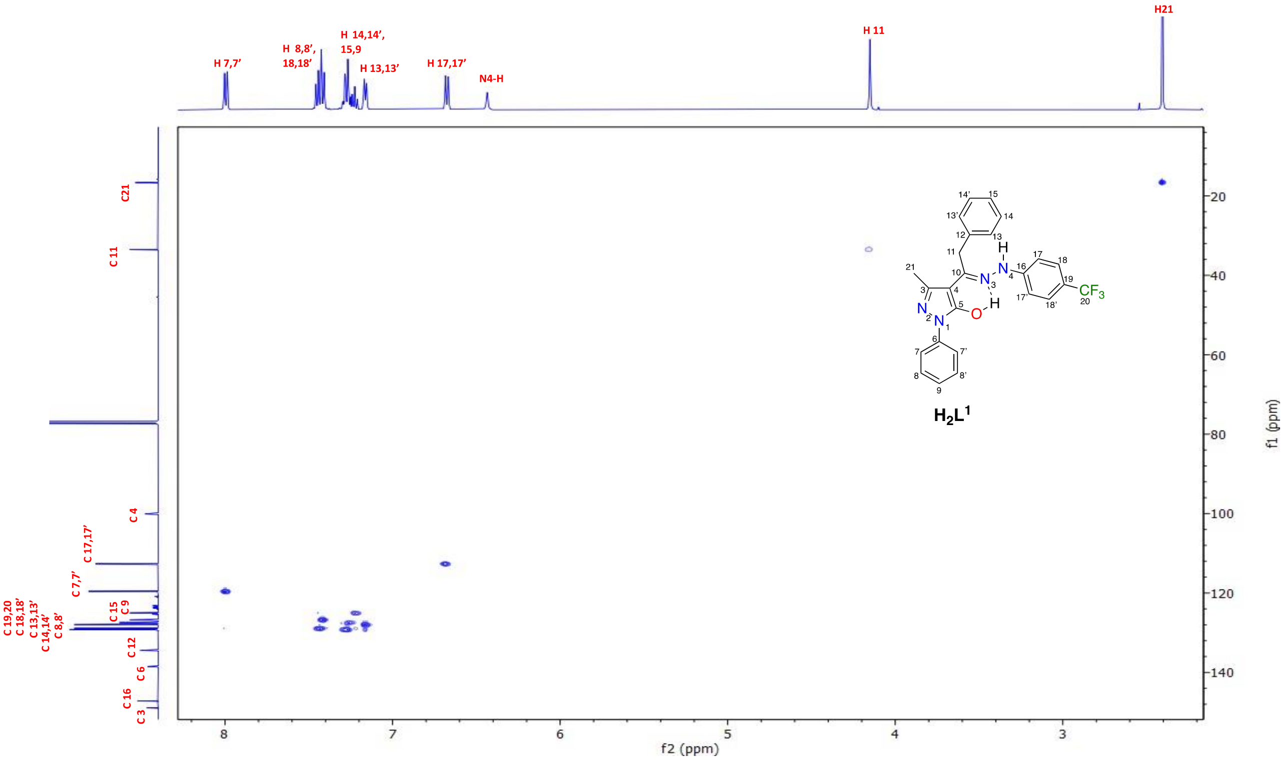

Figure S5. {^{1}H,^{13}C}-HSQC spectrum in $CDCl_3$ at 298 K of H_2L^1.

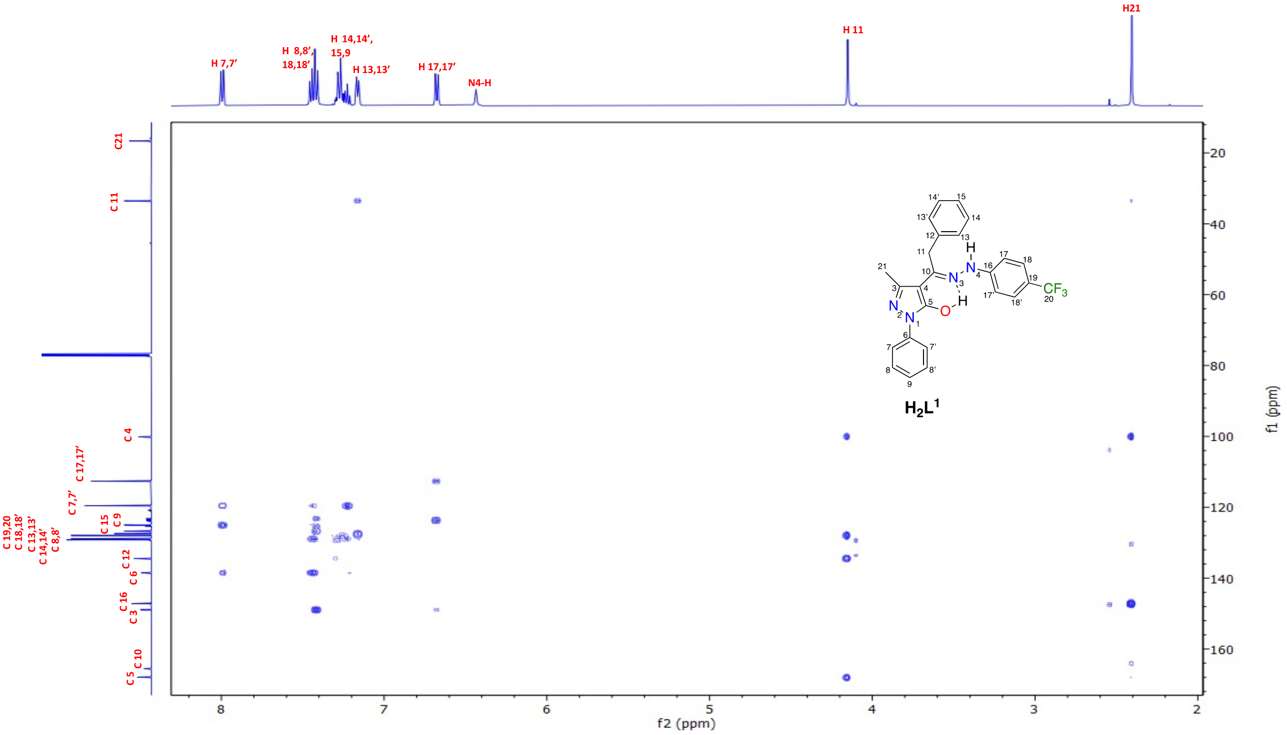

Figure S6. {^{1}H,^{13}C}-HMBC spectrum in $CDCl_3$ at 298 K of H_2L^1.

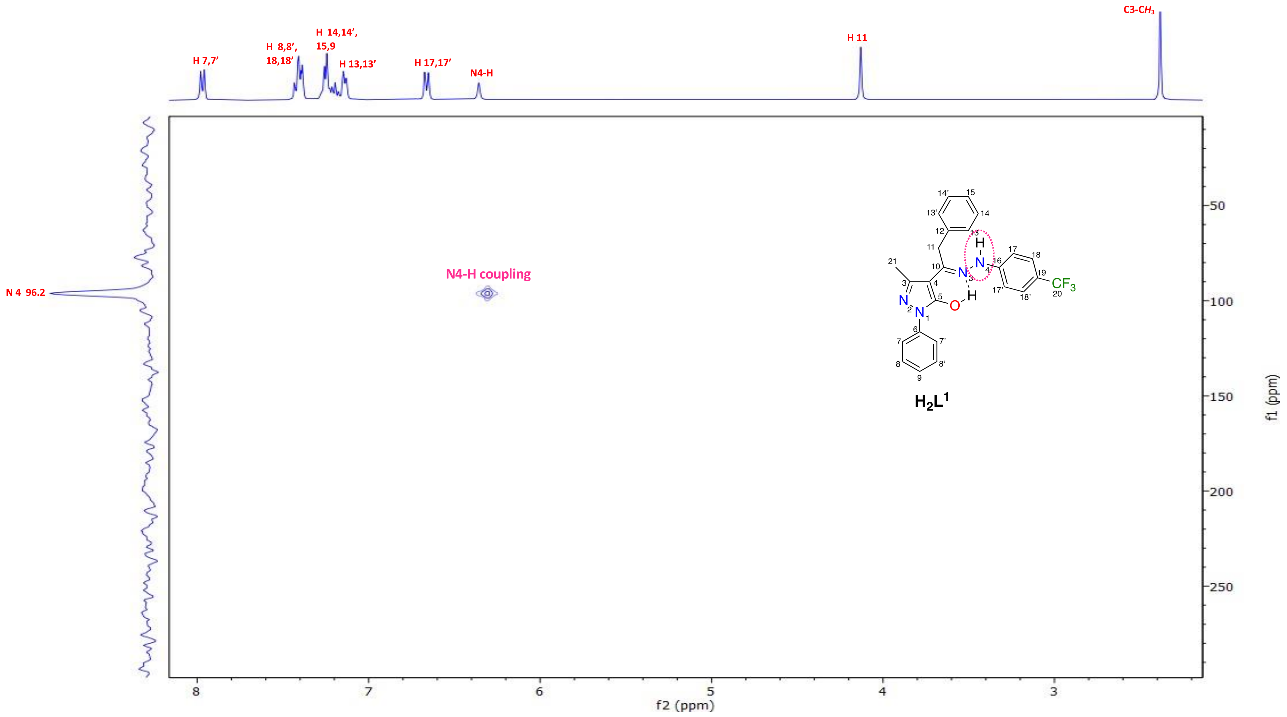

Figure S7. {^{1}H,^{15}N}-HSQC spectrum in $CDCl_3$ at 298 K of H_2L^1.

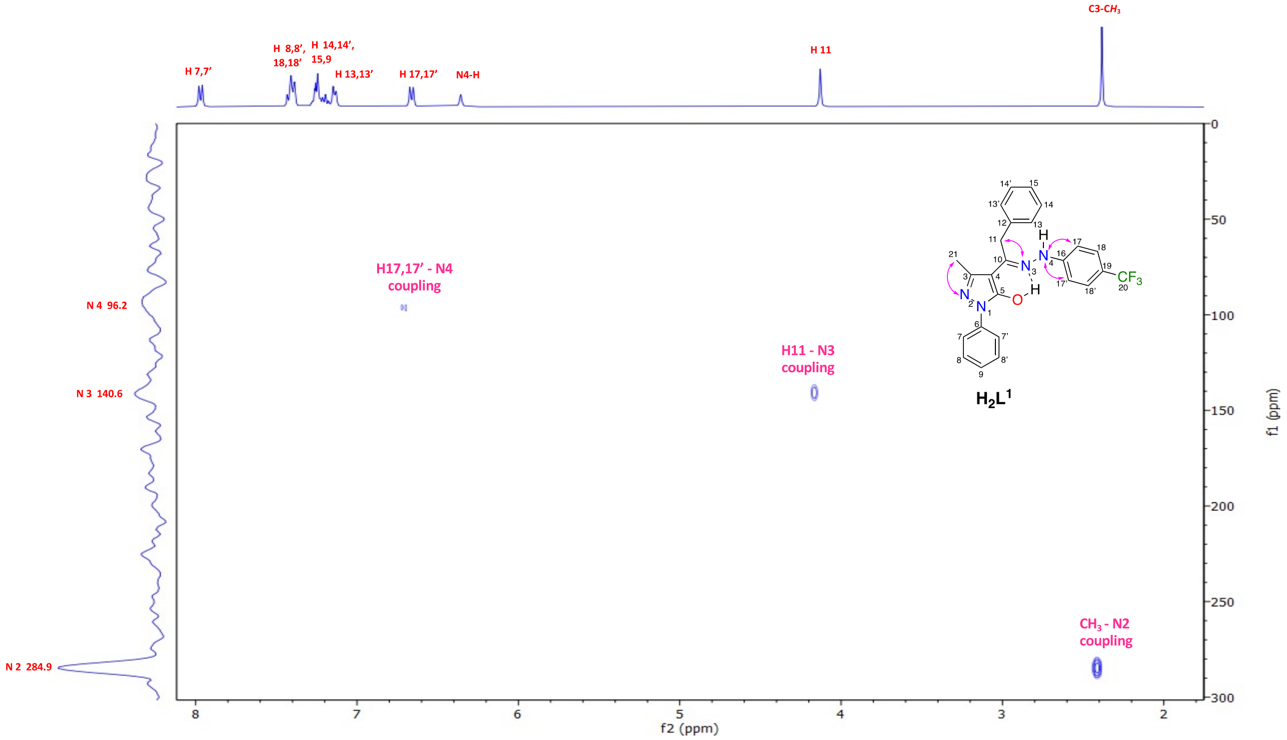

Figure S8. {^{1}H,^{15}N}-HMBC spectrum in $CDCl_3$ at 298 K of H_2L^1.

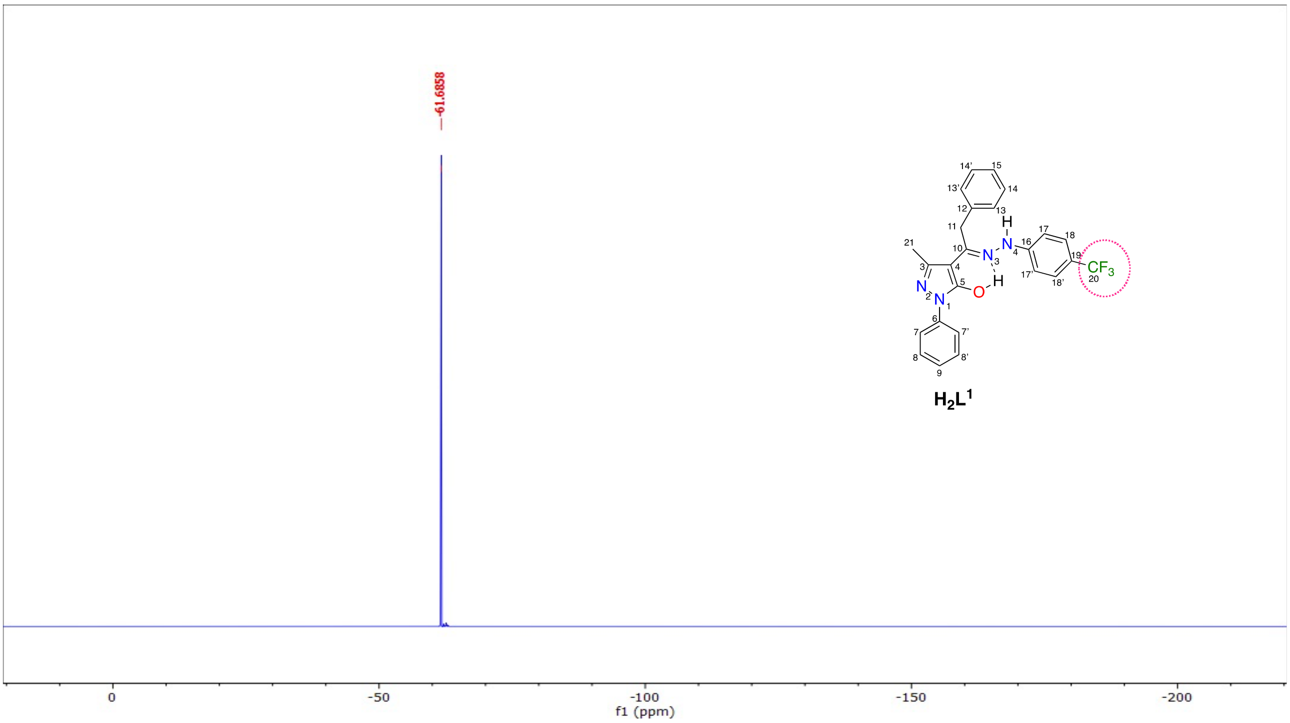

Figure S9. $^{19}F\{^{1}H\}$ NMR spectrum in $CDCl_3$ at 298 K of H_2L^1.

IR spectra of H_2L^1

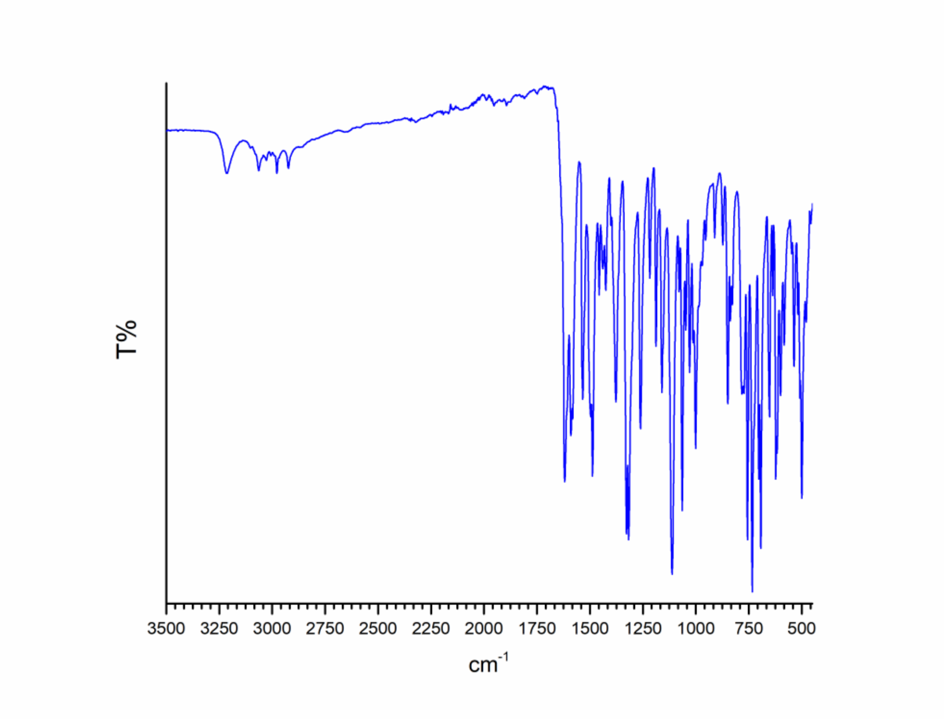

Figure S10 MIR spectrum of H_2L^1.

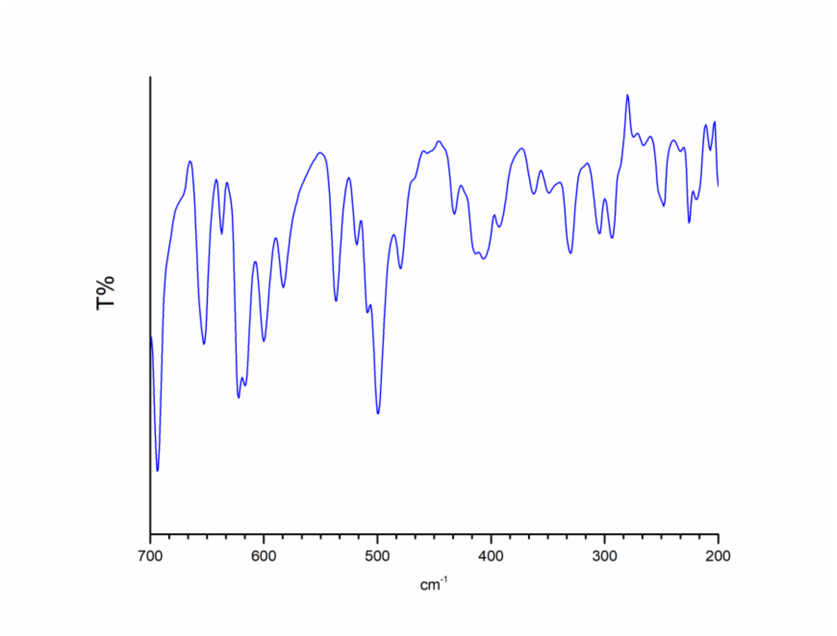

Figure S11 FIR spectrum of H_2L^1.

NMR spectra of H_2L^2

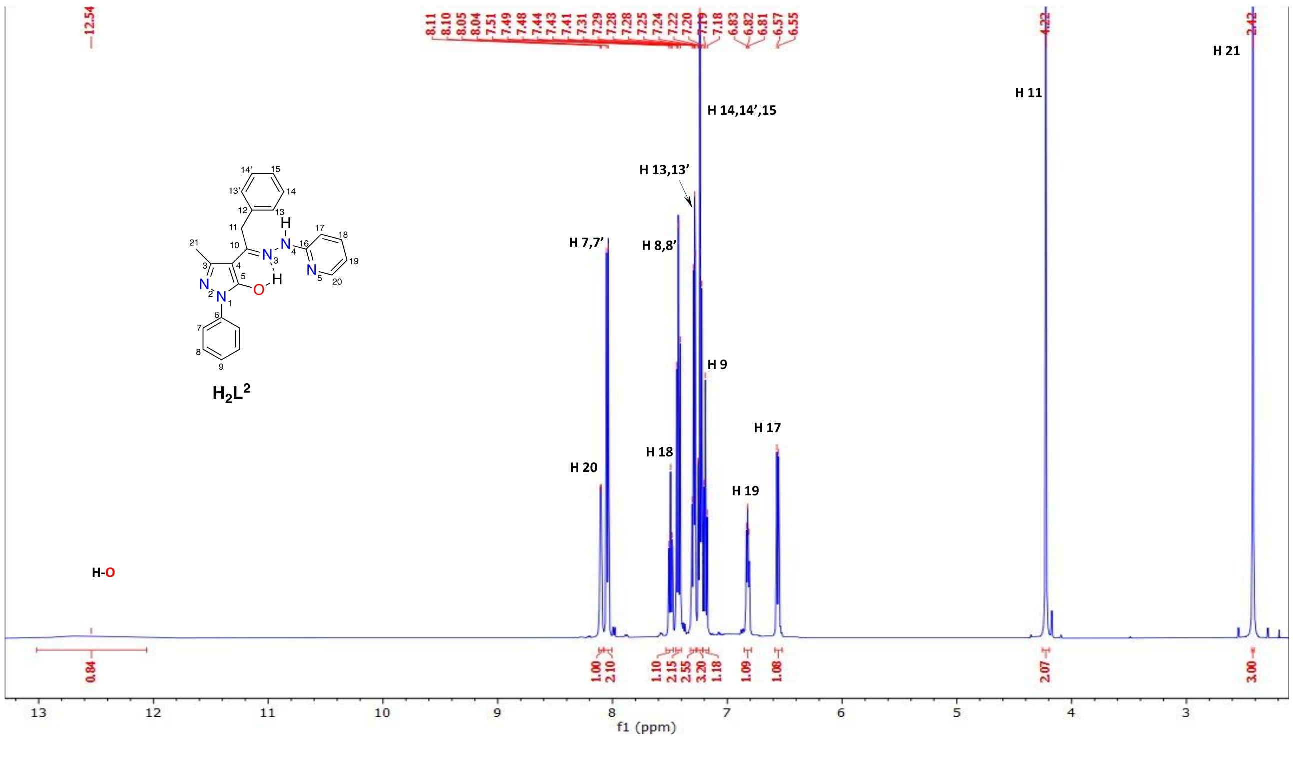

Figure S12. ^1H NMR spectrum in $CDCl_3$ at 298 K of H_2L^2.

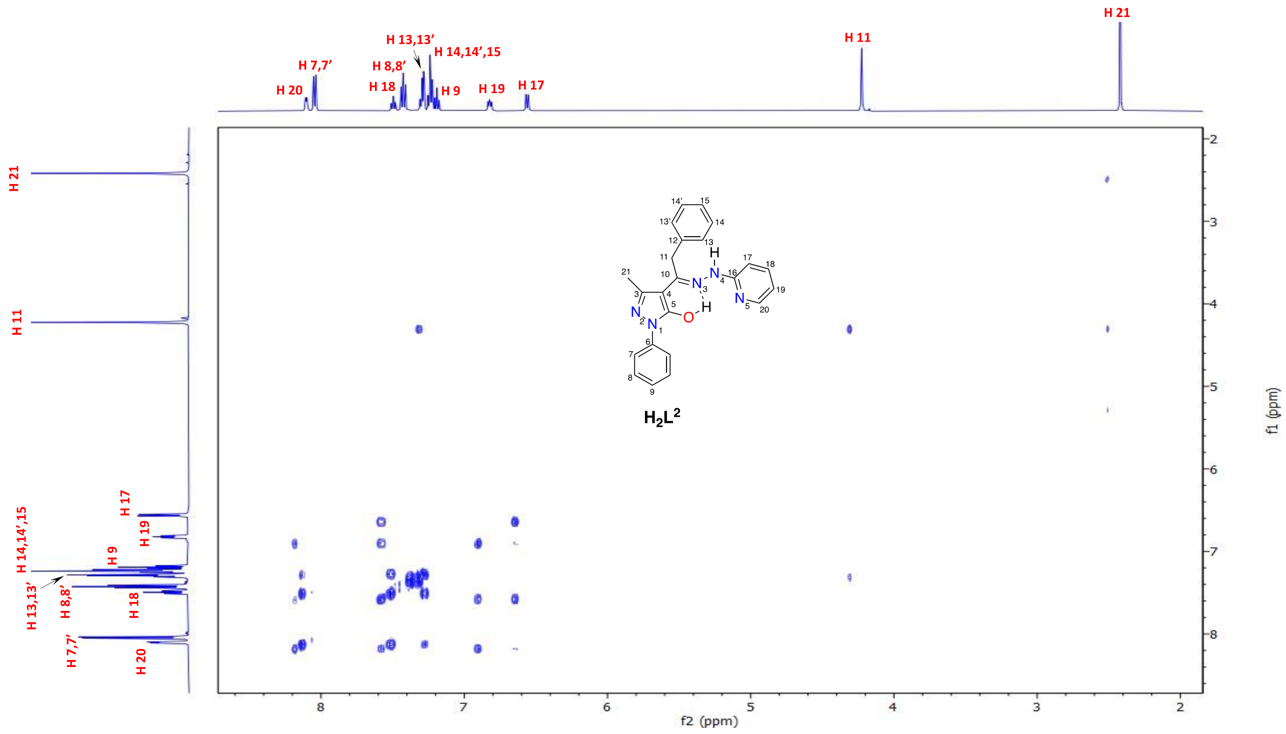

Figure S13. {^{1}H,^{1}H}-COSY spectrum in $CDCl_3$ at 298 K of H_2L^2.

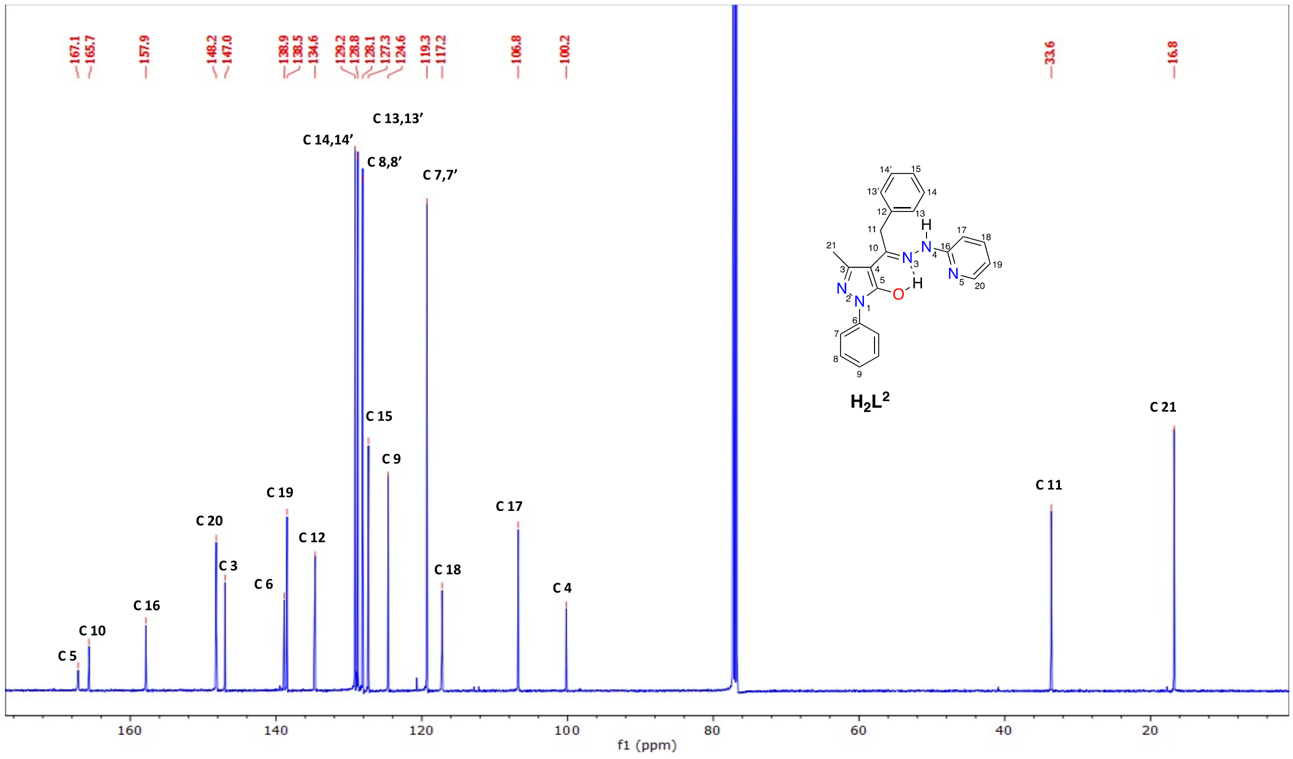

Figure S14. ^{13}C{^{1}H} NMR spectrum in $CDCl_3$ at 298 K of H_2L^2.

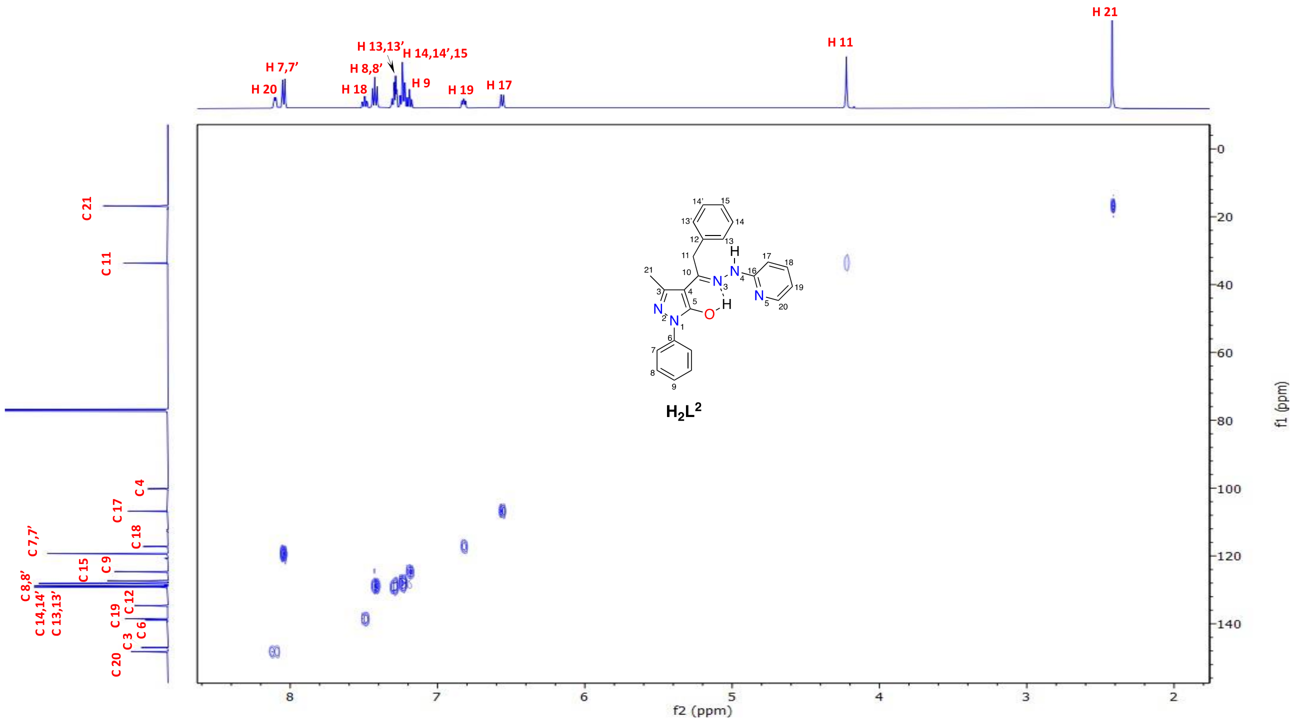

Figure S15. {^{1}H,^{13}C}-HSQC spectrum in $CDCl_3$ at 298 K of H_2L^2.

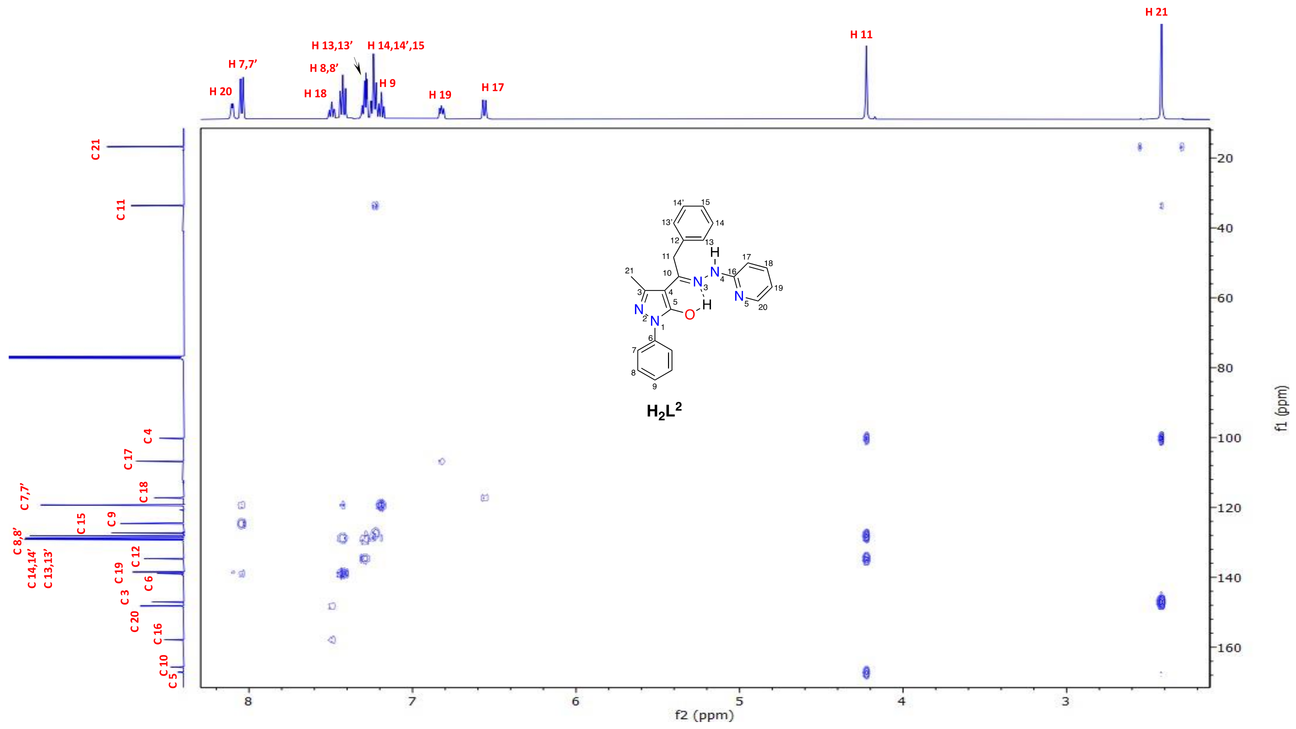

Figure S16. {^{1}H,^{13}C}-HMBC spectrum in $CDCl_3$ at 298 K of H_2L^2.

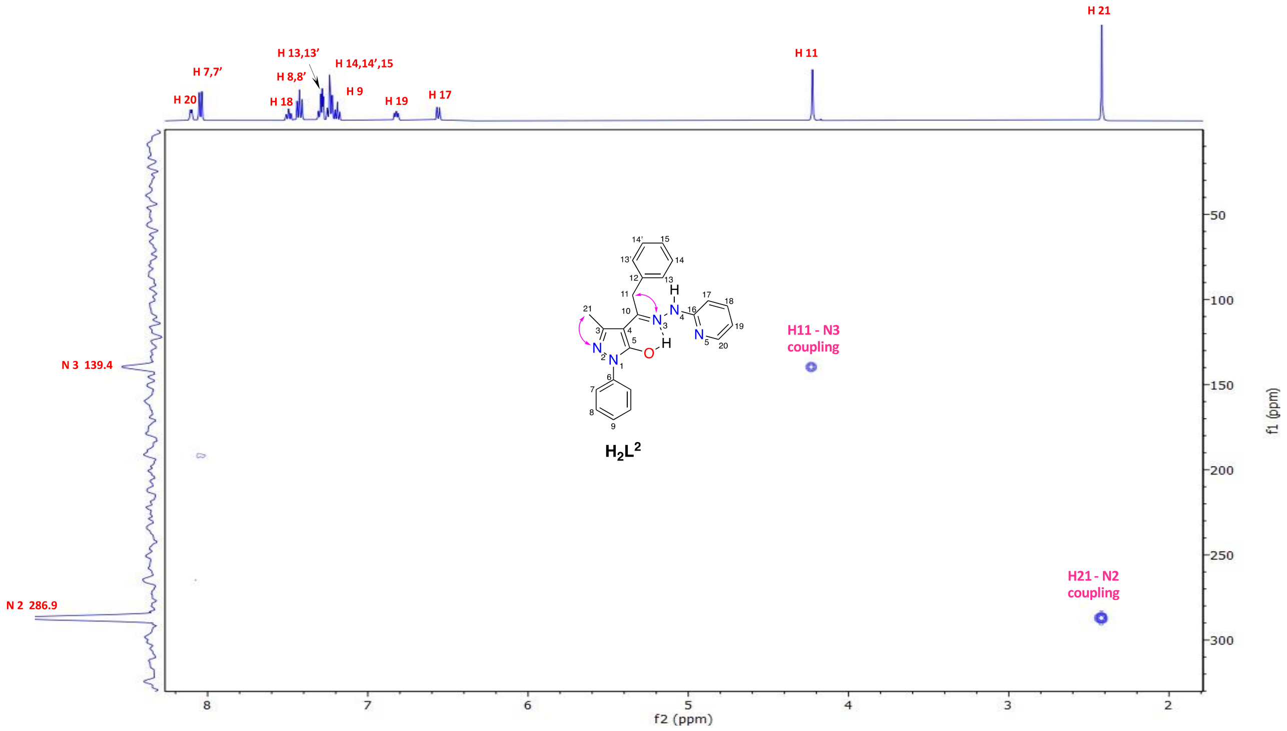

Figure S17. {^{1}H,^{15}N}-HMBC spectrum in $CDCl_3$ at 298 K of H_2L^2.

IR spectra of H_2L^1

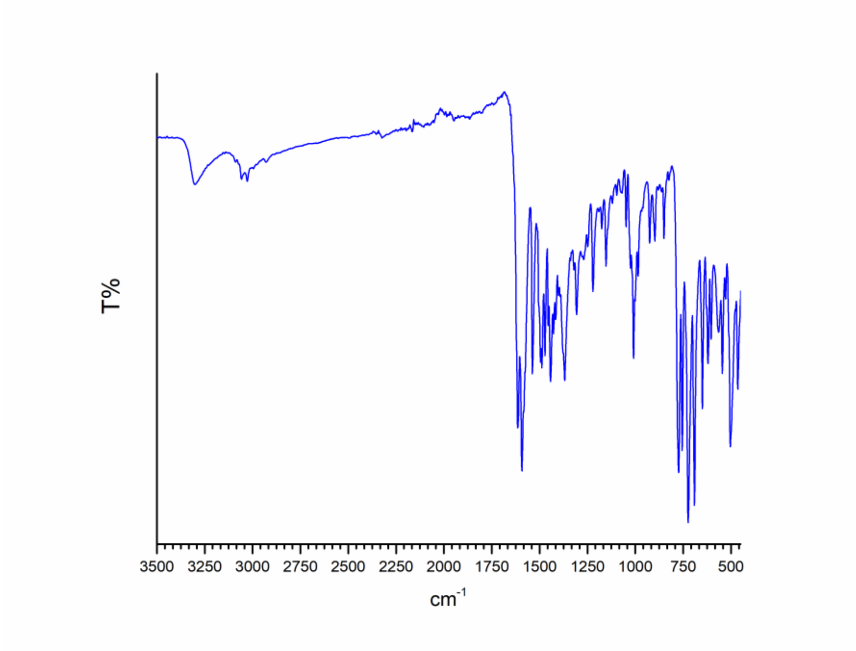

Figure S18 MIR spectrum of H_2L^2.

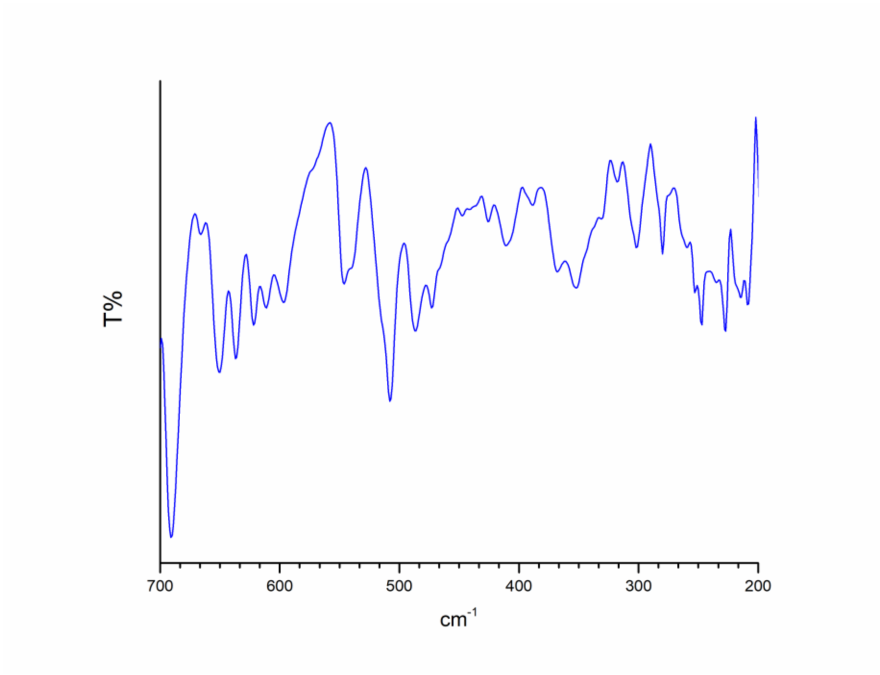

Figure S19 FIR spectrum of H_2L^1.

NMR spectra of [Zn(HL[1])$_2$(MeOH)$_2$] (1)

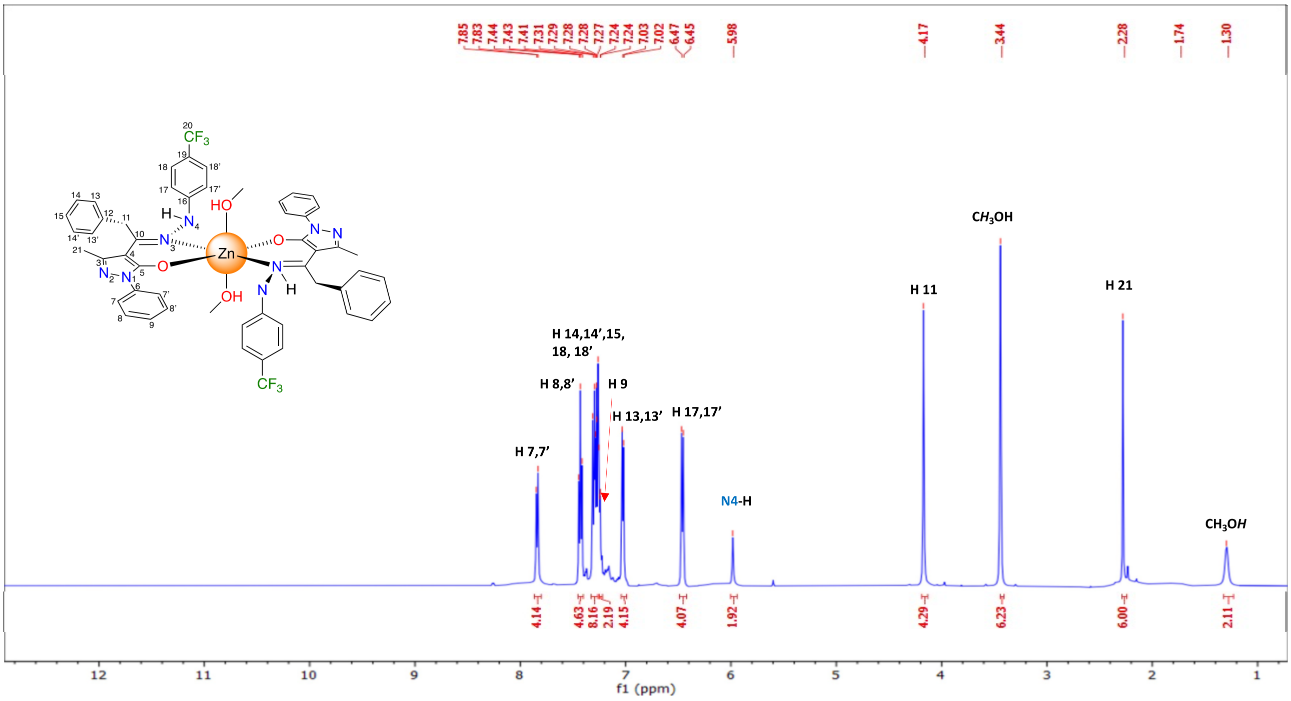

Figure S20. ^{1}H NMR spectrum in $CDCl_3$ at 298 K of [Zn(HL[1])$_2$(MeOH)$_2$] (**1**).

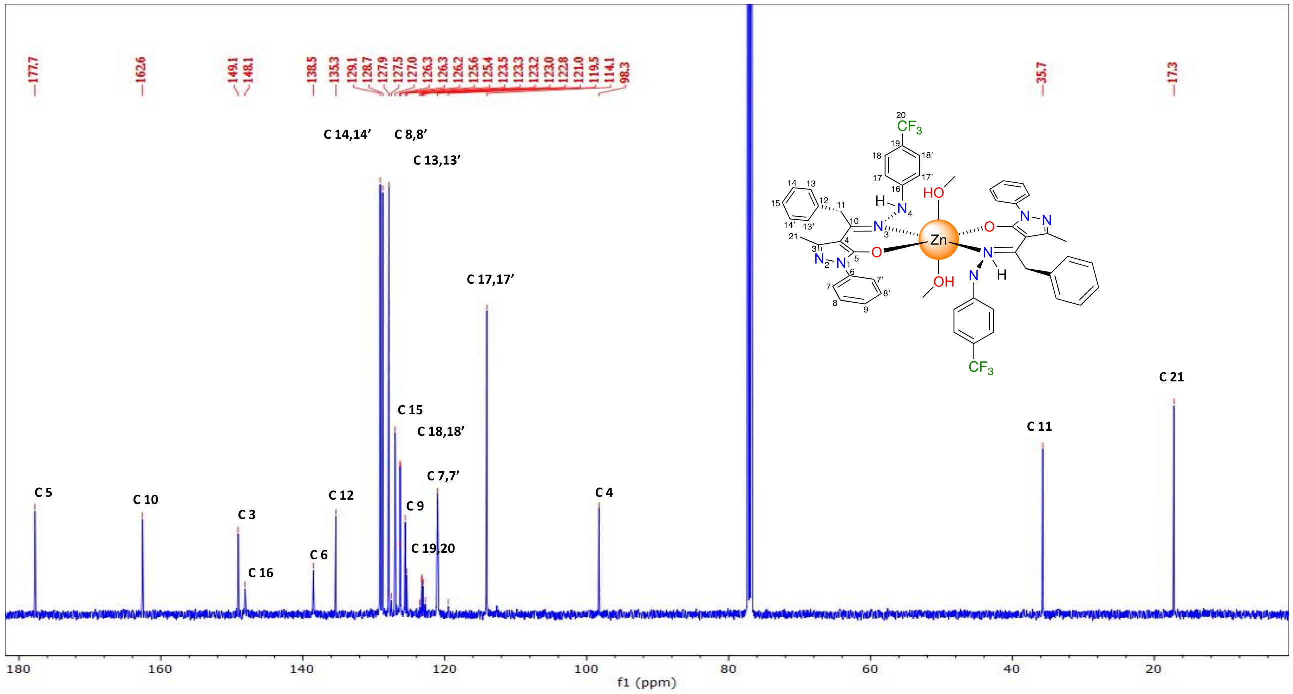

Figure S21. ^{13}C NMR spectrum in $CDCl_3$ at 298 K of $[Zn(HL^1)_2(MeOH)_2]$ (**1**).

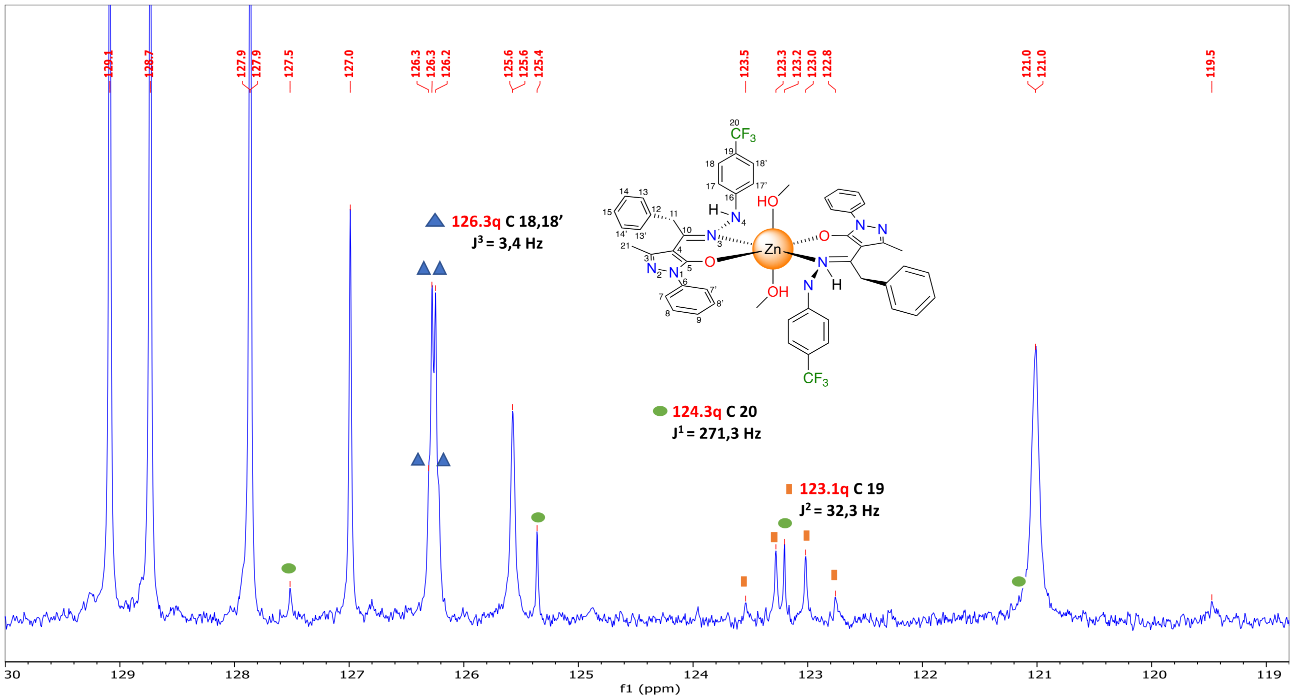

Figure S22. Magnification of ^{13}C NMR spectrum in $CDCl_3$ at 298 K of $[Zn(HL^1)_2(MeOH)_2]$ (**1**).

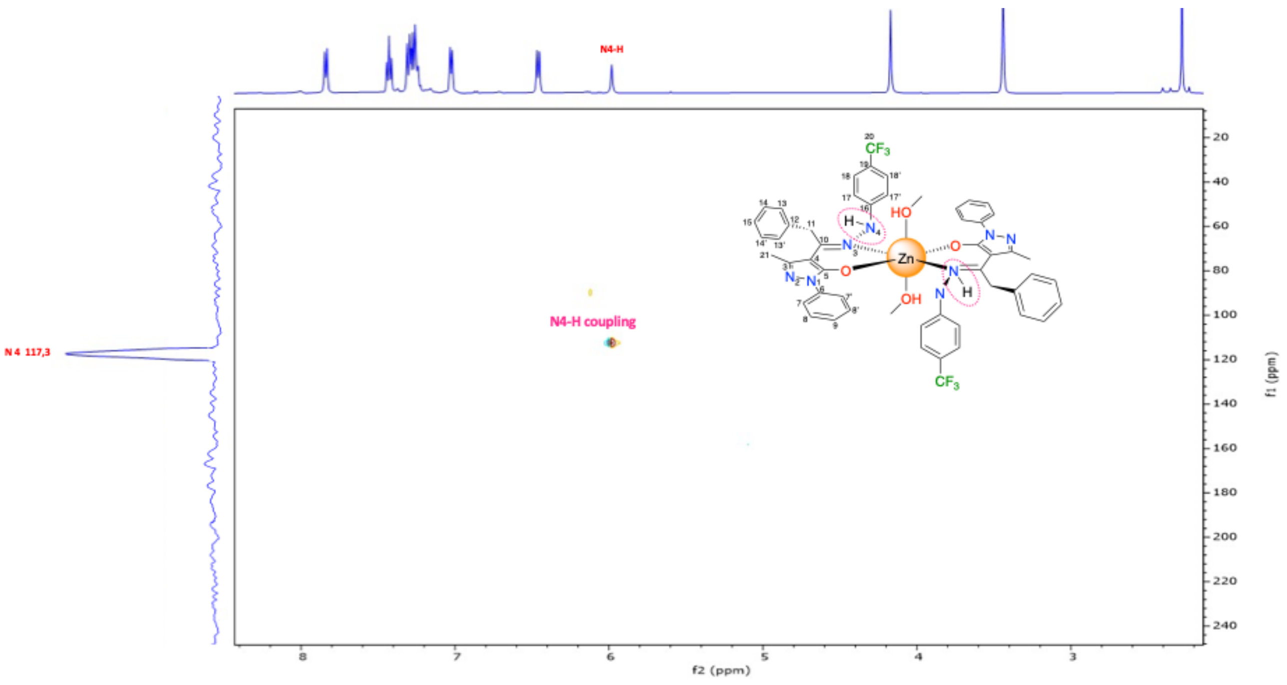

Figure S23. {^{1}H,^{15}N}-HSQC spectrum in $CDCl_3$ at 298 K of [Zn(HL^{1})$_2$(MeOH)$_2$] (**1**).

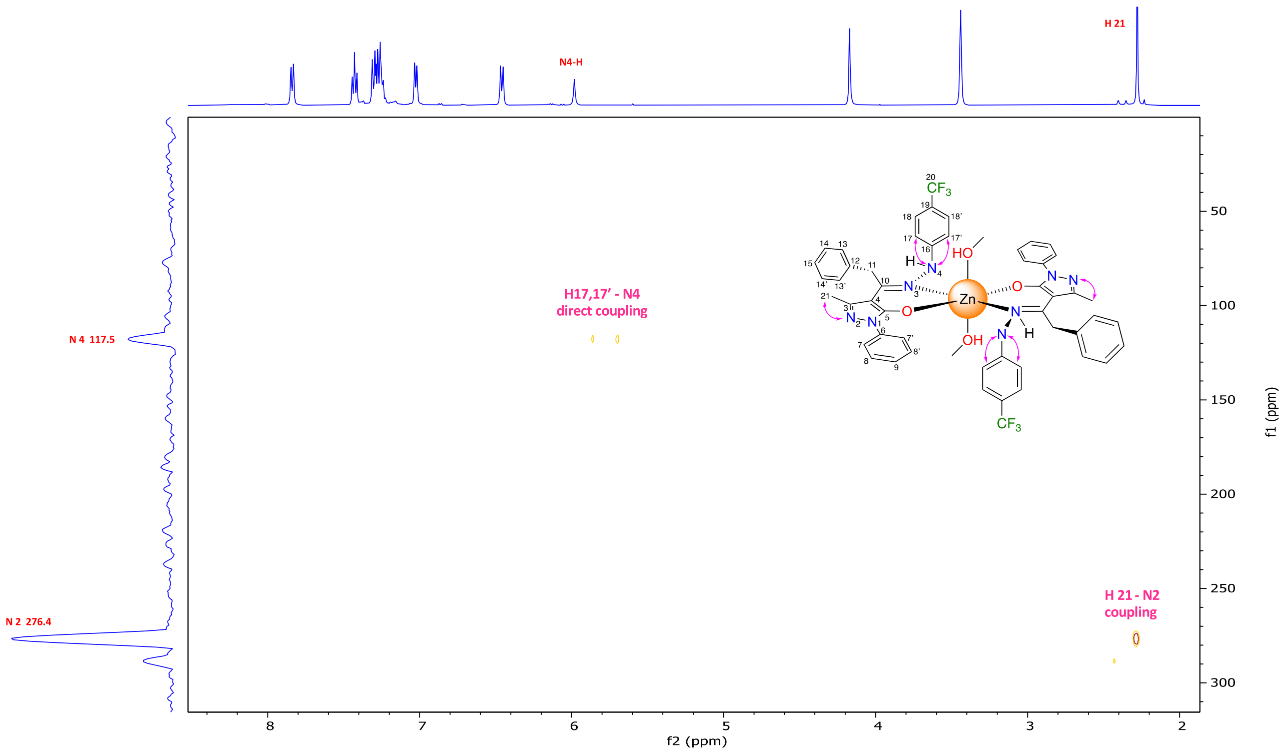

Figure S24. {^{1}H,^{15}N}-HMBC spectrum in $CDCl_3$ at 298 K of [Zn(HL^{1})$_2$(MeOH)$_2$] (**1**).

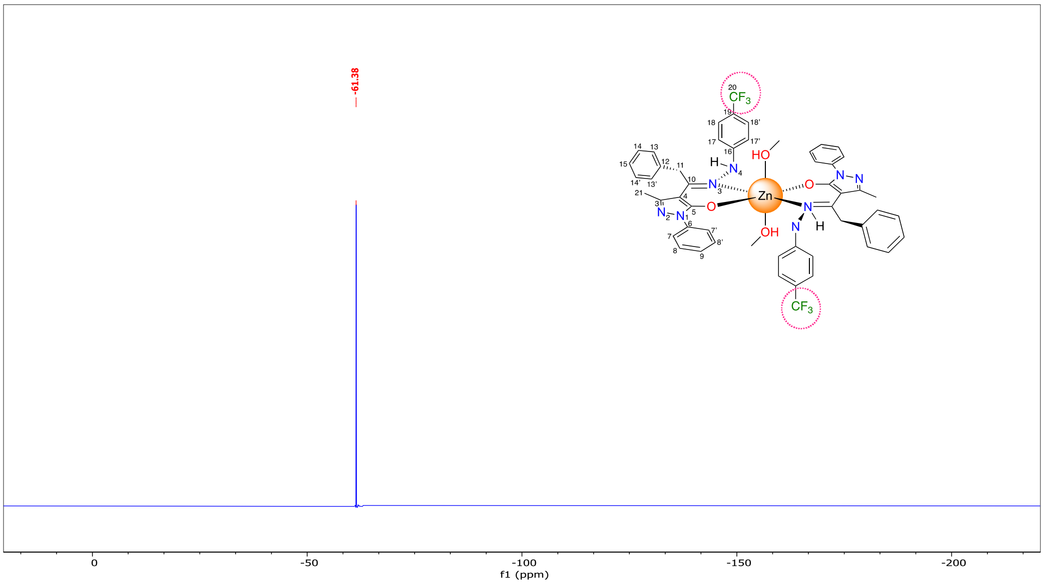

Figure S25. $^{19}F\{^{1}H\}$ NMR spectrum in $CDCl_3$ at 298 K of $[Zn(HL^1)_2(MeOH)_2]$ (**1**).

IR spectra of $[Zn(HL^1)_2(MeOH)_2]$ (1)

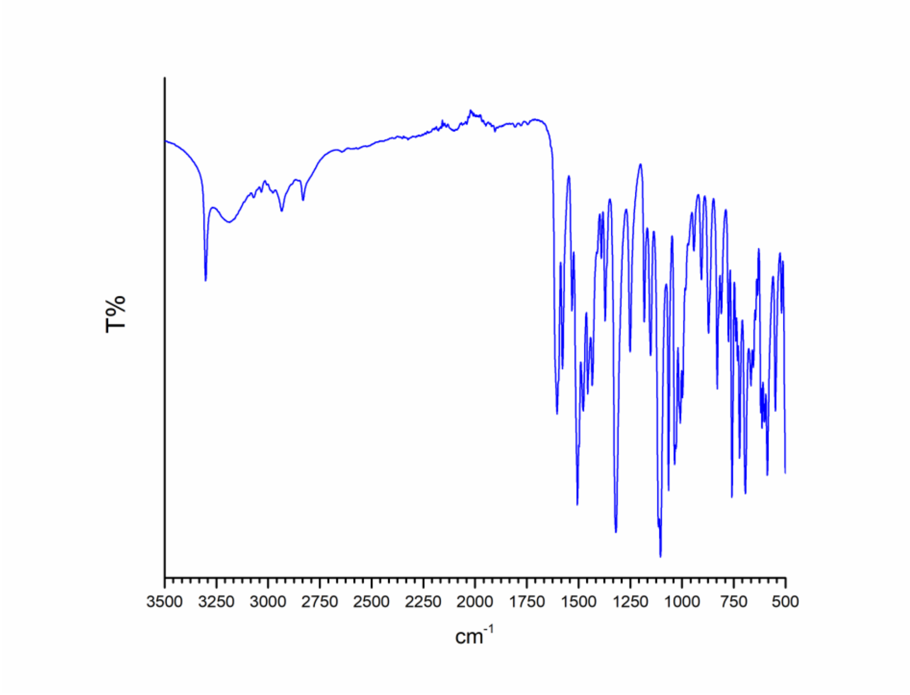

Figure S26. MIR spectrum of $[Zn(HL^1)_2(MeOH)_2]$ **(1).**

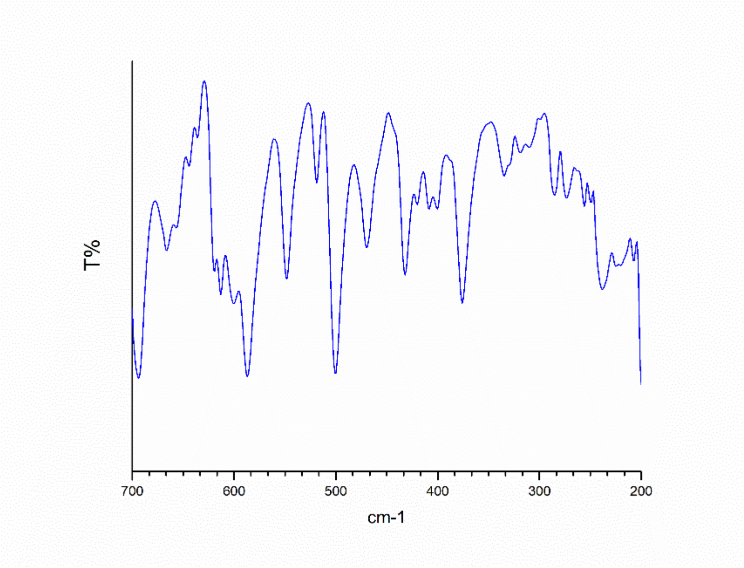

Figure S27. FIR spectrum of $[Zn(HL^1)_2(MeOH)_2]$ **(1).**

NMR spectra of $[Zn(HL^2)_2]$ (2)

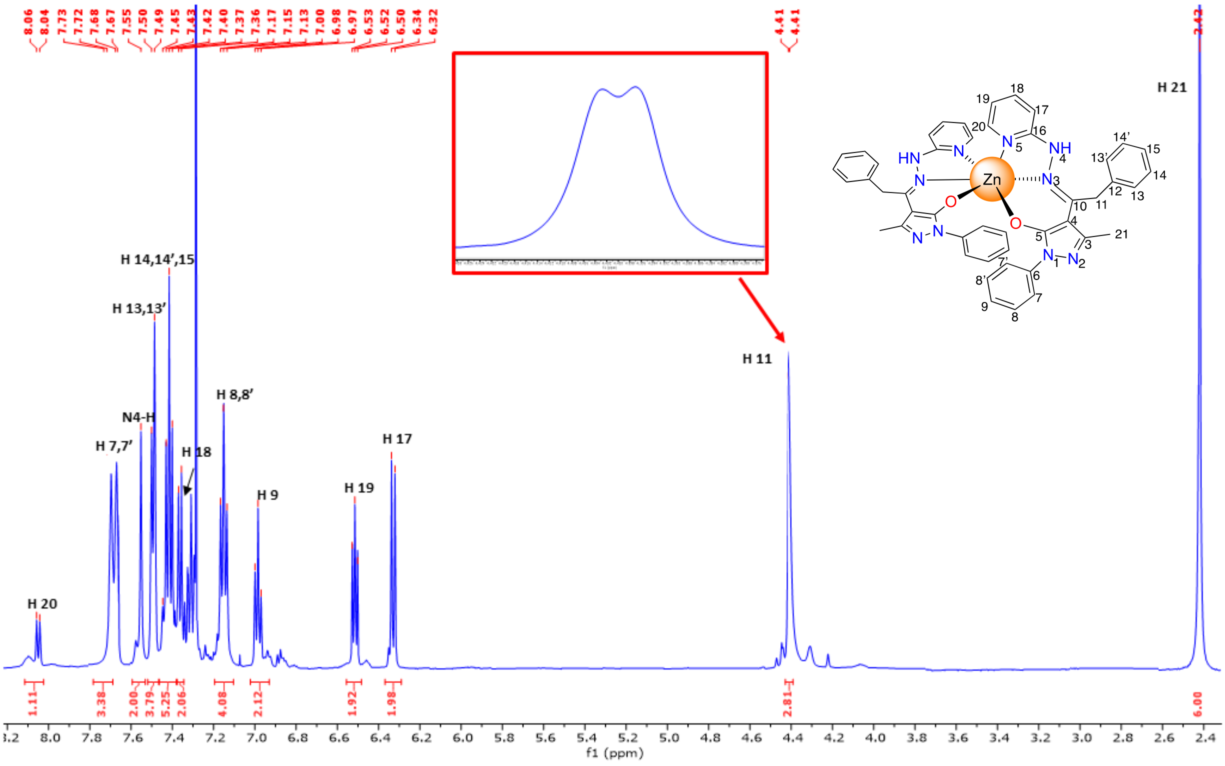

Figure S28. ^1H NMR spectrum in $CDCl_3$ at 298 K of $[Zn(HL^2)_2]$ (**2**).

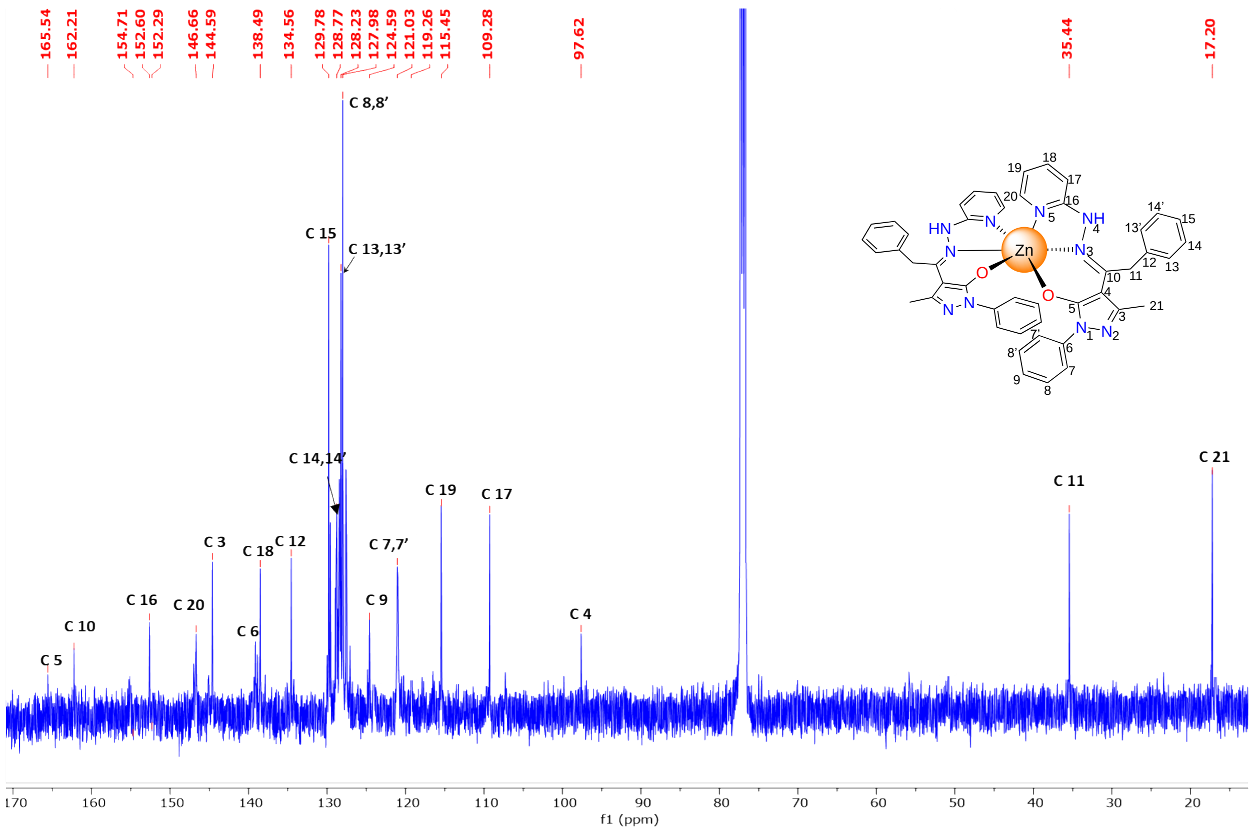

Figure S29. ^{13}C NMR spectrum in $CDCl_3$ at 298 K of $[Zn(HL^2)_2]$ (**2**).

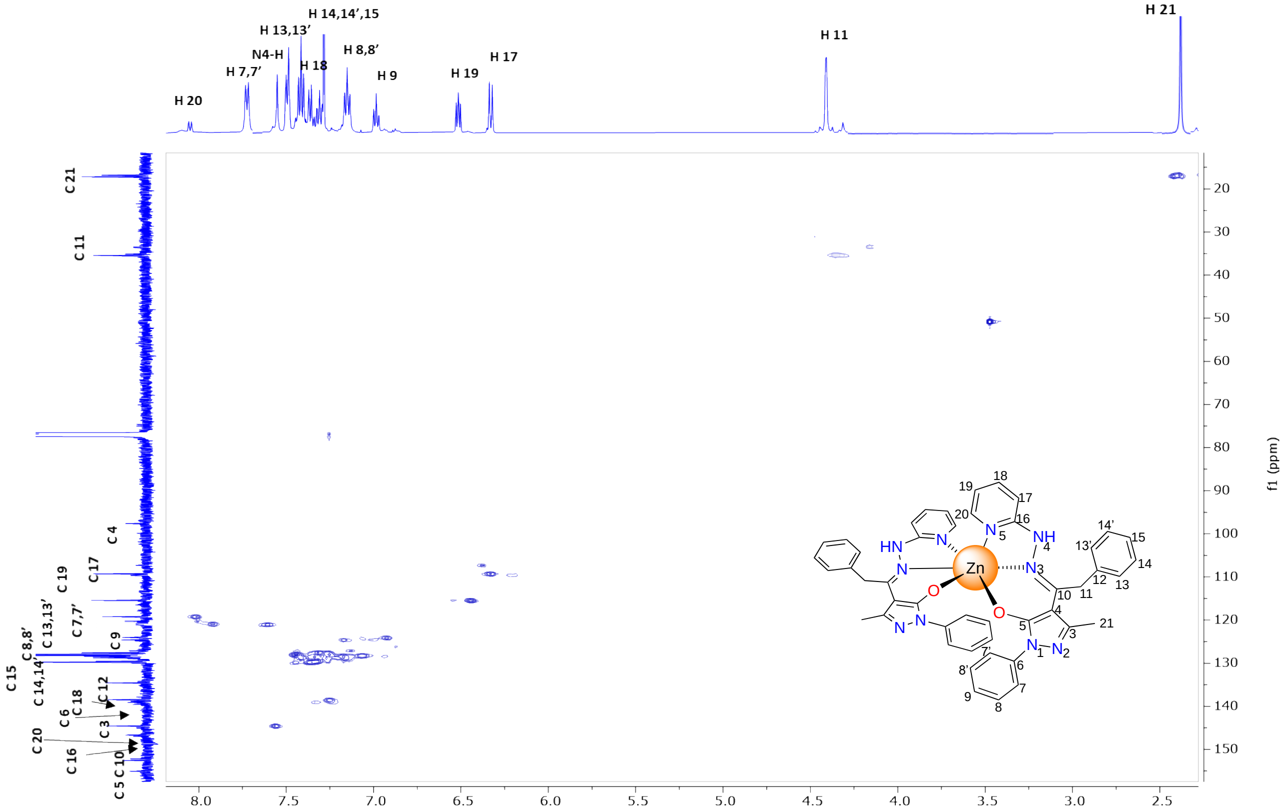

Figure S30. {^{1}H,^{13}C}-HSQC spectrum in $CDCl_3$ at 298 K of $[Zn(HL^2)_2]$ (**2**)

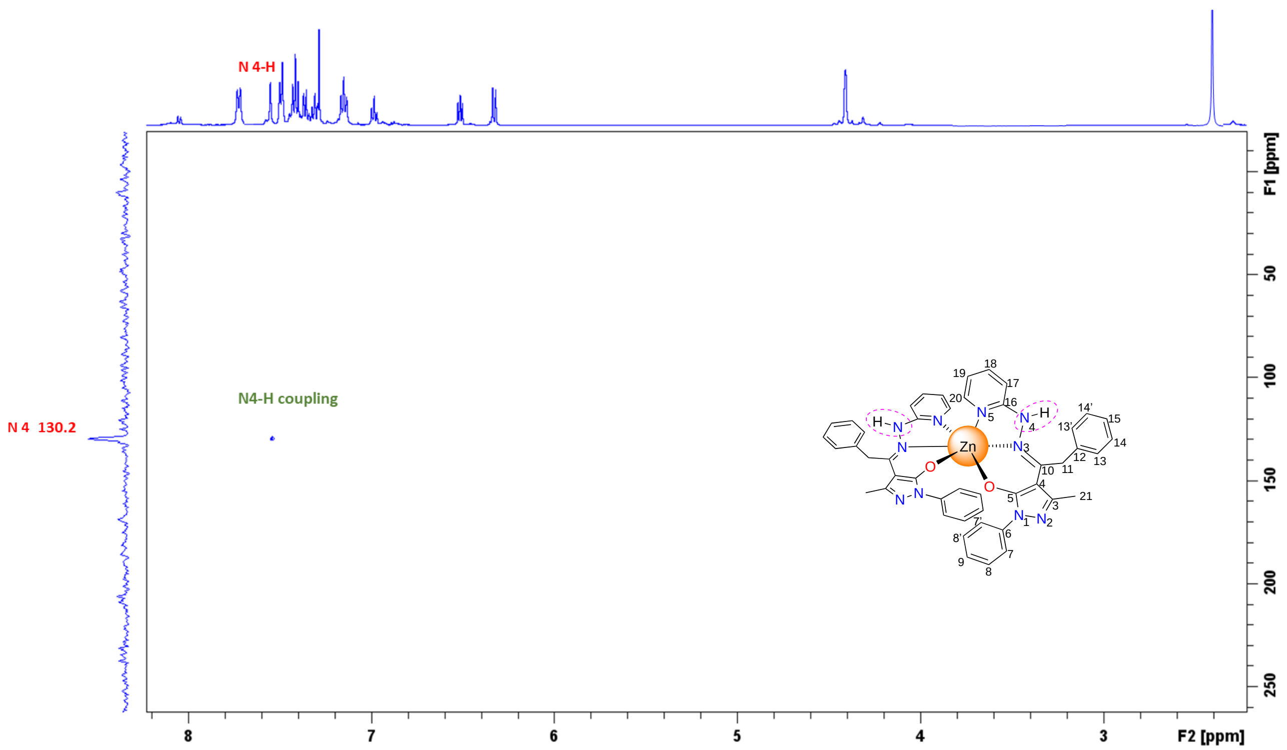

Figure S31. {^{1}H,^{15}N}-HSQC spectrum in $CDCl_3$ at 298 K of $[Zn(HL^2)_2]$ (**2**).

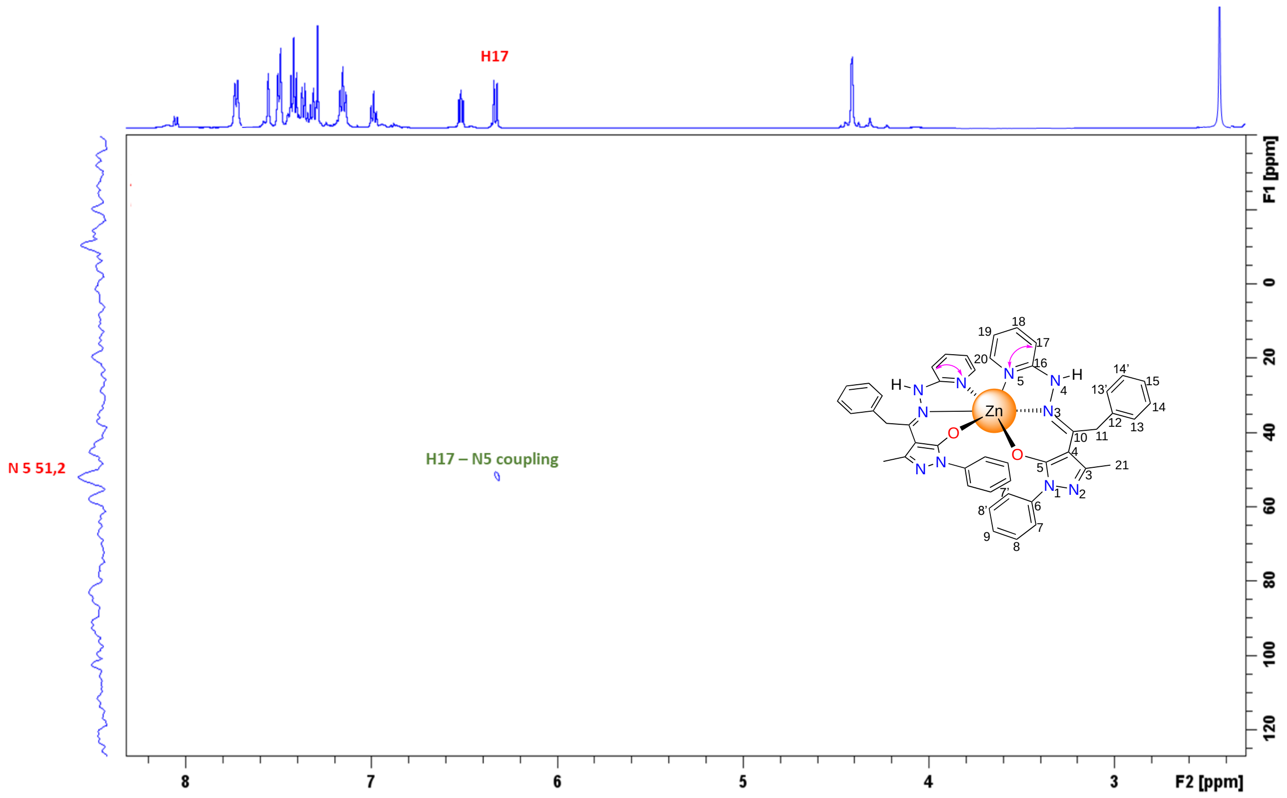

Figure S32. {^{1}H,^{15}N}-HMBC spectrum in $CDCl_3$ at 298 K of $[Zn(HL^2)_2]$ (**2**).

IR spectra of $[Zn(HL^2)_2]$ (2)

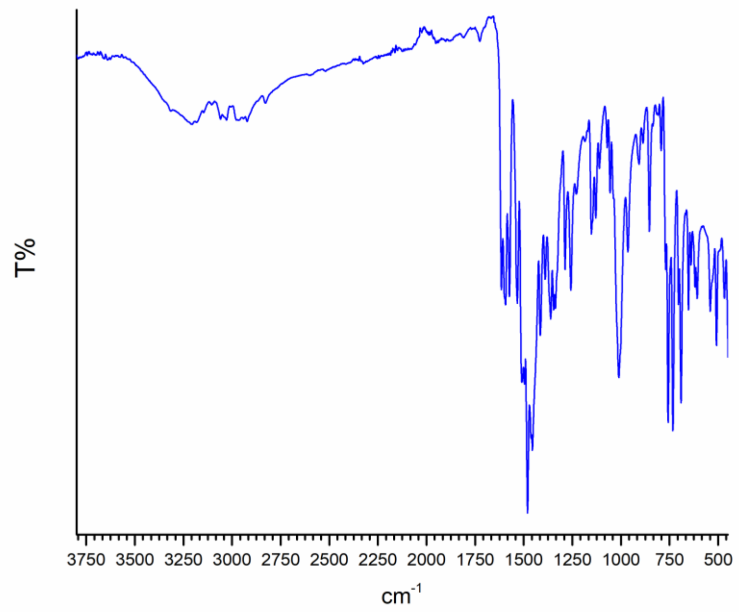

Figure S33. MIR spectrum of $[Zn(HL^2)_2]$ **(2)**.

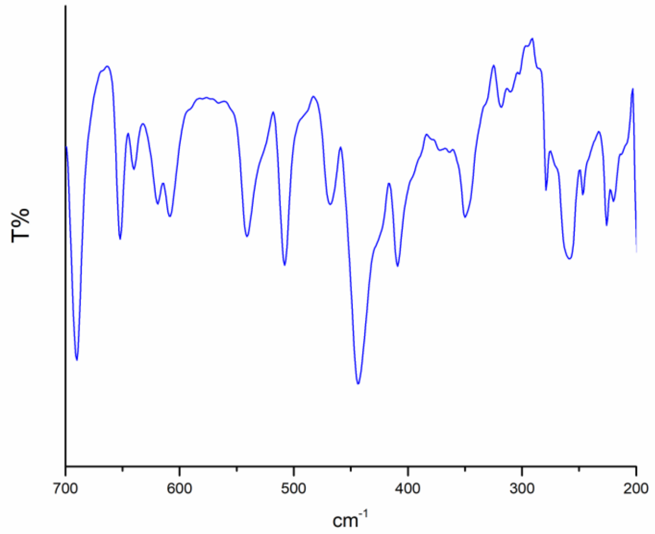

Figure S34. FIR spectrum of $[Zn(HL^2)_2]$ **(2)**.

IR spectra of [Cu(HL^1)$_2$] (3)

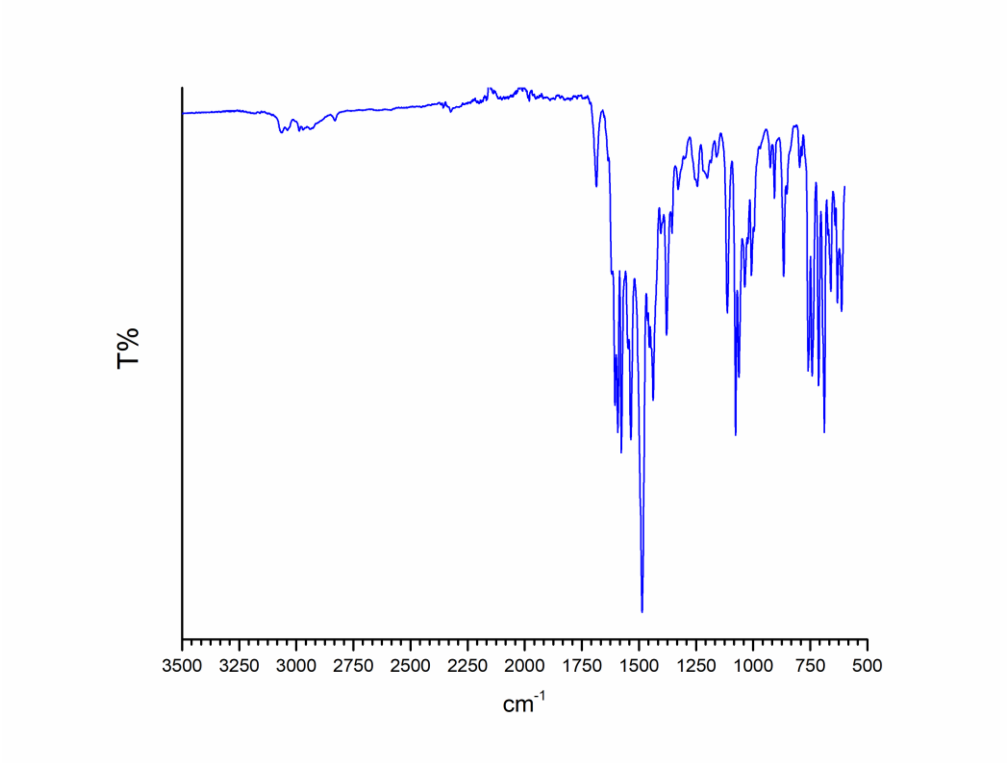

Figure S35. MIR spectrum of [Cu(HL^1)$_2$] **(3)**.

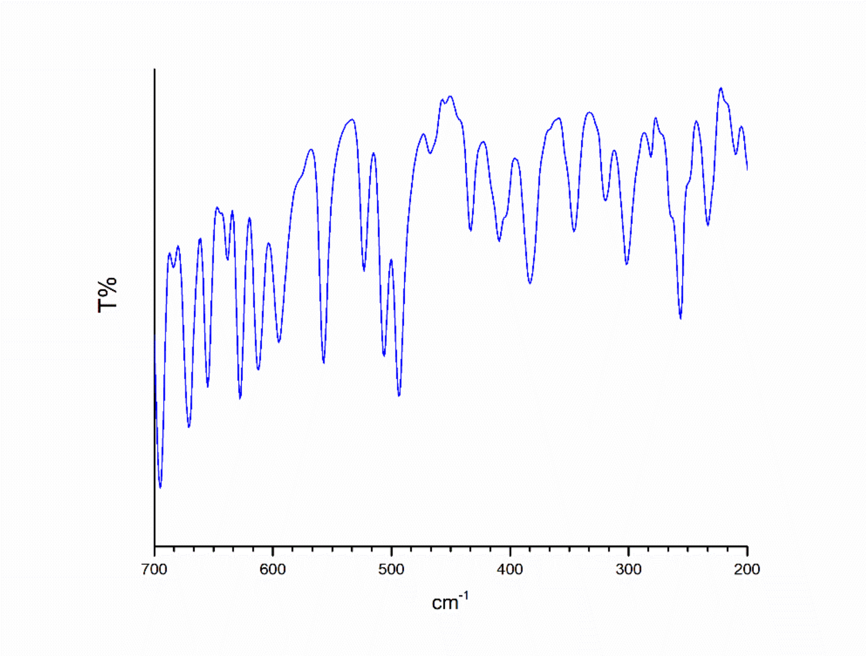

Figure S36. FIR spectrum of [Cu(HL^1)$_2$] **(3)**.

IR spectra of [Cu(HL^2)$_2$] (4)

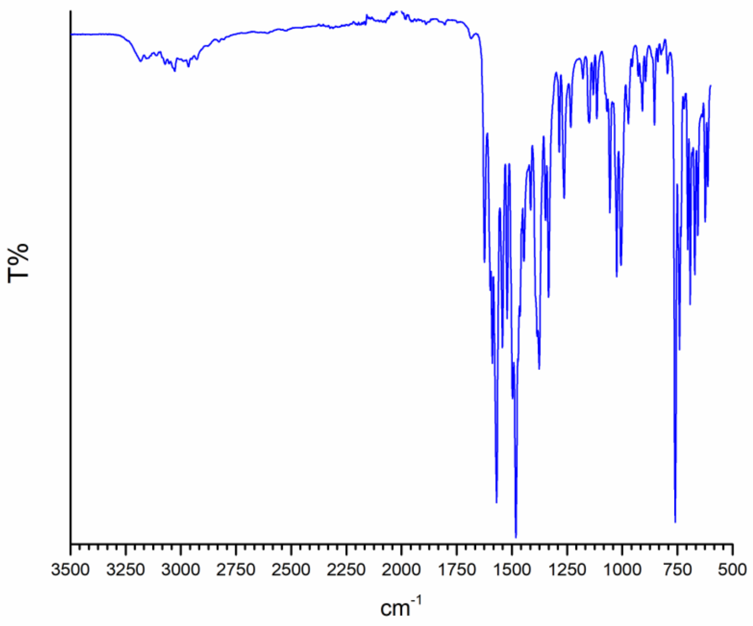

Figure S37. MIR spectrum of [Cu(HL^2)$_2$] **(4)**.

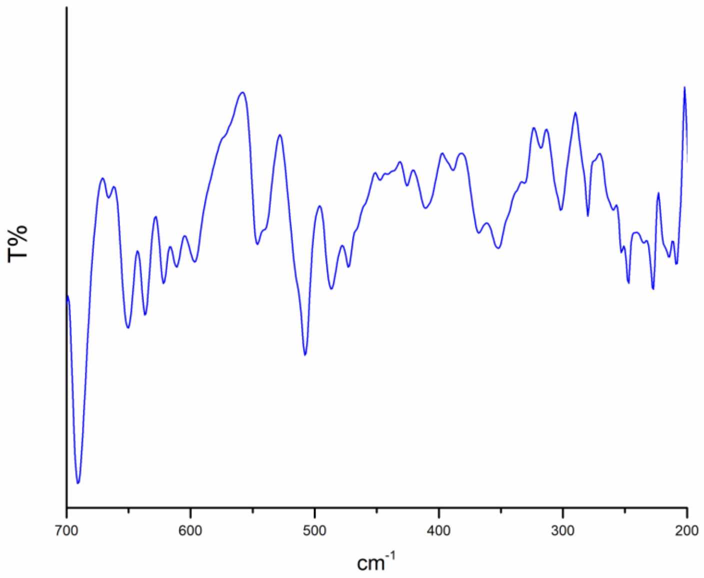

Figure S38. FIR spectrum of [Cu(HL^2)$_2$] **(4)**.

ESI-MS spectra

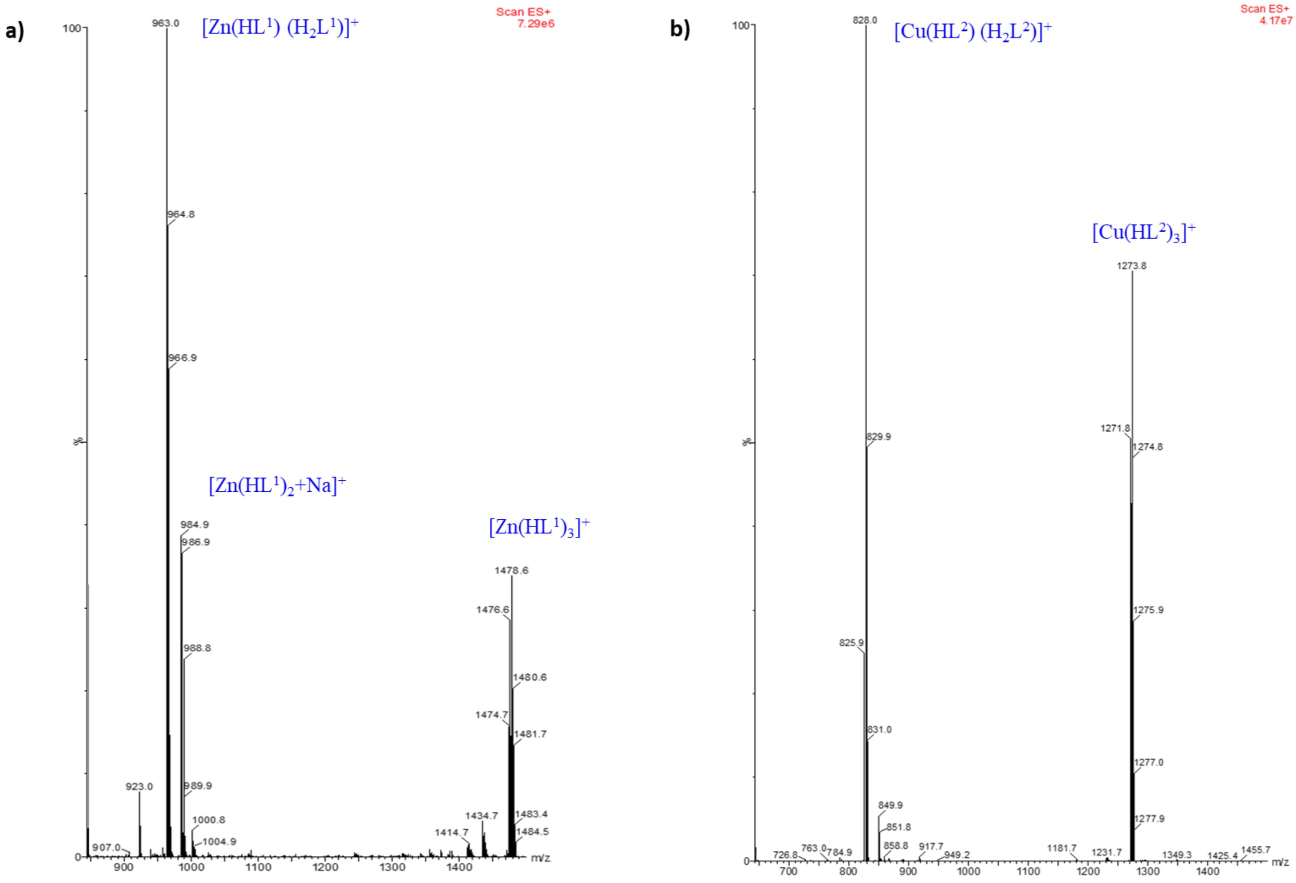

Figure S39. ESI-MS spectra of (a) $[Zn(HL^1)_2(MeOH)_2]$ **(1)** and (b) $[Cu(HL^2)_2]$ **(4)**.

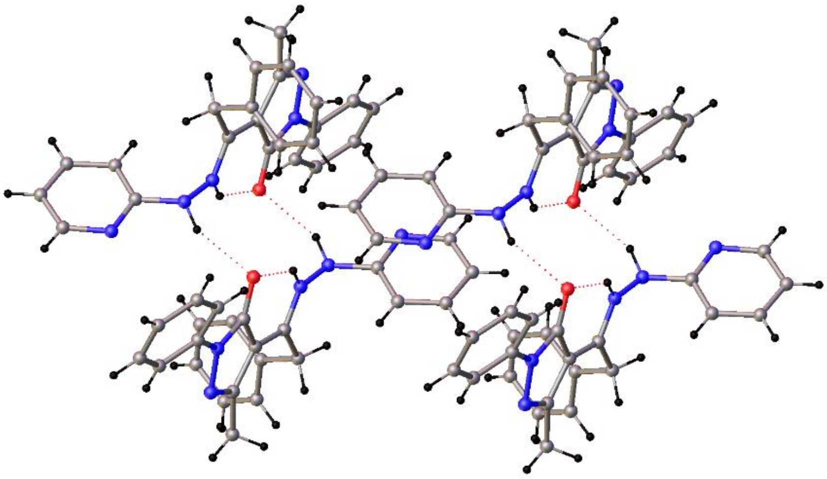

Figure S40. Crystal packing view of H_2L^2 showing N-H---O intramolecular and intermolecular hydrogen bonds [N(4)---O(1)^i and N(4)-H(4a)∠O(1) of 2.995(3) Å AND 135(3)° *i* = -*x*+1, -*y*+2, -*z*+2] and π–π interaction between the pyridine rings.

Table S1. Details of data collection and structure refinements for proligands **H_2L^1** and **H_2L^2**.

	**H_2L_1**	**H_2L_2**
formula	C_{25} H_{21} N_4 O F_3	C_{23} H_{21} N_5 O
Mr	450.46	383.45
crystal size [mm]	0.40 x 0.20 x 0.04	0.30 x 0.20 x 0.14
crystal system	Monoclinic	Triclinic
space group	$P2_1/c$	*P*-1
a [Å]	11.153(3)	8.9495(4)
b [Å]	22.940(6)	9.9398(5)
c [Å]	9.084(3)	12.5083(6)
α [°]	90	112.787(2)
β [°]	97.324(16)	91.215(2)
γ [°]	90	102.879(2)
V [$Å^3$]	2305.1(11)	992.86(8)
Z	4	2
ρ cald [gcm^{-3}]	1.295	1.283
μ [mm^{-1}]	0.098	0.082
θ range [°]	2.429 to 24.974	2.269 to 25.016
data collected	33167	10490
unique data, R_{int}	4001, 0.0758	3474, 0.0267
obs. data [$I > 2\sigma(I)$]	2155	2525
no. Parameters	326	259
restraints	0	48
R_1 [I>2sigma(I)]	0.0543	0.0627
wR_2 [all data]	0.1455	0.1784
GOF	1.001	1.032

Table S2. Details of data collection and structure refinements for complexes **1** and **5**.

	**1**	**5**
formula	$C_{54} H_{56} F_6 N_8 O_6 Zn$	$C_{36} H_{30} N_4 O_4 Cu$
Mr	1092.43	646.18
crystal size [mm]	0.40 x 0.20 x 0.06	0.40 x 0.20 x 0.10
crystal system	Triclinic	Triclinic
space group	*P*-1	*P*-1
a [Å]	9.941(3)	6.4458(2)
b [Å]	11.872(4)	9.3271(4)
c [Å]	12.109(5)	13.4708(5)
α [°]	95.200(17)	110.043(2)
β [°]	101.884(17)	96.231(2)
γ [°]	105.333(16)	95.695(2)
V [Å^3]	1332.6(8)	748.21(5)
Z	1	1
ρ cald [gcm^{-3}]	1.361	1.434
μ [mm^{-1}]	0.539	0.778
θ range [°]	2.303 to 25.678	3.258 to 26.372
data collected	11965	15703
unique data, R_{int}	5012, 0.0642	3045, 0.0260
obs. data [$I > 2\sigma(I)$]	2600	2627
no. Parameters	362	206
restraints	0	0
R_1 [I>2sigma(I)]	0.0543	0.0341
wR_2 [all data]	0.1314	0.0931
GOF	0.942	1.057

Table S3. Energies for the tautomer of proligands $\mathbf{H_2L^1}$ and $\mathbf{H_2L^2}$.

	$\mathbf{H_2L^1}$		$\mathbf{H_2L^2}$	
	Tautomer I	Tautomer II	Tautomer I	Tautomer II
	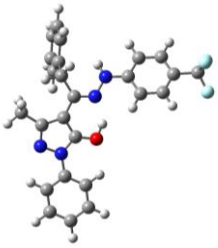	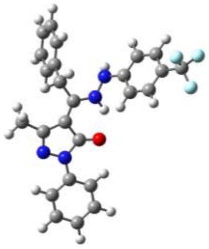	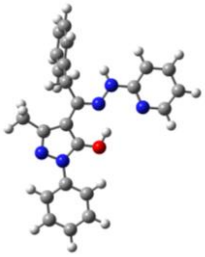	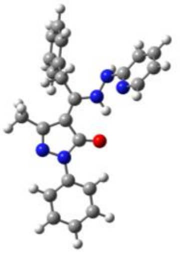
E	-1559,445250	-1559,449708	-1238,350939	-1238,354998
E_o	-1559,028991	-1559,032877	-1237,951191	-1237,954410
E_t	-1559,000852	-1559,004778	-1237,926833	-1237,930083
H	-1558,999908	-1559,003833	-1237,925889	-1237,929139
G	-1559,093531	-1559,097074	-1238,00875	-1238,012944
ΔE	0,0	-2,8	0,0	-2,5
ΔG	0,0	-2,2	0,0	-2,6
$\Delta E(CHCl_3)$	0,0	-4,4	0,0	-4,2
$\Delta G(CHCl_3)$	0,0	-3,8	0,0	-4,3

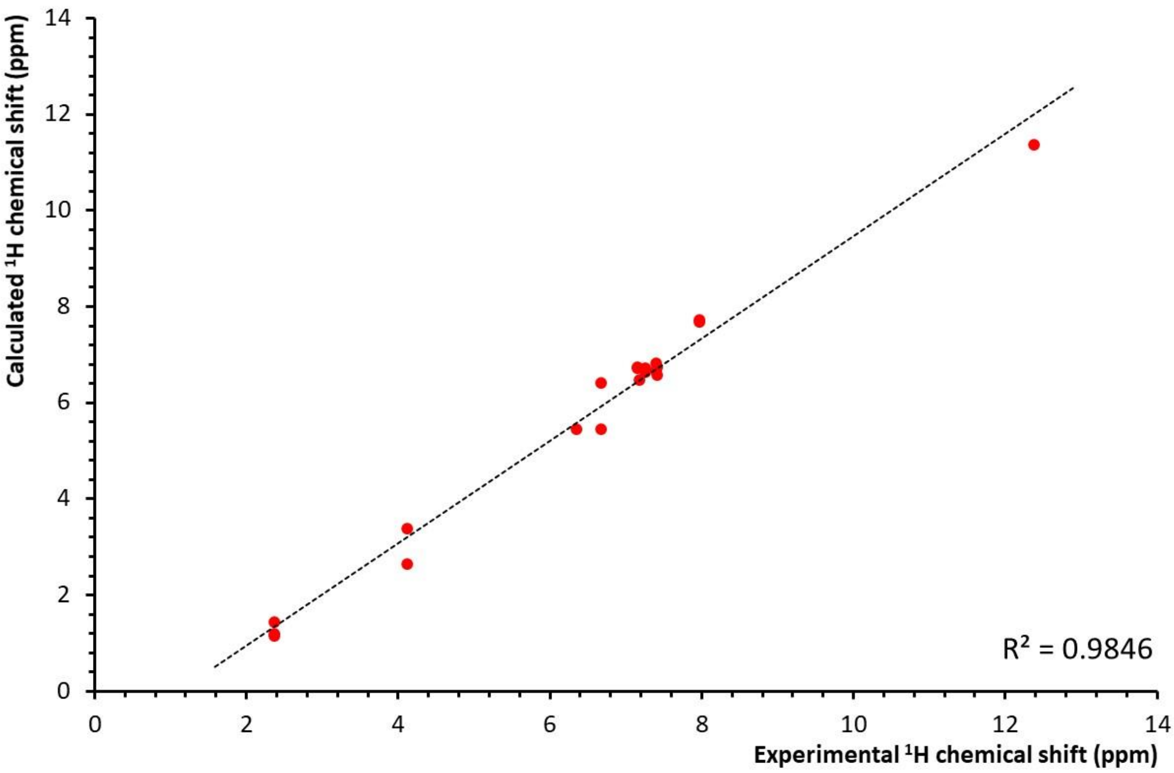

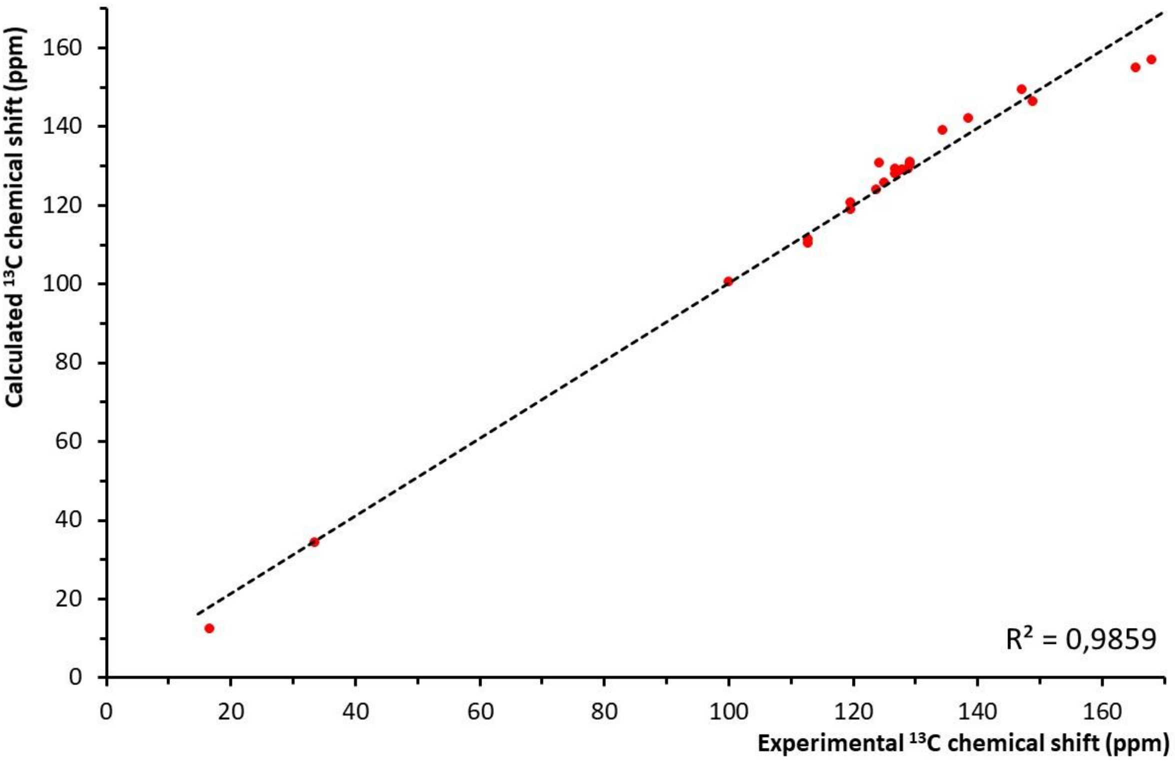

Figure S41. Comparison of the calculated (tautomer I) and experimental ^{1}H and ^{13}C NMR spectra of $\mathbf{H_2L^1}$.

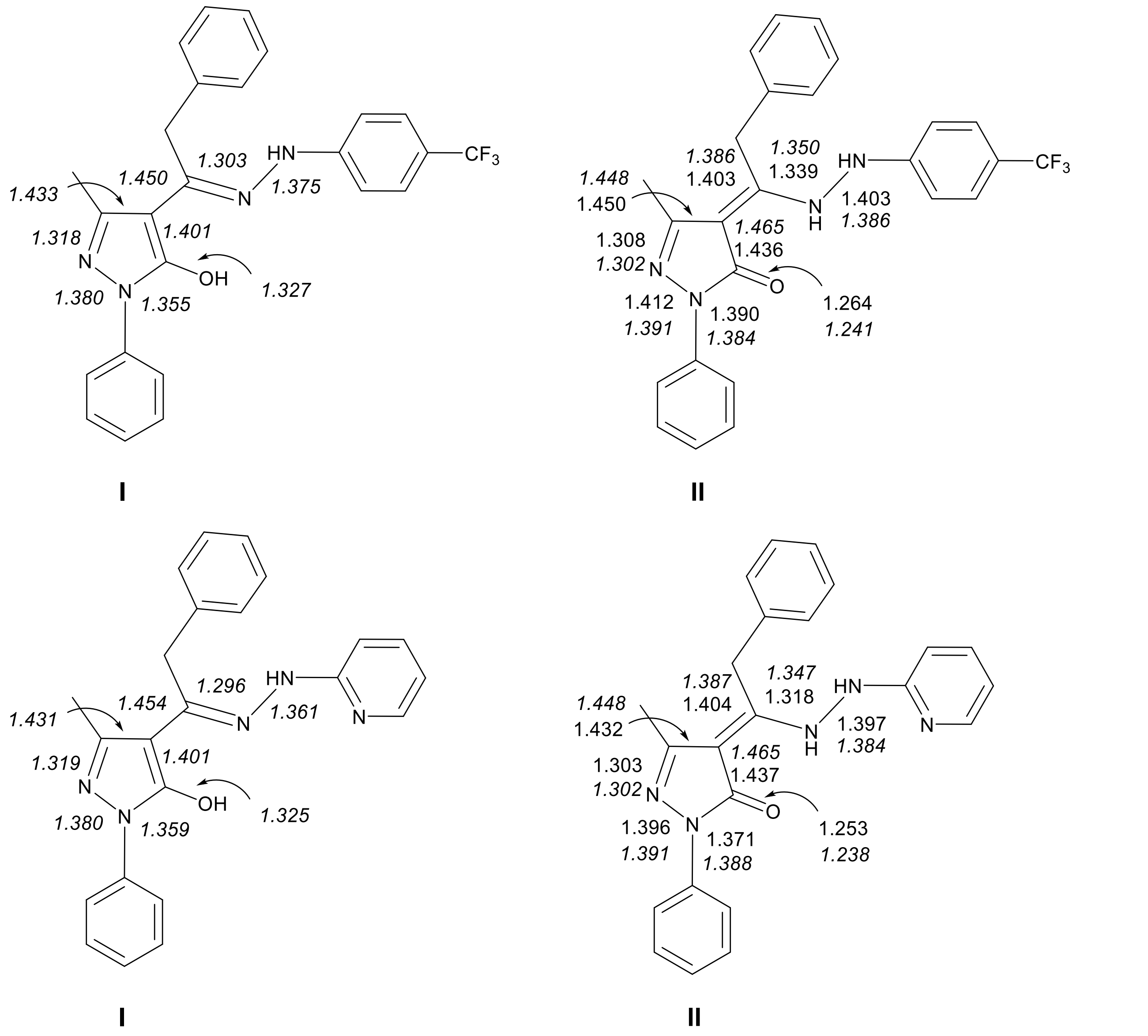

Figure S42. Comparison of selected bond distances of $\mathbf{H_2L^1}$ and $\mathbf{H_2L^2}$: calculated tautomers I and II (*italic*) and experimental.

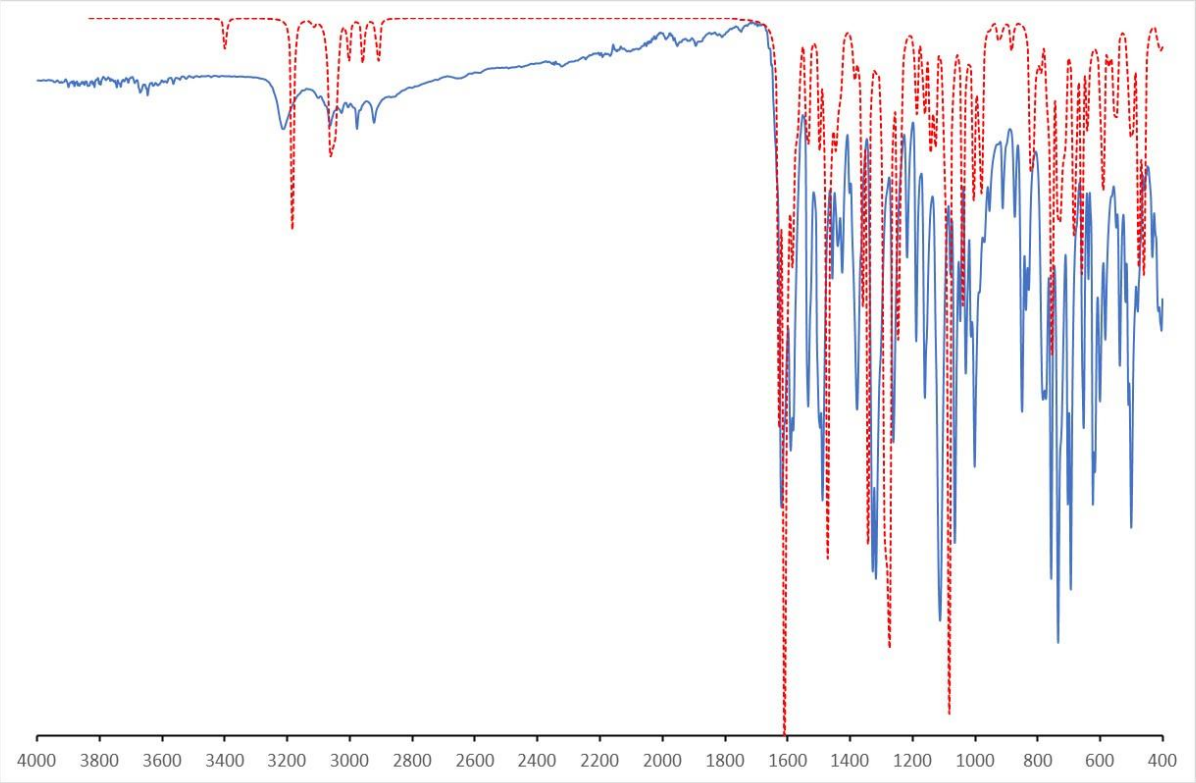

Figure S43. Comparison of the experimental (blue line) and calculated (tautomer II, red line) IR spectrum of $\mathbf{H_2L^1}$ (cm^{-1}).

Table S4. Selected stretching assignments of the IR spectrum of H_2L^1.

**IR band (cm^{-1})**	**Experimental**	**Calculated**
ν(N-H)	3211	3181
ν(C=O)	1620	1626
$\nu(C{=}N)_{pyrazole}$	1534	1537
ν_s(C-F)	1320	1278
ν(N-N)	1064	1141, 1128
ν_{as}(C-F)	1100	1086

Optimized [HL^1]$^-$

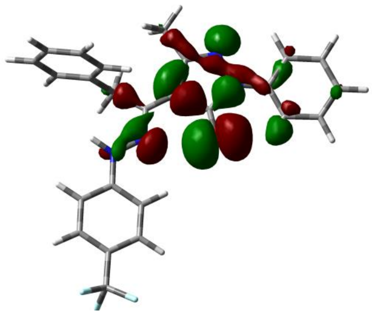
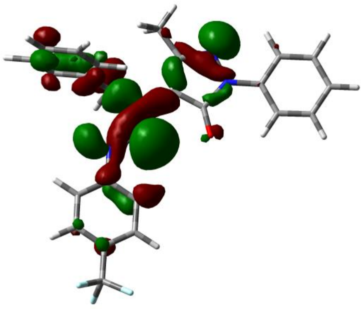
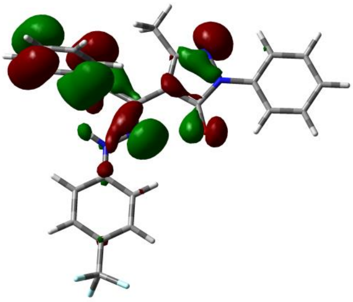

HOMO-2 HOMO-5 HOMO-9

Single-point calculation of [HL^1]$^-$ from the optimization of ***1***

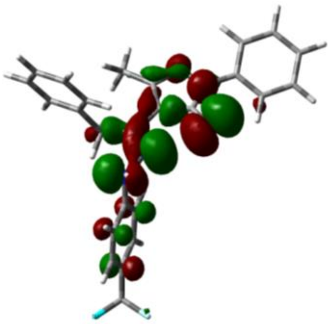
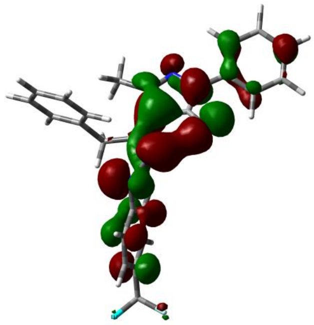

HOMO-1 HOMO-3

Single-point calculation of [HL^2]$^-$ from the optimization of ***2***

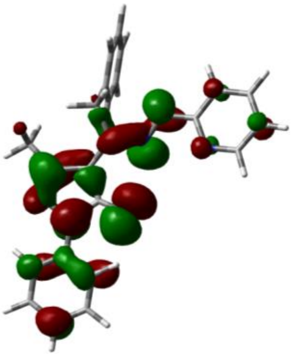
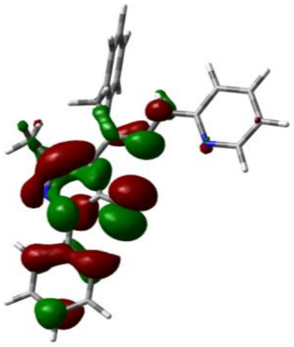
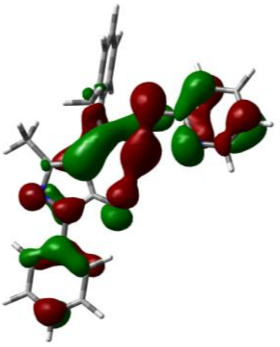

HOMO-1 HOMO-2 HOMO-3

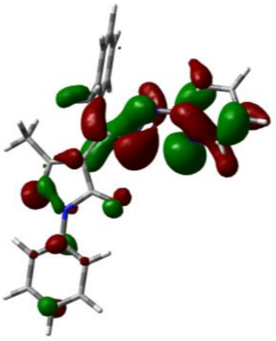
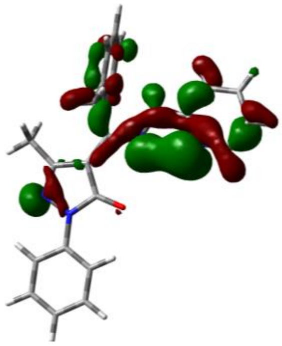

HOMO-4 HOMO-6

Figure S44. MOs of anionic ligands [HL^1]$^-$ and [HL^2]$^-$ involved in the coordination to metal centers.

Table S5. Comparison of selected experimental and calculated structural parameters of complex **1**.

**Bond distances (Å) and angles (º)**	**Experimental**	**Calculated**
Zn-O	1.989	2.050
Zn-N	2.116	2.088
Zn-O_{MeOH}	2.285	2.270
C=O	1.277	1.279
C-N	1.303	1.315
N-N	1.444	1.424
C-C	1.445	1.432
C-$C_{pyrazol}$	1.430	1.430
O-Zn-O	180.0	180.0
N-Zn-N	180.0	180.0
O_{MeOH}-Zn-O_{MeOH}	180.0	180.0

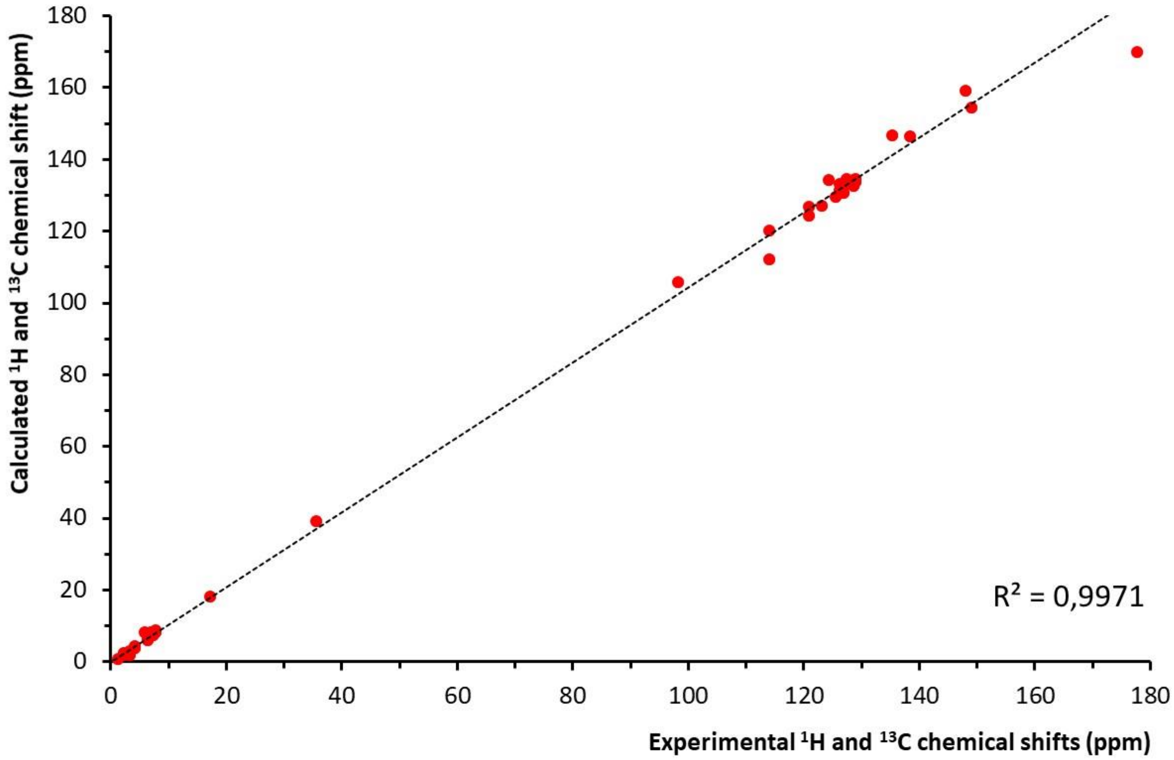

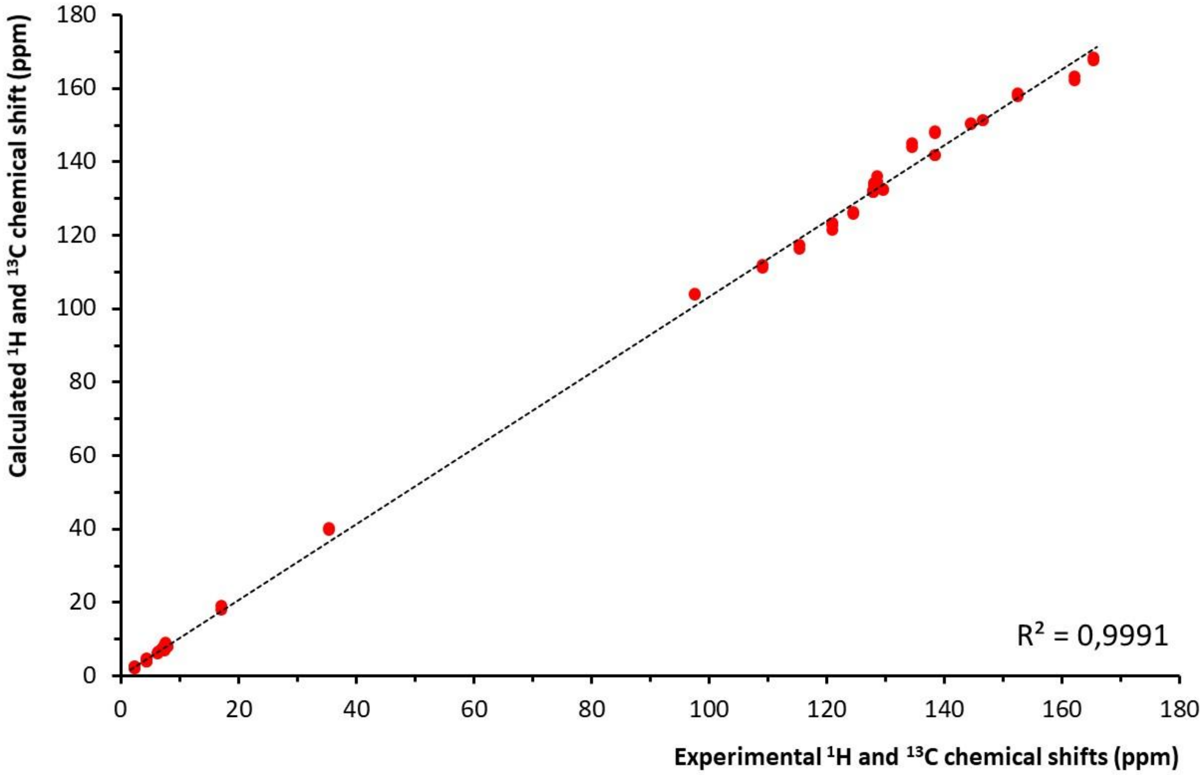

Figure S45. Comparison of the calculated and experimental ^{1}H and ^{13}C NMR spectra of complexes **1** (top) and **2** (bottom).

[Zn(HL[1])2]

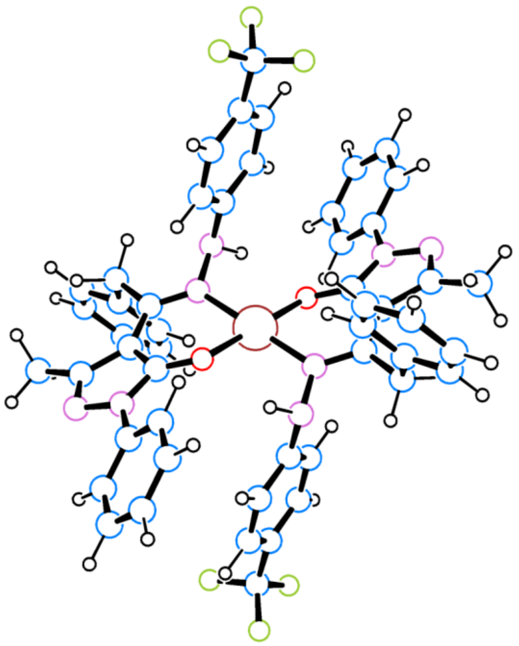

[Zn(HL[3])2]

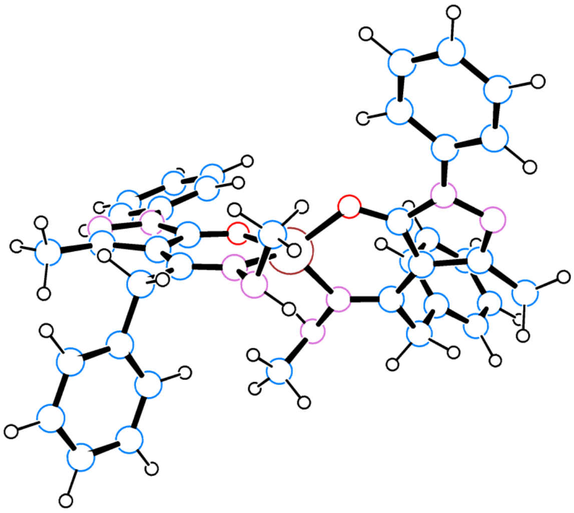

Tetrahedral

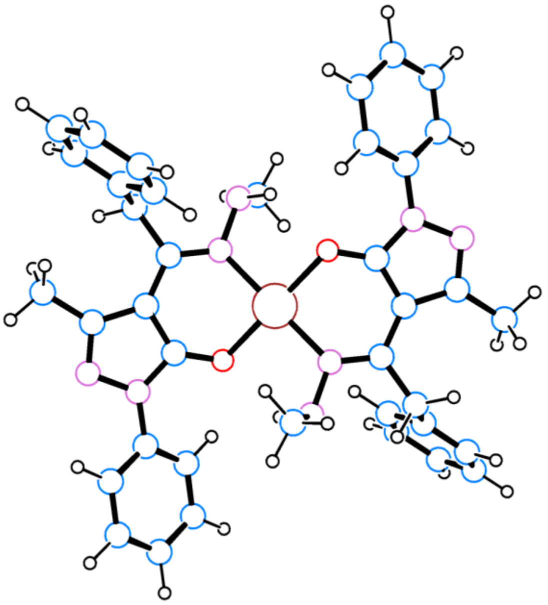

Square planar

Figure S46. Optimized structures of complexes [Zn(HL[1])2] and [Zn(HL[3])2].

Table S6. Comparison of selected experimental and calculated structural parameters of complex **5**.

**Bond distances (Å) and angles (º)**	**Experimental**	**Calculated**
Cu-O	1.931<br>1.893	1.936<br>1.919
C=O	1.268<br>1.276	1.271<br>1.272
C-N	1.354	1.367
N-N	1.401	1.392
C-C	1.404	1.406
C-$C_{pyrazol}$	1.420	1.435
O-Cu-O	180.0	180.0

Table S7. Coordinates of the optimized compounds.

H_2L^1 (tautomer I)

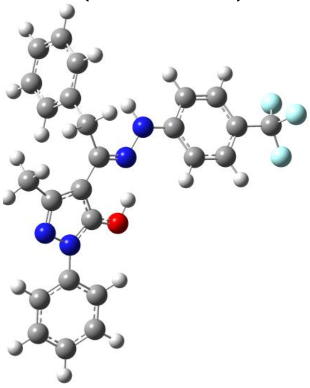

O	1.44690500	-1.49203800	0.14204800
H	0.58384600	-0.99706700	0.06064500
N	-0.16637100	0.48500500	-0.48954500
C	2.14905500	0.69430700	-0.71218700
C	2.35833800	-0.61334800	-0.25425600
C	0.82163900	1.26872700	-0.81791800
C	3.47488100	1.17968700	-0.96000900
C	3.94761800	2.50698300	-1.47046900
H	5.03779000	2.50955400	-1.47312000
H	3.60250500	3.33413900	-0.84372500
H	3.60582700	2.69423500	-2.49293100
N	3.69257400	-0.85187000	-0.24565800
N	4.38000800	0.26239900	-0.68311400
C	4.41906700	-2.01048900	0.15039400
C	3.78357000	-3.24261800	0.33412600
H	2.71961800	-3.33667400	0.18153800
C	5.79949200	-1.89727200	0.34527000
H	6.27219300	-0.93884000	0.18797900
C	6.53496800	-3.01409900	0.72375100
H	7.60466900	-2.91755700	0.87140200
C	4.53648000	-4.34894700	0.71904300
H	4.03695800	-5.30067700	0.86030700
C	5.91037200	-4.24500400	0.91599500
H	6.48809500	-5.11218700	1.21387200
C	0.59197500	2.69046600	-1.28341900
H	-0.19309100	2.65986200	-2.04691500
H	1.49093200	3.06356400	-1.77324900
C	-0.79913500	4.63863500	-0.45262500
H	-1.26867600	4.67343600	-1.43091800
C	0.17348700	3.66697300	-0.18955500
C	0.76703400	3.64034600	1.07799600
H	1.51640200	2.89022800	1.30596900
C	-0.56637500	5.53039300	1.77847600
H	-0.85134200	6.24825400	2.53889900
C	-1.16615800	5.56470600	0.52199800
H	-1.92030900	6.31085900	0.29837500
C	0.39995000	4.56469200	2.05293400
H	0.86978700	4.52903000	3.02933600
N	-1.44629100	0.98586300	-0.52671600
C	-2.52070400	0.12574800	-0.33683800
C	-3.75765200	0.65958200	0.06387600
H	-3.84553900	1.72287900	0.26119700
C	-2.41951300	-1.25274600	-0.57864400
H	-1.47897600	-1.67315400	-0.90625100
C	-3.52893100	-2.06980500	-0.41220500
H	-3.43959000	-3.13346000	-0.59399500
C	-4.86020200	-0.16349900	0.21847700
H	-5.80506300	0.25820300	0.53793300
C	-4.75478200	-1.53738700	-0.01331500
C	-5.96306900	-2.41365200	0.10400700
F	-5.64094000	-3.69637500	0.38600400
F	-6.80865400	-1.99069500	1.07304400
F	-6.68812400	-2.44503000	-1.04353700
H	-1.56966900	1.93653000	-0.19651800

H_2L^1 (tautomer II)

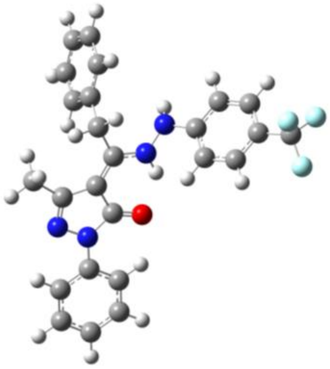

O	-1.63408600	-1.37303600	-0.88297100
N	0.21337200	0.46914800	-0.34625300
C	-1.92541700	0.52140700	0.61878800
C	-2.31752200	-0.68915300	-0.10427600
C	-0.66106800	1.07423500	0.48608000
C	-3.10074700	0.89504300	1.37794900
C	-3.32727200	2.03544800	2.32260200
H	-4.36549300	2.00913500	2.65391000
H	-3.13791200	3.00435200	1.85182700
H	-2.68589700	1.96137800	3.20655000
N	-3.63193600	-0.90713300	0.27154300
N	-4.07790900	0.06121700	1.16583400
C	-4.51700100	-1.94277100	-0.12139000
C	-4.11199500	-2.93760800	-1.02136100
H	-3.11086400	-2.91816900	-1.42336400
C	-5.81552100	-1.96469500	0.40324700
H	-6.11434300	-1.19191500	1.09607800
C	-6.69539300	-2.97350000	0.02832500
H	-7.69802500	-2.97971600	0.44158300
C	-5.00928500	-3.93872000	-1.38310800
H	-4.68638300	-4.70441800	-2.07986800
C	-6.30145800	-3.96710900	-0.86555500
H	-6.99180400	-4.75144500	-1.15374000
C	-0.19780800	2.32736700	1.18376800
H	0.79428800	2.13732900	1.59889600
H	-0.86550200	2.53319100	2.01886600
C	0.98443900	4.40087700	0.33994800
H	1.80019900	4.16566600	1.01558000
C	-0.12138100	3.54787100	0.27270600
C	-1.15865600	3.86224500	-0.61163000
H	-2.02110500	3.20879700	-0.68666900
C	0.01024800	5.85274100	-1.32548100
H	0.06038500	6.74295400	-1.94189200
C	1.04938400	5.54683200	-0.44942300
H	1.91333400	6.19846400	-0.38108000
C	-1.09245900	5.00538500	-1.40553300
H	-1.90520000	5.23329300	-2.08594600
N	1.49138600	0.96145800	-0.55578600
C	2.57692300	0.08877300	-0.38758800
C	3.81859000	0.45107200	-0.93255100
H	3.90856700	1.36717500	-1.50669900
C	2.46820200	-1.10073500	0.34204500
H	1.51211900	-1.40169500	0.74897300
C	3.58778900	-1.90226600	0.53051300
H	3.49445600	-2.82517800	1.08875700
C	4.92868900	-0.35335800	-0.73693600
H	5.88141800	-0.06908400	-1.16579100
C	4.82213500	-1.53698900	-0.00236000
C	6.03932400	-2.37348900	0.25437900
F	5.73333200	-3.67383500	0.45656400
F	6.91814000	-2.32183200	-0.77289600
F	6.71582100	-1.96134200	1.35586800
H	1.55316700	1.57209600	-1.36214700
H	-0.12918800	-0.36109400	-0.85219200

[HL[1]]-

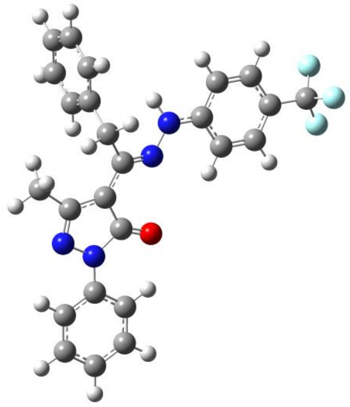

O	1.69119400	-1.76638200	-0.09512300
N	-0.26243600	0.38198000	-0.32614900
C	2.08627500	0.62981800	-0.48247300
C	2.39701200	-0.76358500	-0.21713800
C	0.74982200	1.13825500	-0.64655400
C	3.35098100	1.29879000	-0.52148200
C	3.69418900	2.74903000	-0.72786100
H	4.75145500	2.88171500	-0.49131400
H	3.10801200	3.41464000	-0.08748500
H	3.54314600	3.07113800	-1.76503700
N	3.82342600	-0.77280300	-0.13803400
N	4.36749300	0.48916600	-0.31292900
C	4.67874200	-1.85312900	0.09030600
C	4.18932800	-3.15813800	0.29407200
H	3.12174100	-3.31623300	0.27089700
C	6.07074100	-1.64169300	0.11817300
H	6.43649600	-0.63728600	-0.03888800
C	6.93931800	-2.70240300	0.34311600
H	8.00889800	-2.51306800	0.35988300
C	5.07864100	-4.20442800	0.51732100
H	4.67984700	-5.20276900	0.67203600
C	6.45693000	-3.99552000	0.54534400
H	7.13962000	-4.82062400	0.72033500
C	0.52405200	2.52805900	-1.23582000
H	-0.20940200	2.42860400	-2.04553900
H	1.44087900	2.87842400	-1.70715800
C	-0.70943100	4.68655700	-0.74025300
H	-0.95592700	4.74113100	-1.79678000
C	0.03393700	3.59669700	-0.26807600
C	0.33255400	3.54279700	1.09842700
H	0.89222200	2.69751300	1.48305600
C	-0.82749800	5.63129300	1.47792100
H	-1.16151000	6.41247200	2.15236400
C	-1.13579300	5.69534600	0.12022400
H	-1.71203000	6.52859600	-0.26879400
C	-0.09365500	4.55049800	1.96177500
H	0.14636000	4.48630900	3.01781300
N	-1.53391500	0.87702100	-0.54023900
C	-2.60396600	0.04492700	-0.33143600
C	-3.91552800	0.56872100	-0.32671100
H	-4.06512400	1.63648700	-0.45556300
C	-2.42564900	-1.34418400	-0.14940300
H	-1.41499200	-1.73102700	-0.13712200
C	-3.52840200	-2.16259100	0.02092300
H	-3.38019500	-3.22581900	0.16764500
C	-5.00601100	-0.26397200	-0.15692200
H	-6.00556400	0.15543400	-0.14842200
C	-4.82907000	-1.64196600	0.02085800
C	-6.01302800	-2.53223900	0.14279500
F	-5.72668500	-3.71656400	0.73814500
F	-7.02257000	-1.96807500	0.86290900
F	-6.57447400	-2.85267600	-1.06257600
H	-1.70885600	1.87205200	-0.44855200

H_2L^2 (tautomer I)

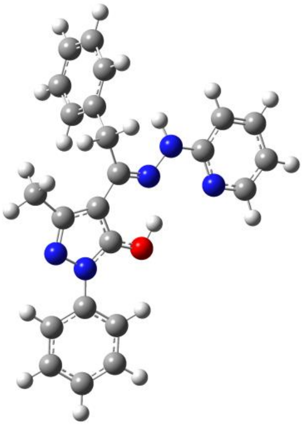

O	-1.48837200	1.31070700	-0.03951400
H	-0.50114000	1.44299600	-0.12678000
N	0.97487100	0.54886200	-0.48486800
C	-0.77161000	-0.96763800	-0.61955900
C	-1.70006000	0.02615900	-0.27692200
C	0.64322700	-0.67291100	-0.76765200
C	-1.56692600	-2.15192400	-0.73105100
C	-1.17362600	-3.55835300	-1.06899400
H	-2.04922900	-4.19943900	-0.96363700
H	-0.38835300	-3.93520500	-0.40742600
H	-0.81435900	-3.64488800	-2.09926700
N	-2.92770400	-0.55409900	-0.20927600
N	-2.83849200	-1.90323500	-0.48595100
C	-4.19403800	0.00695500	0.11444200
C	-4.39114500	1.39126300	0.16357100
H	-3.57406200	2.06503000	-0.04175800
C	-5.26026100	-0.86047100	0.37745300
H	-5.08973300	-1.92574800	0.32494600
C	-6.51119700	-0.34174500	0.68932400
H	-7.33054600	-1.02282900	0.89065500
C	-5.65078200	1.89175400	0.48323300
H	-5.79440400	2.96585400	0.51973600
C	-6.71580200	1.03561700	0.74744200
H	-7.69276800	1.43531000	0.99350300
C	1.64577200	-1.69889100	-1.25616500
H	2.16936000	-1.26493200	-2.11654900
H	1.11748400	-2.57459900	-1.63307000
C	4.00538900	-2.35969900	-0.60546000
H	4.30091700	-2.18782500	-1.63614400
C	2.67650200	-2.14133700	-0.22429400
C	2.31711100	-2.37287400	1.10856800
H	1.29509200	-2.19882900	1.42715200
C	4.58049400	-3.03262800	1.64045900
H	5.31406800	-3.37550600	2.36104600
C	4.95096000	-2.80249900	0.31797300
H	5.97526200	-2.96713700	0.00260600
C	3.26077500	-2.81533900	2.03201900
H	2.96397400	-2.98874000	3.06030100
N	2.26507600	0.96659100	-0.60181900
C	2.57372900	2.28503100	-0.28502000
C	3.92634500	2.66112300	-0.17170300
H	4.71537700	1.92820200	-0.29904400
C	1.87120700	4.41816900	0.13515400
H	1.02246100	5.08627600	0.25259600
C	4.21469200	3.98450000	0.10522600
H	5.24612100	4.30525100	0.20007600
C	3.16773800	4.89725200	0.26404000
H	3.00516100	0.28313500	-0.49150200
N	1.56514300	3.14438900	-0.13452900
H	3.35307400	5.94133100	0.48127300

H_2L^2 (tautomer II)

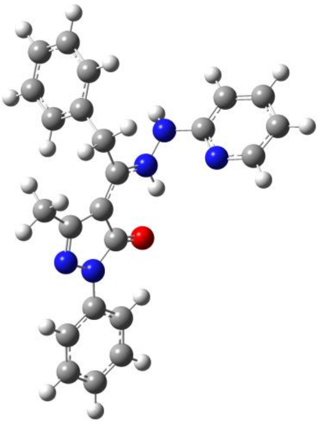

O	1.48580000	1.19295600	-0.93784300
N	-1.07473200	0.58864600	-0.42630000
C	0.71429800	-0.56709100	0.55975100
C	1.69209800	0.25728600	-0.15205600
C	-0.65171000	-0.37119700	0.41826700
C	1.50842600	-1.50232500	1.32802900
C	1.09145400	-2.59650700	2.26281000
H	1.98569500	-3.11789100	2.60497000
H	0.43083400	-3.32178300	1.77889700
H	0.57027000	-2.20200400	3.14080200
N	2.92254400	-0.24632800	0.24570000
N	2.78142400	-1.30632000	1.13536100
C	4.22324200	0.16892500	-0.12944600
C	4.41528700	1.24129000	-1.01160300
H	3.55862800	1.75993700	-1.41338600
C	5.33302500	-0.50704800	0.39460200
H	5.17089900	-1.33081500	1.07401800
C	6.61682800	-0.11145700	0.03738400
H	7.46691200	-0.64326900	0.45070100
C	5.70971100	1.62080800	-1.35586700
H	5.84709500	2.45223700	-2.03860700
C	6.81690600	0.95336900	-0.83886500
H	7.82028700	1.25778800	-1.11366900
C	-1.70666900	-1.18607200	1.12246200
H	-2.42465300	-0.49259900	1.56563800
H	-1.24089100	-1.73305300	1.94028700
C	-3.83907800	-2.29176500	0.32304500
H	-4.38172600	-1.66834200	1.02603900
C	-2.45139700	-2.15955300	0.21696800
C	-1.76960300	-2.96544700	-0.70094700
H	-0.69399800	-2.87190400	-0.80518200
C	-3.84216700	-4.01088500	-1.37228600
H	-4.37830900	-4.72603600	-1.98564900
C	-4.53027200	-3.21219900	-0.46155700
H	-5.60638600	-3.30245500	-0.36294900
C	-2.46006100	-3.88267700	-1.49041900
H	-1.91561200	-4.49815800	-2.19780200
N	-2.41620200	0.89338500	-0.58124200
C	-2.86280800	2.16748800	-0.20093500
C	-4.07924700	2.65014200	-0.71507400
H	-4.64933000	2.06818700	-1.43055700
C	-2.56606600	4.03366300	1.07878000
H	-1.92980200	4.55338600	1.78923800
C	-4.52252100	3.88539300	-0.27631900
H	-5.45586200	4.29030700	-0.65106000
C	-3.75425200	4.60383100	0.64353000
H	-2.80700500	0.53440100	-1.44296400
N	-2.11998200	2.83842200	0.67314700
H	-0.35111100	1.19079200	-0.83942000
H	-4.06622600	5.57514900	1.00521500

[HL^2]$^-$

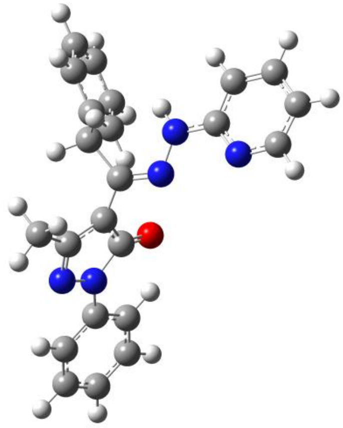

O	-1.28701800	-0.51191400	-1.27204700
N	1.39105400	-0.85566600	0.55991300
C	-0.78470300	-0.07307100	1.08021600
C	-1.58108900	-0.37337100	-0.07374700
C	0.66054800	0.09173500	1.05705300
C	-1.69567100	0.09217500	2.15504700
C	-1.38999100	0.38796400	3.59408900
H	-2.29961700	0.26557000	4.18550400
H	-1.02791700	1.41316700	3.73919300
H	-0.62056500	-0.28568400	3.98562400
N	-2.91490000	-0.37800400	0.44496300
N	-2.95207400	-0.10947400	1.80704200
C	-4.11055800	-0.60544900	-0.23538600
C	-4.13553300	-0.89295600	-1.61458700
H	-3.19600200	-0.93235500	-2.14575100
C	-5.33165400	-0.54784200	0.46442600
H	-5.30065600	-0.32726300	1.52169600
C	-6.53288600	-0.77141800	-0.19682900
H	-7.46100900	-0.72211700	0.36581800
C	-5.35063200	-1.11268500	-2.25486000
H	-5.34666000	-1.33356400	-3.31845700
C	-6.56007900	-1.05581900	-1.56243300
H	-7.50129200	-1.22950100	-2.07392800
C	1.23961100	1.43288700	1.49313800
H	2.17167500	1.28228300	2.05290100
H	0.52971400	1.90890500	2.17361300
C	2.35040200	3.49983100	0.54006600
H	2.81435800	3.64236500	1.51219500
C	1.50916100	2.39689400	0.33880600
C	0.92196100	2.22426100	-0.92221100
H	0.27949100	1.36925700	-1.11679700
C	2.00474900	4.23717300	-1.73065000
H	2.19476400	4.94536600	-2.53059900
C	2.59720300	4.41364600	-0.48093700
H	3.25226400	5.26054900	-0.30195000
C	1.17244900	3.14114000	-1.94384900
H	0.70891500	2.98973300	-2.91283900
N	2.72573100	-0.54294500	0.35326900
C	3.63920400	-1.49925900	-0.01701700
C	4.86932100	-1.07791000	-0.58320900
H	5.05364500	-0.02369700	-0.76230200
C	4.29268500	-3.68872600	-0.12871200
H	4.02432800	-4.72588300	0.06537100
C	5.81188600	-2.03099200	-0.90974100
H	6.75765900	-1.72828700	-1.34827000
C	5.53015900	-3.38373300	-0.68393200
H	2.95368100	0.40613800	0.07914900
N	3.36604900	-2.79130100	0.21030000
H	6.23908800	-4.16477200	-0.93064400

Complex 1

Zn	0.00000100	0.00000100	0.00000600
O	0.95971400	-0.73997300	1.65392600
O	-1.14798700	1.19776600	1.54989700
N	-2.59913500	-1.38894500	-0.46221000
N	-1.34993700	-1.58051600	0.19429000
N	1.94927900	-2.64713300	2.57272800
N	1.78460300	-4.02103700	2.55453800
C	3.06707300	-2.09258000	3.24296900
F	-8.29636800	0.18242100	1.85446100
C	0.12119800	-3.03763000	1.34558400
C	0.70633300	-4.25832000	1.84688500
C	-1.71805900	-4.66427300	-1.04793700
C	0.98687200	-2.00697800	1.83089900
C	-4.74744200	-0.30048100	-0.31417500
H	-4.68471400	-0.07678800	-1.37416800
C	-5.96140300	-0.19781700	1.77384600
C	-3.68434500	-0.97419300	0.31799200
C	-2.01153800	-3.95393600	0.26852000
H	-3.03270100	-3.57516200	0.22667600
H	-1.98449600	-4.67871200	1.08099500
C	3.02928800	-0.79174000	3.75836700
H	2.13096100	-0.19976900	3.65706900
C	-3.78760300	-1.25765700	1.68846300
H	-2.98470700	-1.77314200	2.19950600
F	-7.31177500	-0.38823900	3.71145400
C	-1.07293900	-2.80414900	0.58990900
C	-2.24815000	2.11682700	1.64577800
H	-2.08434000	2.82744500	2.46006500
H	-3.19139100	1.58795800	1.79276200
H	-2.28043000	2.66211500	0.70605900
C	4.21747600	-2.87222900	3.40533700
H	4.22200200	-3.88451900	3.02408800
C	0.29225300	-5.68933000	1.68158900
H	1.08260000	-6.32545800	2.08103700
H	0.12393600	-5.95443400	0.63612700
H	-0.62516500	-5.91770500	2.23366700
F	-7.02542500	1.60058300	2.89005600
C	-4.91611200	-0.86919500	2.40335000
H	-4.98198500	-1.09727300	3.46090100
C	-5.86493800	0.08206600	0.40549900
H	-6.67153400	0.60209800	-0.09881600
C	4.14800100	-0.27386800	4.40757400
H	4.10999200	0.73605300	4.80312600
C	5.29959500	-1.04225800	4.56146100
H	6.16594400	-0.63462700	5.07153000
C	-7.14229500	0.28856400	2.55313800
C	-1.04916100	-4.04530200	-2.10727600
H	-0.66984400	-3.03626600	-1.99209400
C	5.32282100	-2.34490500	4.06412200
H	6.20980800	-2.95812500	4.18665400
C	-0.85295700	-4.71978000	-3.31243300
H	-0.33113700	-4.22377800	-4.12535600
C	-1.32403700	-6.01878200	-3.48032800
H	-1.17068200	-6.54066700	-4.41933800
C	-2.18412200	-5.97233700	-1.22582300
H	-2.70663500	-6.46959800	-0.41312800
C	-1.99234300	-6.64443300	-2.42958000
H	-2.36270900	-7.65816100	-2.54528800
H	-2.44081200	-0.77196300	-1.25274500
H	-1.07954300	0.66646600	2.35038100
O	-0.95971200	0.73997500	-1.65391500
O	1.14799300	-1.19776200	-1.54988200
N	2.59914200	1.38894100	0.46221200
N	1.34994000	1.58051700	-0.19428200
N	-1.94927400	2.64713500	-2.57271700
N	-1.78459700	4.02103900	-2.55452500
C	-3.06707000	2.09258400	-3.24295700
F	8.29635700	-0.18245900	-1.85447800
C	-0.12119300	3.03763000	-1.34557600
C	-0.70632200	4.25832000	-1.84688100
C	1.71806200	4.66429100	1.04792700
C	-0.98686800	2.00697900	-1.83088900
C	4.74744200	0.30046300	0.31416900
H	4.68471600	0.07677100	1.37416300
C	5.96139400	0.19779000	-1.77385500
C	3.68434600	0.97418000	-0.31799400
C	2.01154400	3.95393600	-0.26852000

H	3.03270500	3.57516000	-0.22666900
H	1.98450800	4.67870500	-1.08100200
C	-3.02928800	0.79174400	-3.75835400
H	-2.13096300	0.19977000	-3.65705700
C	3.78760000	1.25764000	-1.68846700
H	2.98470400	1.77312800	-2.19950800
F	7.31176100	0.38820500	-3.71146700
C	1.07294200	2.80415000	-0.58990100
C	2.24816200	-2.11681600	-1.64576200
H	2.08435600	-2.82744000	-2.46004400
H	3.19139900	-1.58794100	-1.79274900
H	2.28044800	-2.66210000	-0.70603900
C	-4.21747200	2.87223500	-3.40532400
H	-4.22199500	3.88452500	-3.02407500
C	-0.29223600	5.68932900	-1.68158800
H	-1.08258300	6.32546000	-2.08103300
H	-0.12391400	5.95443400	-0.63612700
H	0.62518000	5.91770100	-2.23367000
F	7.02540500	-1.60061500	-2.89006800
C	4.91610500	0.86917200	-2.40335700
H	4.98197400	1.09724700	-3.46090900
C	5.86493300	-0.08209000	-0.40550800
H	6.67152800	-0.60212700	0.09880500
C	-4.14800300	0.27387300	-4.40755900
H	-4.10999700	-0.73604700	-4.80311000
C	-5.29959600	1.04226600	-4.56144500
H	-6.16594700	0.63463600	-5.07151200
C	7.14228100	-0.28859700	-2.55315100
C	1.04914900	4.04534100	2.10726900
H	0.66982200	3.03630700	1.99209600
C	-5.32281800	2.34491300	-4.06410600
H	-6.20980500	2.95813500	-4.18663700
C	0.85294000	4.71983500	3.31241500
H	0.33110900	4.22384800	4.12534000
C	1.32403000	6.01883600	3.48029700
H	1.17067200	6.54073400	4.41930000
C	2.18413200	5.97235500	1.22580000
H	2.70665200	6.46960200	0.41310100
C	1.99235100	6.64446700	2.42954700
H	2.36272600	7.65819300	2.54524600
H	2.44081900	0.77196000	1.25274800
H	1.07954500	-0.66646500	-2.35036700

Complex 2

Zn	-0.14749400	0.16439600	-0.81358400
C	0.56408100	3.96311600	2.37843900
C	0.52965400	-3.45416300	2.62534000
C	-0.57830800	3.26664600	2.79470800
H	-0.76901200	2.27675000	2.40985800
C	1.73522900	-3.23709300	1.94282700
H	1.77759700	-2.49423600	1.16201000
C	-1.45183700	3.86021400	3.70224900
H	-2.33032900	3.30826700	4.02309300
C	2.86222700	-3.98197400	2.28147200
H	3.78714900	-3.80471600	1.74053200
C	-1.20747600	5.13461500	4.20753000
H	-1.89315500	5.58663700	4.91712500
C	2.81650200	-4.93953300	3.29113100
H	3.70091900	-5.51399000	3.54779200
C	-0.06537900	5.81859400	3.79472100
H	0.14487500	6.81125800	4.18122000
C	1.61653800	-5.14738300	3.96893500
H	1.55958800	-5.88731100	4.76158700
C	0.81682400	5.24424400	2.88683400
H	1.70925000	5.76522000	2.56894200
C	0.47998400	-4.41626100	3.64453300
H	-0.45254100	-4.57413500	4.16728400
C	1.31875800	2.32205900	0.61033400
C	-0.85903100	-1.77038700	1.33914600
C	2.56183000	2.20909100	-0.10662300
C	-2.23676100	-1.38404000	1.47726100
C	3.37388300	3.28183200	0.41663200
C	-2.72688300	-2.18950300	2.56858900
C	4.77785400	3.71613100	0.10835600
H	5.03001300	4.54268000	0.77343800

H	4.89009700	4.07223000	-0.92114500
H	5.50977400	2.91924900	0.26897100
C	-4.08228300	-2.28157600	3.20816800
H	-4.07554100	-3.12309000	3.90167500
H	-4.88344800	-2.44416900	2.48191100
H	-4.33180600	-1.38419400	3.78413600
C	2.85014200	1.19098900	-1.06371500
C	-2.88991000	-0.41729500	0.65755100
C	1.35338300	-1.59938200	-2.69568200
C	-2.34160000	1.71720800	-2.13390200
C	1.72600900	-2.73071700	-3.45074700
H	2.77072600	-2.91522600	-3.67725100
C	-3.07233000	2.53407200	-3.02326400
H	-4.15192900	2.59998100	-2.93973900
C	0.73468900	-3.58165100	-3.89040100
H	0.99600100	-4.45984200	-4.47170600
C	-2.38082100	3.24802500	-3.97772000
H	-2.92016200	3.88779500	-4.66862500
C	-0.60584900	-3.30924500	-3.57928000
H	-1.40733200	-3.95916600	-3.90782200
C	-0.98330100	3.14538800	-4.04841500
H	-0.41013700	3.69817500	-4.78220600
C	-0.87474800	-2.18709100	-2.82093400
H	-1.88633500	-1.93466600	-2.51967300
C	-0.35330000	2.31069400	-3.14649200
H	0.72355100	2.17977800	-3.15225000
N	1.48462300	3.39678500	1.46431100
N	-0.64573700	-2.72823600	2.31533200
N	2.73493200	3.97178600	1.32849300
N	-1.79516400	-2.97701500	3.04488600
N	1.93238500	0.30067400	-1.38477900
N	-2.27953900	0.08686400	-0.39738900
N	2.31021700	-0.69699000	-2.28360100
N	-2.98797000	1.02986300	-1.13758200
N	0.07634200	-1.34454400	-2.38592400
N	-1.00809300	1.60518400	-2.20938700
O	0.25830600	1.64408000	0.53859300
O	0.02864800	-1.37241700	0.53665200
H	-3.97655800	0.86044500	-1.27574100
C	-4.29072500	0.06042900	1.00190400
H	-4.53201200	-0.22085300	2.02418300
H	-4.27198200	1.15558900	0.98879000
C	-5.39775500	-0.42793700	0.07545300
C	-5.38200800	-1.71867100	-0.46714300
C	-6.48220500	0.40680100	-0.22251700
C	-6.42342800	-2.16150600	-1.28008700
H	-4.54825200	-2.37960600	-0.25220600
C	-7.52495500	-0.03516300	-1.03477000
H	-6.51317100	1.41062700	0.19282900
C	-7.49852400	-1.32250500	-1.56635000
H	-6.39492200	-3.16671500	-1.68852000
H	-8.35767600	0.62683300	-1.25026500
H	-8.30891300	-1.66907200	-2.19924900
C	4.21885200	1.11180300	-1.71815600
H	4.73252000	2.06366500	-1.61179800
H	4.06909300	0.96860200	-2.79375600
C	5.12193000	-0.00024300	-1.19477700
C	5.09580500	-0.39654600	0.14740900
C	6.03258300	-0.62277000	-2.05806900
C	5.96114900	-1.38543500	0.61245100
H	4.39222400	0.06529100	0.83206600
C	6.89946200	-1.60971000	-1.59362500
H	6.06771900	-0.32574000	-3.10311600
C	6.86622600	-1.99387300	-0.25470800
H	5.92434000	-1.67905800	1.65640500
H	7.59986100	-2.07788000	-2.27809000
H	7.53899800	-2.76335100	0.10957600
H	3.24107300	-1.07963200	-2.16279900

Complex 3

Cu	0.00000000	0.00000000	0.00000000
O	1.80642200	0.12739200	-0.72427000
N	-0.06646200	-0.79966400	2.79890200

N	0.69796200	-0.92744700	1.61218500
N	3.73785400	-1.08017900	-1.24197200
N	4.43082100	-2.18731100	-0.77791000
C	4.16884600	-0.42902700	-2.42444900
F	0.12735000	3.72130100	7.24534500
C	2.64703500	-1.77980300	0.57484200
C	3.80401500	-2.59797100	0.29655900
C	1.27726500	-4.00142600	2.77524800
C	2.63592100	-0.81793200	-0.47743700
C	-0.84333100	0.77050700	4.46409300
H	-1.76836700	0.20800300	4.53420200
C	0.54549000	2.60562800	5.19951000
C	0.14769300	0.37094100	3.55063900
C	1.94826200	-2.63522700	2.87279500
H	1.55805800	-2.12212600	3.75174700
H	3.01774500	-2.77032100	3.03064300
C	3.94810600	0.93980900	-2.60976800
H	3.46642300	1.51783900	-1.83281600
C	1.33754100	1.10636000	3.47456700
H	2.10755700	0.82296000	2.76914400
F	2.03435300	4.10659600	6.26706500
C	1.73227500	-1.75005900	1.66240800
C	4.83348800	-1.17062400	-3.40556600
H	5.01647500	-2.22312400	-3.23432600
C	4.34696600	-3.79438800	1.01515100
H	5.12799000	-4.24465400	0.40216000
H	3.57544700	-4.54244200	1.20755800
H	4.79494200	-3.52587800	1.97722800
F	0.20375600	4.93846500	5.45276400
C	1.52796600	2.21427900	4.29339300
H	2.45414500	2.77311700	4.22696300
C	-0.64466800	1.87483700	5.27471200
H	-1.41747300	2.16884600	5.97541200
C	4.36675900	1.55082000	-3.78998500
H	4.19163500	2.61274600	-3.92840300
C	5.01769900	0.81430900	-4.77711500
H	5.34562500	1.29631800	-5.69195800
C	0.72952300	3.83253500	6.03859900
C	0.20536000	-4.26101600	1.91829400
H	-0.16248900	-3.48334200	1.25797600
C	5.25636500	-0.54458700	-4.57324500
H	5.77289300	-1.12554600	-5.33037800
C	-0.39847100	-5.51802100	1.89376800
H	-1.22557600	-5.70122400	1.21528500
C	0.05598900	-6.53304600	2.73018700
H	-0.41374700	-7.51094700	2.70972800
C	1.72820100	-5.03099300	3.61003500
H	2.56208400	-4.84829400	4.28228800
C	1.12335800	-6.28396400	3.59163200
H	1.48915900	-7.06780200	4.24727900
H	-1.04532600	-0.91522500	2.55944700
O	-1.80642200	-0.12739200	0.72427000
N	0.06646200	0.79966400	-2.79890200
N	-0.69796200	0.92744700	-1.61218500
N	-3.73785400	1.08017900	1.24197200
N	-4.43082100	2.18731100	0.77791000
C	-4.16884600	0.42902700	2.42444900
F	-0.12735000	-3.72130100	-7.24534500
C	-2.64703500	1.77980300	-0.57484200
C	-3.80401500	2.59797100	-0.29655900
C	-1.27726500	4.00142600	-2.77524800
C	-2.63592100	0.81793200	0.47743700
C	0.84333100	-0.77050700	-4.46409300
H	1.76836700	-0.20800300	-4.53420200
C	-0.54549000	-2.60562800	-5.19951000
C	-0.14769300	-0.37094100	-3.55063900
C	-1.94826200	2.63522700	-2.87279500
H	-1.55805800	2.12212600	-3.75174700
H	-3.01774500	2.77032100	-3.03064300
C	-3.94810600	-0.93980900	2.60976800
H	-3.46642300	-1.51783900	1.83281600
C	-1.33754100	-1.10636000	-3.47456700
H	-2.10755700	-0.82296000	-2.76914400
F	-2.03435300	-4.10659600	-6.26706500
C	-1.73227500	1.75005900	-1.66240800
C	-4.83348800	1.17062400	3.40556600
H	-5.01647500	2.22312400	3.23432600
C	-4.34696600	3.79438800	-1.01515100

H	-5.12799000	4.24465400	-0.40216000
H	-3.57544700	4.54244200	-1.20755800
H	-4.79494200	3.52587800	-1.97722800
F	-0.20375600	-4.93846500	-5.45276400
C	-1.52796600	-2.21427900	-4.29339300
H	-2.45414500	-2.77311700	-4.22696300
C	0.64466800	-1.87483700	-5.27471200
H	1.41747300	-2.16884600	-5.97541200
C	-4.36675900	-1.55082000	3.78998500
H	-4.19163500	-2.61274600	3.92840300
C	-5.01769900	-0.81430900	4.77711500
H	-5.34562500	-1.29631800	5.69195800
C	-0.72952300	-3.83253500	-6.03859900
C	-0.20536000	4.26101600	-1.91829400
H	0.16248900	3.48334200	-1.25797600
C	-5.25636500	0.54458700	4.57324500
H	-5.77289300	1.12554600	5.33037800
C	0.39847100	5.51802100	-1.89376800
H	1.22557600	5.70122400	-1.21528500
C	-0.05598900	6.53304600	-2.73018700
H	0.41374700	7.51094700	-2.70972800
C	-1.72820100	5.03099300	-3.61003500
H	-2.56208400	4.84829400	-4.28228800
C	-1.12335800	6.28396400	-3.59163200
H	-1.48915900	7.06780200	-4.24727900
H	1.04532600	0.91522500	-2.55944700

Complex 4

Cu	0.25328500	-1.00628300	0.28406400
C	-0.93441100	2.93885800	3.04681200
C	-0.92850300	2.94793900	-2.82794900
C	0.29292000	3.28415600	2.47278900
H	0.62324000	2.79690200	1.56590100
C	-2.03088200	2.07977500	-2.83025100
H	-1.95273500	1.12299900	-2.33789800
C	1.07574900	4.25704600	3.08529900
H	2.02693400	4.52596100	2.63822800
C	-3.20763900	2.46093900	-3.47026000
H	-4.05247500	1.77847600	-3.45985300
C	0.64534400	4.89536500	4.24746000
H	1.26092600	5.65851200	4.71163600
C	-3.31241500	3.69093700	-4.11449100
H	-4.23359800	3.97687300	-4.61223000
C	-0.58142700	4.54838800	4.80735000
H	-0.92469000	5.03362300	5.71513100
C	-2.21294300	4.54777300	-4.11235500
H	-2.27181400	5.51074500	-4.61097700
C	-1.37224000	3.56695800	4.21671300
H	-2.31319400	3.27332300	4.66681100
C	-1.03037700	4.18783000	-3.47687100
H	-0.17620800	4.85003400	-3.47265900
C	-1.39303100	0.77141500	1.83478700
C	0.59697600	1.47985300	-1.43335000
C	-2.62841100	0.03304600	1.58363900
C	1.95406400	1.69632000	-0.97925000
C	-3.65355900	0.83836900	2.02823300
C	2.32028000	2.97629900	-1.54582600
C	-5.14209000	0.72245800	1.98871200
H	-5.60478500	1.71387800	1.98578900
H	-5.52589900	0.19525400	2.86809300
H	-5.48131300	0.19171800	1.10165100
C	3.58851800	3.78061900	-1.48504900
H	3.46427800	4.65195400	-2.12884500
H	4.46184800	3.22288200	-1.83453800
H	3.80811900	4.14462100	-0.47565800
C	-2.67755800	-1.30354600	1.01104400
C	2.70881800	0.78447700	-0.18579800
C	-0.63919000	-3.39955600	-0.82656300
C	2.64266700	-2.20033300	1.75050500
C	-0.64766700	-4.64387700	-1.51336500
H	-1.52120600	-5.27572300	-1.41076000
C	3.56146800	-3.12763700	2.28776000
H	4.61461100	-3.06928300	2.03204900
C	0.42484100	-4.99509900	-2.29630800
H	0.41815700	-5.94071400	-2.83013100
C	3.08520400	-4.09615000	3.14735500

H	3.76963800	-4.82093300	3.57672100
C	1.52576000	-4.12422900	-2.41584200
H	2.37882100	-4.36498200	-3.03813200
C	1.71970500	-4.13687900	3.45890900
H	1.31233100	-4.88396400	4.12933600
C	1.48562100	-2.94045000	-1.70891900
H	2.30016700	-2.22509700	-1.75451400
C	0.89824100	-3.19230000	2.86798000
H	-0.17199100	-3.18738800	3.05445400
N	-1.75606000	1.96609900	2.42086300
N	0.28327500	2.61072600	-2.18278600
N	-3.13302300	1.97337200	2.63748500
H	-3.55869700	2.86312100	2.39190400
N	1.34199100	3.49870600	-2.24105900
N	-1.60884900	-1.80097900	0.42938300
N	2.20857700	-0.36761300	0.23412400
N	-1.72311100	-3.02763500	-0.11897000
N	3.10186900	-1.19010500	0.93090200
N	0.45469000	-2.58676400	-0.92517400
N	1.33796200	-2.24268700	2.03106400
O	-0.21118700	0.44920000	1.64335500
O	-0.18418500	0.52437800	-1.24076800
H	3.98683200	-1.36433100	0.47116900
C	4.14432000	1.11982600	0.18883700
H	4.30300700	2.19129500	0.10542000
H	4.27610600	0.86897500	1.24497700
C	5.21033400	0.39618400	-0.62571000
C	5.04538800	0.14771400	-1.99390800
C	6.40414200	-0.00831000	-0.01501800
C	6.04768600	-0.48347000	-2.72866000
H	4.12573100	0.44775200	-2.48564900
C	7.40802600	-0.63704200	-0.74896000
H	6.54961300	0.17587700	1.04612500
C	7.23242800	-0.87679400	-2.11014600
H	5.90038100	-0.66658700	-3.78825700
H	8.32653600	-0.94000900	-0.25611900
H	8.01168700	-1.36822300	-2.68354600
C	-3.93777300	-2.13637400	1.09581600
H	-4.47354200	-1.89281500	2.01726400
H	-3.61615400	-3.17858700	1.17635000
C	-4.89842900	-2.03736100	-0.08242500
C	-4.48114500	-1.64157300	-1.35634200
C	-6.24229800	-2.38632600	0.09885500
C	-5.38446700	-1.59494000	-2.41744700
H	-3.44293600	-1.38061800	-1.52384900
C	-7.14653100	-2.34112100	-0.95955200
H	-6.58478400	-2.70079000	1.08180300
C	-6.71953900	-1.94257300	-2.22466500
H	-5.03791900	-1.29091400	-3.40033800
H	-8.18391100	-2.61652100	-0.79515200
H	-7.42051800	-1.90516900	-3.05252600

Complex 5

Cu	-0.00001100	-0.00000500	0.00001700
O	1.77975300	-0.71596900	0.03309800
C	2.81313700	-0.02585600	-0.23661000
C	2.92157800	1.33356100	-0.68399200
C	1.78170700	2.12743700	-0.90485500
N	4.09366900	-0.49612000	-0.14520400
N	5.01733800	0.48111200	-0.50399700
C	4.56319300	-1.78397800	0.23244000
C	5.09878900	2.77544900	-1.23484500
H	6.16685500	2.57134700	-1.15764800
H	4.86288300	3.63162200	-0.59809100
H	4.88216300	3.05774100	-2.26976100
C	3.69930700	-2.75183100	0.76053700
H	2.65071700	-2.52936600	0.88727100
C	4.20639500	-3.99934600	1.11600800
H	3.52914800	-4.74340200	1.52316100
C	5.92305300	-2.07565900	0.07199500
H	6.57971000	-1.31719900	-0.32995800
C	5.55698900	-4.29696500	0.95759900
H	5.94136000	-5.27203000	1.23829400
C	6.40941900	-3.32615000	0.43553300
H	7.46574000	-3.54004400	0.30703900
C	1.70165000	4.56976200	-0.23873600

C	0.63577800	4.48914700	0.66562500
H	-0.08650100	3.68616700	0.57170400
C	2.61831300	5.61617300	-0.10221400
H	3.44350000	5.70344500	-0.80292200
C	2.48068300	6.56004200	0.91451600
H	3.20273700	7.36574000	1.00222000
C	1.85014400	3.56790400	-1.37810100
H	1.02564700	3.69428800	-2.08648900
H	2.77635800	3.75966600	-1.91743600
C	0.50018800	5.42978100	1.68310100
H	-0.33281200	5.35293400	2.37467400
C	1.42162300	6.46782300	1.81282500
H	1.31246500	7.19999300	2.60638600
C	4.34259700	1.55297600	-0.81922600
O	-1.77977600	0.71595400	-0.03307300
C	-2.81316100	0.02583800	0.23662600
C	-2.92160000	-1.33357700	0.68401200
C	-1.78172800	-2.12744600	0.90489600
N	-4.09369300	0.49610300	0.14522500
N	-5.01736100	-0.48112300	0.50403600
C	-4.56322100	1.78396200	-0.23241000
C	-5.09881700	-2.77550400	1.23474500
H	-6.16688300	-2.57139800	1.15754800
H	-4.86290800	-3.63165100	0.59795700
H	-4.88220000	-3.05783800	2.26965100
C	-3.69934400	2.75182100	-0.76051300
H	-2.65075600	2.52935900	-0.88726100
C	-4.20644000	3.99933600	-1.11597000
H	-3.52920100	4.74339700	-1.52312700
C	-5.92308000	2.07563900	-0.07194600
H	-6.57973100	1.31717500	0.33001100
C	-5.55703300	4.29695100	-0.95754300
H	-5.94141100	5.27201600	-1.23822800
C	-6.40945400	3.32613100	-0.43547100
H	-7.46577400	3.54002100	-0.30696300
C	-1.70161700	-4.56974600	0.23870700
C	-0.63573200	-4.48906500	-0.66563300
H	0.08651800	-3.68606200	-0.57167200
C	-2.61824400	-5.61618300	0.10213400
H	-3.44343900	-5.70350700	0.80282500
C	-2.48056400	-6.56001200	-0.91462600
H	-3.20258900	-7.36573200	-1.00237100
C	-1.85016000	-3.56792700	1.37809900
H	-1.02567700	-3.69431800	2.08650100
H	-2.77638400	-3.75971900	1.91740600
C	-0.50009300	-5.42965800	-1.68313800
H	0.33291600	-5.35276000	-2.37469500
C	-1.42149100	-6.46772700	-1.81291300
H	-1.31229500	-7.19986600	-2.60649800
C	-4.34262200	-1.55301300	0.81918800
O	-0.60157700	-1.69597000	0.71412000
O	0.60155600	1.69596000	-0.71408400

Complex [Zn(HL^1)$_2$]

Zn	0.00001500	0.00018400	-0.00024200
O	1.25671300	-0.17886100	-1.58307100
N	-1.87403600	2.23387300	-0.31948700
N	-0.56787400	1.80474200	-0.68845500
N	2.95090000	0.88600400	-2.78633100
N	3.35049600	2.17419600	-3.10815000
C	3.76216300	-0.21174400	-3.16600000
F	-7.40514800	0.45349300	-2.85236400
C	1.39957900	2.25146200	-1.93680900
C	2.44012100	2.98140000	-2.62625900
C	0.09787900	5.05278600	-0.42463800
C	1.80053300	0.87952400	-2.04540300
C	-4.20213200	1.57609300	-0.39524700
H	-4.34548100	2.03204100	0.57856500
C	-5.10340100	0.40008600	-2.30432800
C	-2.92470600	1.57491900	-0.98344300
C	-0.31237100	4.09878000	-1.54158000
H	-1.40173500	4.08250700	-1.59263600
H	0.04796400	4.48509500	-2.49400300
C	3.19787800	-1.46708800	-3.41687600
H	2.12761000	-1.59992400	-3.34064500
C	-2.75620400	0.97890800	-2.23995900

H	-1.78073700	0.96645600	-2.70823800
F	-6.05638600	-0.46638100	-4.29337900
C	0.17030300	2.67457700	-1.36581700
C	5.14050700	-0.02383800	-3.30503200
H	5.55629800	0.95970200	-3.13093800
C	2.62610100	4.45284400	-2.83600200
H	3.62731100	4.62355900	-3.23224700
H	2.51388700	5.01841000	-1.90893200
H	1.91131700	4.85778000	-3.55950000
F	-6.53265500	-1.48590600	-2.43682200
C	-3.84072600	0.39457900	-2.88854200
H	-3.69828600	-0.06031000	-3.86157400
C	-5.27386500	0.99489800	-1.04864700
H	-6.25220600	1.00410200	-0.58179400
C	4.02078000	-2.53210100	-3.77666300
H	3.57692900	-3.50325100	-3.97094200
C	5.39671000	-2.35415900	-3.90416500
H	6.03182000	-3.18613400	-4.18944400
C	-6.26552600	-0.26797100	-2.97258500
C	0.50408000	4.61843600	0.83908300
H	0.56994600	3.55751400	1.05446700
C	5.94820000	-1.09393200	-3.67537800
H	7.01707000	-0.94032500	-3.78305200
C	0.83751900	5.53581300	1.83557900
H	1.15822800	5.17650700	2.80826100
C	0.76459400	6.90258200	1.58608200
H	1.02536800	7.61559100	2.36126700
C	0.03148700	6.43065400	-0.66488800
H	-0.28037400	6.78864200	-1.64226700
C	0.35795700	7.34775800	0.32903100
H	0.29945400	8.41123400	0.12001900
H	-1.97070200	2.15412300	0.68688100
O	-1.25641400	0.17942800	1.58273400
N	1.87382000	-2.23371900	0.31870500
N	0.56771400	-1.80448100	0.68776100
N	-2.95048700	-0.88518200	2.78637500
N	-3.35021100	-2.17331400	3.10827500
C	-3.76150500	0.21268100	3.16623700
F	7.40513900	-0.45565500	2.85281700
C	-1.39958400	-2.25088200	1.93647100
C	-2.44008600	-2.98066000	2.62615500
C	-0.09855100	-5.05248100	0.42433000
C	-1.80029100	-0.87888400	2.04517900
C	4.20211200	-1.57673200	0.39507000
H	4.34546200	-2.03249700	-0.57882700
C	5.10345800	-0.40147700	2.30457500
C	2.92459000	-1.57527100	0.98305700
C	0.31209700	-4.09843300	1.54109100
H	1.40147200	-4.08231300	1.59189000
H	-0.04806400	-4.48461300	2.49363600
C	-3.19703900	1.46803200	3.41669100
H	-2.12679700	1.60079200	3.34000100
C	2.75608400	-0.97952900	2.23969500
H	1.78055300	-0.96686800	2.70783000
F	6.05646300	0.46404300	4.29402800
C	-0.17044500	-2.67418300	1.36530700
C	-5.13980800	0.02488900	3.30586100
H	-5.55573200	-0.95864800	3.13207500
C	-2.62627400	-4.45207800	2.83588900
H	-3.62745700	-4.62264600	3.23226200
H	-2.51426100	-5.01764100	1.90879400
H	-1.91145900	-4.85714300	3.55928500
F	6.53316900	1.48411100	2.43788500
C	3.84069800	-0.39571600	2.88859600
H	3.69825200	0.05898000	3.86171800
C	5.27392800	-0.99603400	1.04877200
H	6.25234200	-1.00544500	0.58207700
C	-4.01972900	2.53315000	3.77665300
H	-3.57573600	3.50430200	3.97059400
C	-5.39561800	2.35531800	3.90474900
H	-6.03055700	3.18737800	4.19015900
C	6.26568600	0.26605000	2.97318500
C	-0.50495400	-4.61818300	-0.83934200
H	-0.57068600	-3.55727200	-1.05481200
C	-5.94728000	1.09509100	3.67638100
H	-7.01611500	0.94156700	3.78451500
C	-0.83876400	-5.53560300	-1.83567400
H	-1.15962500	-5.17633800	-2.80832200

C	-0.76601200	-6.90235900	-1.58606100
H	-1.02707600	-7.61540000	-2.36112100
C	-0.03232800	-6.43033700	0.66470000
H	0.27969000	-6.78828200	1.64204400
C	-0.35917000	-7.34748300	-0.32905800
H	-0.30079500	-8.41094900	-0.11995700
H	1.97055100	-2.15338300	-0.68761500

Complex [Zn(HL^3)$_2$] (Tetrahedral)

Zn	0.01042200	-0.37573400	-0.07571900
O	-1.56147400	-1.31772400	-0.76624400
N	-0.27739000	1.71105100	1.95460300
N	-0.99805400	0.63127300	1.32990800
N	-3.77546800	-1.99020700	-0.50330700
N	-4.84902800	-1.75046400	0.33563200
C	-3.90901700	-2.96095900	-1.53031600
C	-3.08828500	-0.45312900	0.97302800
C	-4.46065900	-0.84425100	1.19708000
C	-3.66554200	2.64958900	1.99920600
C	-2.68101500	-1.24606100	-0.14800100
C	-2.90513200	1.48713300	2.62107600
H	-2.10456300	1.89752500	3.23616000
H	-3.57375100	0.92872700	3.27565500
C	-3.04338400	-2.98701100	-2.63028300
H	-2.25095400	-2.25827400	-2.70780000
C	-2.28105100	0.53348100	1.62101700
C	-4.94560200	-3.89737400	-1.43869700
H	-5.61765500	-3.85408700	-0.59283900
C	-5.45616800	-0.41104500	2.23028200
H	-6.38646700	-0.95254700	2.05681900
H	-5.66743900	0.65905700	2.17851800
H	-5.11912500	-0.63899100	3.24649800
C	-3.21469500	-3.95597400	-3.61603300
H	-2.53976100	-3.96694700	-4.46615500
C	-4.24015500	-4.89351600	-3.52714300
H	-4.36760200	-5.64278800	-4.30157600
C	-3.37578300	3.13669500	0.72248800
H	-2.61670600	2.65243200	0.11763500
C	-5.10530100	-4.85337300	-2.43516600
H	-5.91361300	-5.57323000	-2.35372800
C	-4.05625700	4.23938700	0.20890000
H	-3.81799800	4.59952400	-0.78693200
C	-5.03666100	4.87597000	0.96477500
H	-5.56695200	5.73347900	0.56350100
C	-4.65473200	3.29646100	2.74943800
H	-4.89337500	2.93369400	3.74569900
C	-5.33359200	4.40001000	2.24050200
H	-6.09742900	4.88565000	2.83971700
H	0.12126500	2.24271800	1.18451400
O	1.06875300	1.08033500	-0.86633400
N	1.24156100	-2.96091100	0.64389000
N	1.52528100	-1.66228500	0.08563900
N	3.10328500	1.88285700	-1.66395000
N	4.41903000	1.47097600	-1.77354100
C	2.75754300	3.19941400	-2.06751700
C	3.18088000	-0.19845600	-0.83639400
C	4.47588500	0.25275000	-1.29735900
C	4.62030900	-2.32482000	1.31008800
C	2.31715900	0.91485700	-1.09842500
C	3.83132500	-2.50823800	0.02061100
H	3.33235200	-3.47362700	0.07787600
H	4.52043300	-2.55060700	-0.82204100
C	1.43370700	3.55356900	-2.35341200
H	0.64925300	2.81744000	-2.26491000
C	2.78616000	-1.43838300	-0.24354000
C	3.77583800	4.15133300	-2.19555400
H	4.79572800	3.85841700	-1.98796600
C	5.81100200	-0.42795900	-1.33530100
H	6.54656400	0.28663700	-1.70546600
H	6.13013300	-0.77671100	-0.35148000
H	5.81415000	-1.28780500	-2.01284600
C	1.14228800	4.85624700	-2.75033800
H	0.11328300	5.12172600	-2.97165100
C	2.15009800	5.80875300	-2.87428900
H	1.91350200	6.82074200	-3.18608800
C	4.15777200	-1.54836100	2.37414800

H	3.22285400	-1.00705300	2.28129700
C	3.46708400	5.44543300	-2.59854200
H	4.26514600	6.17474400	-2.69506400
C	4.89056300	-1.44937700	3.55621700
H	4.51524500	-0.83616200	4.36957800
C	6.09614900	-2.13044600	3.69627600
H	6.66592000	-2.05241000	4.61650000
C	5.83512200	-3.00441600	1.46161600
H	6.21156300	-3.61454600	0.64491100
C	6.56657500	-2.91147300	2.64183800
H	7.50607000	-3.44688100	2.73653700
H	0.56044400	-2.79519100	1.37800800
C	0.63061500	-3.84521200	-0.36271700
H	1.38519600	-4.12549500	-1.10104500
H	0.30416100	-4.75405000	0.14804000
H	-0.22185300	-3.39443300	-0.87988500
C	0.82169100	1.17292100	2.75614000
H	1.47209900	1.99873300	3.05040000
H	0.42444400	0.70484900	3.65951500
H	1.42160300	0.42584100	2.21597200

Complex $[Zn(HL^3)_2]$ (Square planar)

Zn	0.00000000	0.00000000	0.00000000
O	1.98349600	0.12290700	-0.36880400
N	-1.59334500	2.12113900	-1.31881400
N	-0.26526800	1.82062400	-0.85174100
N	3.90314000	1.45307700	-0.32282500
N	4.24359200	2.79308000	-0.39534400
C	4.91069500	0.49258900	-0.05894300
C	1.99300300	2.56208300	-0.66449700
C	3.13038500	3.45087300	-0.60881900
C	-0.24663600	5.10122600	-0.23597000
C	2.55136400	1.26576700	-0.44910200
C	0.15962700	4.18285300	-1.38168100
H	-0.70408100	4.06624100	-2.03533900
H	0.95027200	4.65786800	-1.96078100
C	4.78780900	-0.82454900	-0.51434600
H	3.92190200	-1.11428400	-1.09286500
C	0.61460500	2.80050500	-0.94999600
C	6.05134800	0.88177700	0.65072300
H	6.14180300	1.91035300	0.97368100
C	3.22345900	4.94053100	-0.73950200
H	4.23851700	5.24625600	-0.48428500
H	2.52448900	5.45834600	-0.08024900
H	3.02452900	5.27679700	-1.76217100
C	5.79269700	-1.74717300	-0.23346600
H	5.68894500	-2.76739700	-0.58870200
C	6.92603000	-1.36735400	0.48154600
H	7.70707400	-2.08998200	0.69360100
C	-0.72441100	4.61973800	0.98527500
H	-0.78144400	3.55102100	1.15893900
C	7.05177200	-0.04768500	0.91315200
H	7.93435900	0.26325900	1.46303900
C	-1.12928500	5.49831700	1.98927200
H	-1.49386900	5.10257600	2.93212600
C	-1.06840700	6.87425700	1.78782300
H	-1.38317700	7.55723200	2.57019900
C	-0.18900100	6.48712600	-0.42633800
H	0.17958300	6.88210600	-1.36914900
C	-0.59734000	7.36666500	0.57209200
H	-0.54304600	8.43733400	0.40145000
H	-2.21117600	1.70588600	-0.62810300
O	-1.98349600	-0.12290700	0.36880400
N	1.59334500	-2.12113900	1.31881400
N	0.26526800	-1.82062400	0.85174100
N	-3.90314000	-1.45307700	0.32282500
N	-4.24359200	-2.79308000	0.39534400
C	-4.91069500	-0.49258900	0.05894300
C	-1.99300300	-2.56208300	0.66449700
C	-3.13038500	-3.45087300	0.60881900
C	0.24663600	-5.10122600	0.23597000
C	-2.55136400	-1.26576700	0.44910200
C	-0.15962700	-4.18285300	1.38168100
H	0.70408100	-4.06624100	2.03533900
H	-0.95027200	-4.65786800	1.96078100
C	-4.78780900	0.82454900	0.51434600

H	-3.92190200	1.11428400	1.09286500
C	-0.61460500	-2.80050500	0.94999600
C	-6.05134800	-0.88177700	-0.65072300
H	-6.14180300	-1.91035300	-0.97368100
C	-3.22345900	-4.94053100	0.73950200
H	-4.23851700	-5.24625600	0.48428500
H	-2.52448900	-5.45834600	0.08024900
H	-3.02452900	-5.27679700	1.76217100
C	-5.79269700	1.74717300	0.23346600
H	-5.68894500	2.76739700	0.58870200
C	-6.92603000	1.36735400	-0.48154600
H	-7.70707400	2.08998200	-0.69360100
C	0.72441100	-4.61973800	-0.98527500
H	0.78144400	-3.55102100	-1.15893900
C	-7.05177200	0.04768500	-0.91315200
H	-7.93435900	-0.26325900	-1.46303900
C	1.12928500	-5.49831700	-1.98927200
H	1.49386900	-5.10257600	-2.93212600
C	1.06840700	-6.87425700	-1.78782300
H	1.38317700	-7.55723200	-2.57019900
C	0.18900100	-6.48712600	0.42633800
H	-0.17958300	-6.88210600	1.36914900
C	0.59734000	-7.36666500	-0.57209200
H	0.54304600	-8.43733400	-0.40145000
H	2.21117600	-1.70588600	0.62810300
C	1.83534800	-1.41843400	2.58127900
H	1.24612300	-1.87156200	3.38168300
H	2.89414400	-1.51777700	2.82781800
H	1.58699500	-0.34660000	2.53504800
C	-1.83534800	1.41843400	-2.58127900
H	-2.89414400	1.51777700	-2.82781800
H	-1.24612300	1.87156200	-3.38168300
H	-1.58699500	0.34660000	-2.53504800

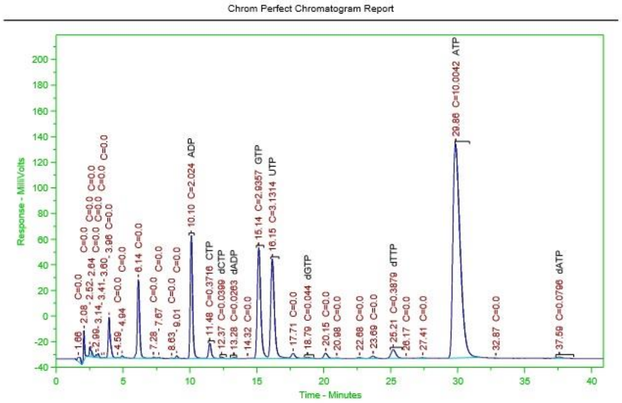

A

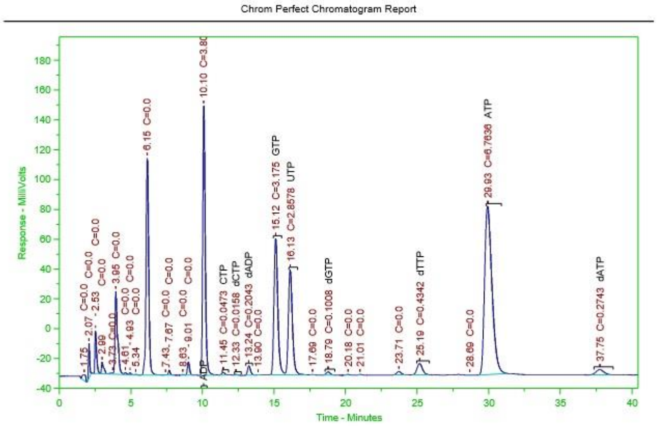

B

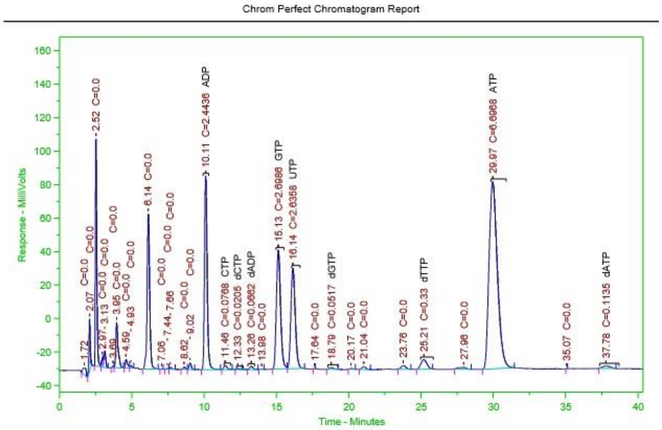

C

Figure S47. HPLC chromatographs showing NTPs, dNTPs, NDPs and dNDPs. From the top: A, non-treated cells; B, cells treated with H_2L^1 (5 uM); and C, cells treated with 1 (5 uM).

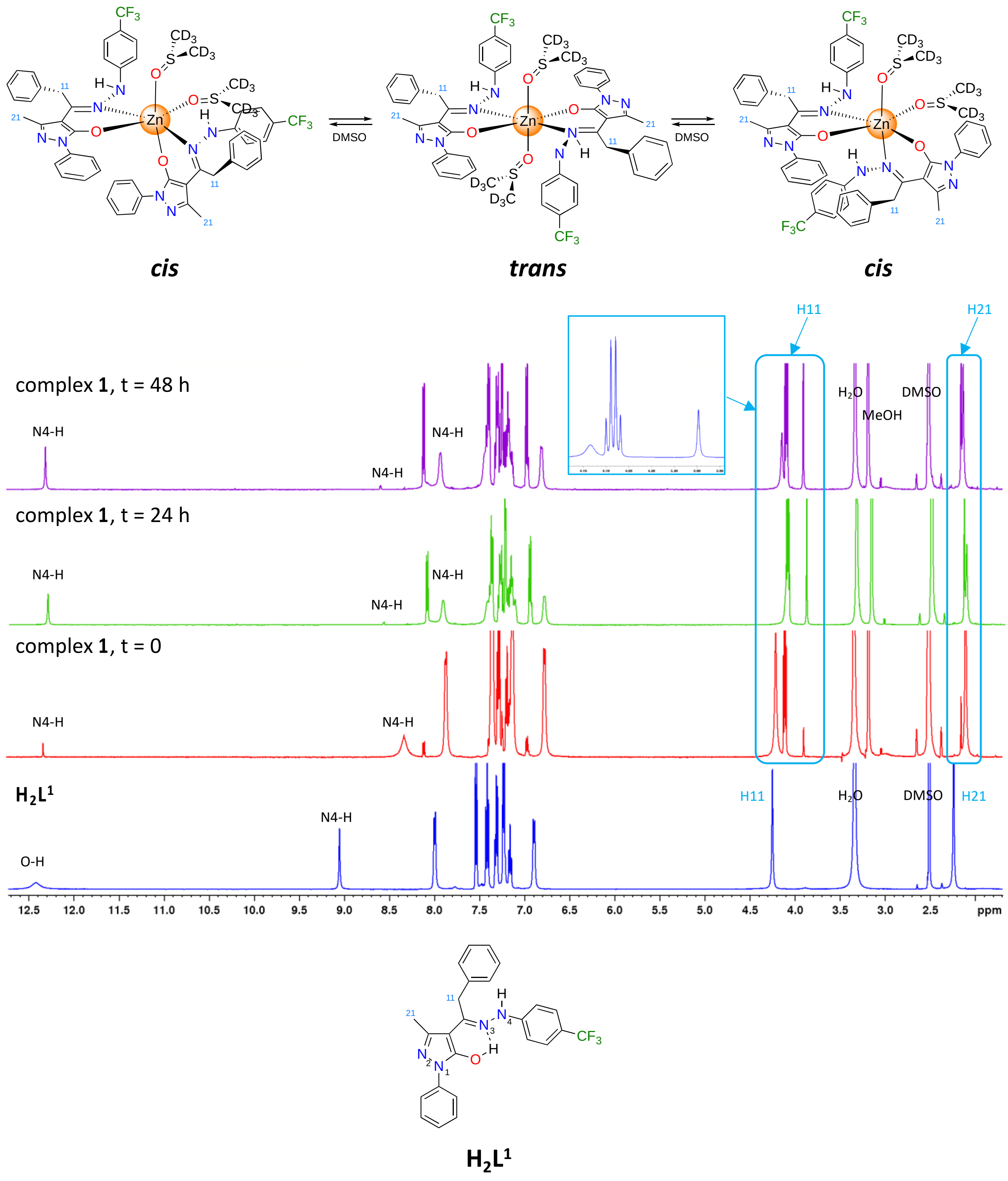

Figure S48. ^{1}H NMR spectra in DMSO-d6 of [Zn(HL^{1})$_2$(MeOH)$_2$] (**1**) at t = 0, 24 h and 48 h.

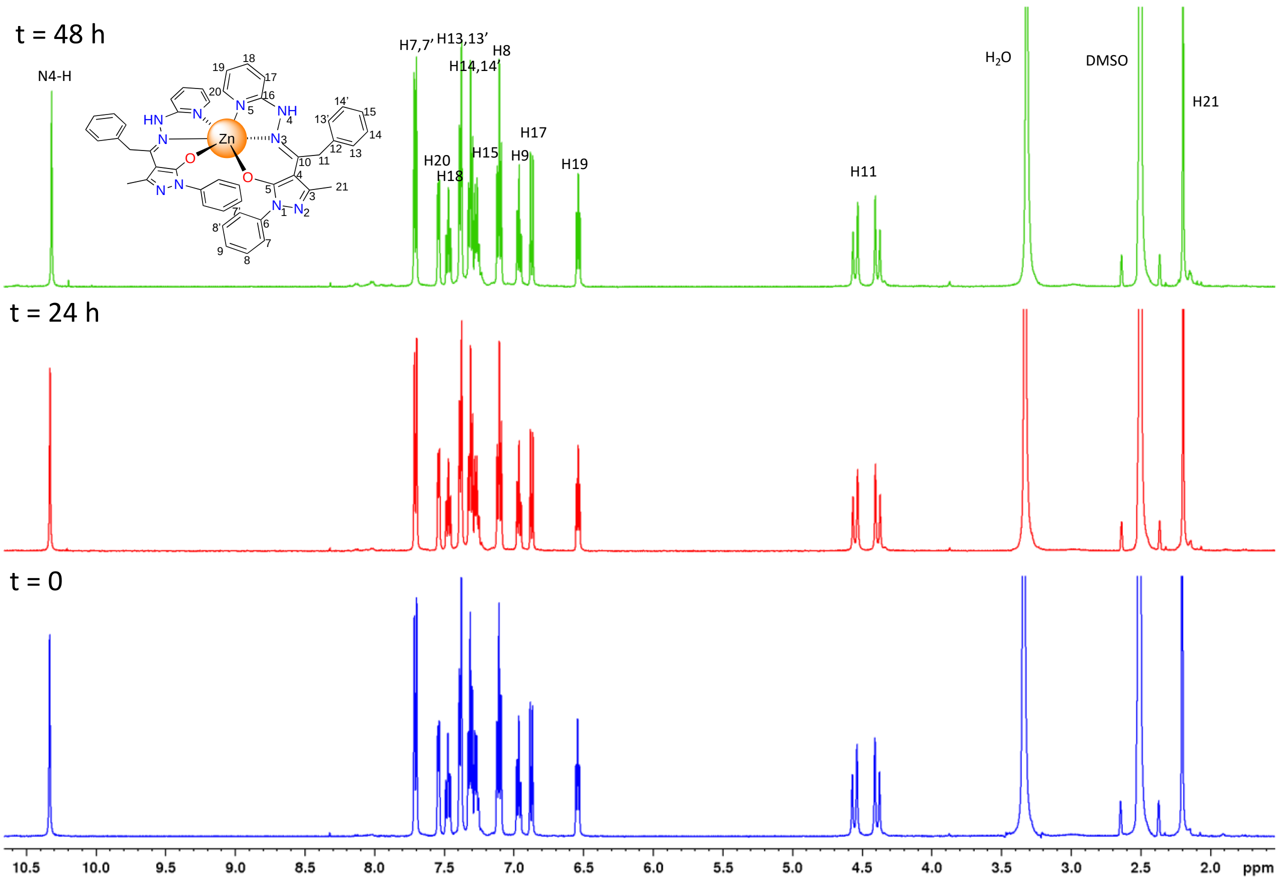

Figure S49. ^{1}H NMR spectra in DMSO-d$_6$ of [Zn(HL^2)$_2$] (**2**) at t = 0, 24 h and 48 h.
